# River pollution by priority chemical substances under the Water Framework Directive: A provisional pan-European assessment

**DOI:** 10.1016/j.scitotenv.2018.12.354

**Published:** 2019-04-20

**Authors:** Alberto Pistocchi, Chiara Dorati, Alberto Aloe, Antoni Ginebreda, Rafael Marcé

**Affiliations:** a European Commission, DG Joint Research Centre, Directorate D – Sustainable Resources, Via E. Fermi, 2749, 21027 Ispra, VA, Italy; b ARHS Italia - external consultant for the European Commission DG JRC - Directorate D Sustainable Resources, Via E. Fermi, 2749, 21027 Ispra, VA, Italy; c IDAEA-CSIC, Barcelona, Spain; d Catalan Institute for Water Research (ICRA), Emili Grahit 101, 17003 Girona, Spain

**Keywords:** Water Framework Directive, Priority substances, IPChem, Emission inventorying, Inverse modelling

## Abstract

In this paper, we build a preliminary inventory of dissolved phase water emissions of 36 of the 45 chemical priority substances under the European Union's Water Framework Directive. For point sources, we consider the European Pollutant Release and Transfer Register (E-PRTR) containing reported emissions from major industrial facilities. We consider all other sources as diffuse, and we estimate European average chemical emission factors from available measurements of dissolved phase concentrations, assuming simple emission patterns such as population and agricultural land. The emission inventory enables modelling concentrations, which have been compared with independent measurements. Due to the way they are estimated, they cannot withstand a point-by-point comparison. However, predicted concentrations exhibit a frequency distribution and order of magnitude compatible with observations, and match a fair proportion of independently reported exceedances of environmental quality standards for many of the substances studied.

While apparently a preliminary picture based on crude simplifications, our representation suggests that simple drivers such as population and agriculture are useful to describe chemical pollution at European scale.

From our preliminary inventory, E-PRTR industrial point emissions seem to account for a relatively small share of total emissions. Consequently, apart from specific measures such as upgrades to urban wastewater treatment plants in certain high impact areas, the management of priority substances may require a more strategic approach to emission control, addressing chemical use across sectors and the management of out-phased, legacy chemicals. At the same time, we advocate that improving emission inventories requires monitoring data reflecting the variability of emission patterns across Europe, as presently available monitoring data do not enable a catchment-specific estimation of emissions.

## Highlights

• Emissions of 36 priority water pollutants in Europe estimated from monitoring data by inverse modelling;
• Estimates reasonably match observations, suggesting emissions may, as a first approximation, be assumed uniform in the EU;
• Large industrial point source emissions generally important at a local level, but diffuse emissions dominate elsewhere;
• The paper demonstrates the feasibility of emission inventorying at continental scale
• More targeted monitoring needed to better understand emission patterns, hence to plan river basin management measures.

## Graphical abstract

**Figure:**
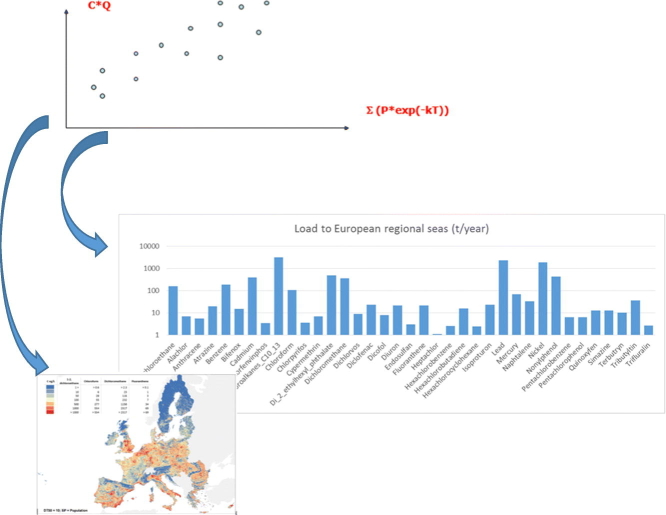

## 1 Introduction

Chemical water pollution is a key issue in river basin management. In the European Union (EU), the Water Framework Directive (WFD) 60/2000/EC contains inter alia provisions for the identification of priority substances (PS) to be addressed at EU level. These were initially identified by Directive 2008/105/EC, and the initial list was modified by Directive 2013/39/EU to include 45 substances. EU Member States are required to monitor PS, and identify water bodies at risk of exceeding environmental quality standards (EQS). In order to identify which PS may be tackled through appropriate river basin management measures, and dispose of a benchmark to evaluate progress on pollution control, EU Member States must also develop emission inventories of PS. However, 10 years after the entry into force of Directive 2008/105/EC, emission inventorying is still rather heterogeneous and far from complete across the EU, with few inventories officially adopted or publicly accessible. The only inventory available at European level is the European Pollutant Release and Transfer Register (E-PRTR) provided for by Regulation (EC) No 166/2006,^1^ covering major industrial installations. However, the E-PRTR does not include diffuse emissions except for metals, fluoranthene and anthracene. EU Member States report emissions to the Water Information System for Europe (WISE: https://water.europa.eu/), but the information currently available shows limited quality, completeness and homogeneity. The difficulties in producing emission inventories owe in part to the complexity of the factors controlling the release and environmental fate of chemicals: the use pattern and intensity of chemicals still in use, the environmental stockpiles of legacy chemicals, and the atmospheric long range transport of persistent multimedia substances are only poorly, if ever, known. Moreover, a chemical may undergo substantially different retention and elimination in wastewater treatment plants depending on their design and operation, or in soils or riverine ecosystems depending on their residence time, organic matter content and other landscape and climate parameters (Hollander et al., 2009).

In the estimation of emissions, it may be practical to distinguish point from diffuse source emissions. The former are associated to a single and well identified responsible, individually reported in dedicated registers, while the latter are by definition associated to an area without knowing their exact location therein.

In this paper we consider as point emissions only those in the E-PRTR, and we describe a first attempt at quantifying diffuse emissions of PS based on the information available at European scale, using as simple a model as possible and capitalizing on the available measurements of PS collected in a pan-European repository (IPCheM) as described in Section 2. We first use observed concentrations to back-calculate emission factors of several PS, and then use these emission factors to estimate PS loads and concentrations. After comparing our estimates of emissions with those reported by EU Member States whenever available, and our computed concentrations with independent observations, we discuss the strengths and limitations of the approach, and draw recommendations towards advancing large scale chemical emission inventories.

## 2 Materials and methods

Although measured concentrations should be in principle related to emissions of a chemical substance in the upstream catchment, estimating diffuse emissions from concentrations requires several assumptions which may seldom hold strictly true. In this work, we start from the simplest possible assumption: emissions are (1) constant in time, and (2) homogeneous across the EU, being only proportional to the intensity of human activities, described by the simple proxies of population (for household and industrial chemicals) and agricultural land (for agrochemicals). We refer to the spatial distribution of population or agricultural land as “emission patterns”.

The assumption of emissions constant in time may be apparently inappropriate for chemicals with clear occasional use (e.g. pesticides), and also emissions from relatively steady sources always show fluctuations in time.

The assumption of emission homogeneity across the EU ignores the impact of different agricultural practices, lifestyles and economic activities on the use of chemicals that can be found across the continent, besides the specificities of landscape, climate and infrastructure.

Although the impact of the errors introduced with these assumptions is difficult to quantify, we may anticipate that the estimates they underpin will be necessarily affected by large uncertainties.

However, although apparently simplistic, these assumptions may not be more questionable than more sophisticated alternatives in the absence of conclusive evidence on the spatiotemporal distribution of emissions hence, following the Ockham's razor principle, they do not appear to be an unreasonable starting point to help better understanding what we actually know of emissions of PS in Europe, and how we could bridge knowledge gaps towards pan-European emission inventories.

### 2.1 Back-calculation of emission factors

Following the approach extensively presented in Pistocchi and Marinov, 2014a, for the generic J^th^ chemical, we assume the diffuse emission intensity at any point (*ξ*, *η*) in space to be:

(1) $$E_{J}\left(\xi ,\eta \right)=\varepsilon_{J}\mathrm{EP}\left(\xi ,\eta \right)$$

where EP(*ξ*, *η*) is the value of an assumed and known emission pattern at the generic point (*ξ*, *η*),and ε_*J*_ the chemical's emission factor. Moreover, we assume emissions to be stationary in time, and the travel time of water across a river basin to be constant and known. The load of the chemical at a river cross section (x,y) is then (Pistocchi and Marinov, 2014b, pp. 413–415):

(2) $$L_{J}\left(x,y\right)=\int_{A\left(x,y\right)}\varepsilon_{J}\mathrm{EP}\left(\xi ,\eta \right)e^{-\frac{\ln2}{\mathrm{DT}50_{J}}t\left(\xi ,\eta \right)}\;\mathrm{d\xi}\;\mathrm{d\eta}$$

where A(x,y) is the drainage area of river section (x,y), *t*(*ξ*, *η*) is the water time of travel from point (*ξ*, *η*) to river section (x,y) and DT50_J_ is the overall dissipation half-life of the chemical. DT50_J_ and ε_J_ are the two model parameters that must be calibrated once *EP*(*ξ*, *η*) and *t*(*ξ*, *η*) are given. Let us now consider a set of observed loads of the chemical at a number of cross sections of a river network, where the influence of point source emissions can be neglected. If the abovementioned assumptions are acceptable, observed loads should be reasonably correlated to the load proxy defined as (Pistocchi and Marinov, 2014a; Pistocchi et al., 2012):

(3) $$\Phi \left(x,y\right)=\frac{L_{J}\left(x,y\right)}{\varepsilon_{J}}=\int_{A\left(x,y\right)}\mathrm{EP}\left(\xi ,\eta \right)e^{-\frac{\ln2}{\mathrm{DT}50}t\left(\xi ,\eta \right)}\;\mathrm{d\xi}\;\mathrm{d\eta}$$

If we compute the load proxy Φ(x, y) for different emission patterns and different values of DT50, we can compare each case with the observed loads. An ideal load proxy would explain 100% of the variance of observed loads, and the corresponding best fit linear model would have a zero intercept, meaning no load is observed when the proxy is null. In this case, the slope of the best fit linear model would be an appropriate estimate of the emission factor, and the corresponding DT50 would be expected to represent the overall dissipation half-life of the chemical.

For a pan-European assessment, we first estimate the water travel time on the basis of the European CCM2 synthetic stream network derived from the SRTM digital elevation model at a resolution of 100 m (Vogt et al., 2007), corresponding to sub-basins with an average size of about 7 km^2^. We use the hydraulic geometry equations proposed by Pistocchi and Pennington, 2006, for the European stream network, taking as annual average discharge the average for the period 2005–2013 simulated with the LISFLOOD model (Burek et al., 2013) calibrated for Europe (Bisselink et al., 2018). The travel time through lakes and reservoirs is taken from the HydroLakes dataset (Messager et al., 2016).

As an emission pattern we used human population and agricultural area. Population was derived from the HYDE database (Klein Goldewijk and Van Drecht, 2006) for the year 2000, while agricultural land was derived from the Corine Land Cover 2012 dataset (https://land.copernicus.eu/pan-european/corine-land-cover/clc-2012). For comparison, we also considered emission patterns of human population connected to a wastewater treatment plant, and livestock density, as estimated by Bouraoui et al., 2009. For each emission pattern, we computed a load proxy for DT50 values of 1, 3, 5, 7, 10, 20, 50, 100 and 1000 days. This corresponds to a total of 36 load proxies, one for each combination of one of the four emission patterns and one of the nine values of DT50. Load proxies have the same units of measurement as the corresponding emission pattern (e.g. persons, km^2^ of agricultural land, etc.). These load proxies could be then compared with observed loads to estimate emission factors in units of load per person, km^2^, etc.

Measuring loads requires simultaneous measurement of water flow Q and concentration C at the same river cross section, but this type of information is not systematically available for the EU. Monitored concentration data are however available in the European Commission's IPCheM platform (https://ipchem.jrc.ec.europa.eu). IPCheM aims at collecting virtually all monitoring data publicly available in Europe, in all environmental media, by linking to existing data repositories. In particular, it receives data from the Water Information System for Europe (WISE: https://water.europa.eu/freshwater) where, inter alia, monitoring data on PS produced by the EU Member States are collected. We initially queried the IPCheM database for all 45 priority substances listed in Annex I of Dir. 2013/39/EU, finding data covering a sampling period from 2000 to 2008, totalling 367,114 records and 1995 sampling stations in 23 EU countries (excluding Cyprus, Croatia, Luxembourg, Malta and Sweden), all with geographic coordinates available. The following PS were excluded from this work due to the number of available and quantifiable observations being too small: Aclonifen, Brominated diphenylethers (PBDE), Cybutryne, Octylphenols and their ethoxylates, Dioxins and Dioxin-like compounds, PFOS, Hexabromocyclododecanes (HBCDD), Polycyclic Aromatic hydrocarbons (PAHs, excluding Anthracene, Fluoranthene and Naphthalene) and Trichlorobenzenes. All in all, we consider 36 out of the 45 PS as listed in Table 1. Chemical loads were estimated from concentrations and river water flow at all IPCheM sampling points. First of all, for each river segment in the CCM2 stream network, we estimated the daily water discharge for the date of each sample from a time series of 5-km gridded simulated discharges obtained from LISFLOOD. Then, IPCheM sampling points were also associated to the nearest CCM2 river segment. The product of measured concentration and simulated water flow finally yielded one estimate of load at each point and for each sampling date.

**Table 1:** Priority substances addressed in this study (AA=EQS for annual average concentrations; MAC=EQS for maximum admissible concentrations).

CAS number	Name of priority substance	Main applications	AA ug/L	MAC ug/L	Note
107–06-2	1,2-dichloroethane	Industrial solvent, very volatile, related to urban/industrial uses		10	
15,972–60-8	Alachlor	Herbicide, banned in 2006 (Commission Decision 2006/966/EC)	0.3	0.7	
120–12-7	Anthracene	Mainly a by-product related to urban/industrial uses	0.1	0.1	MAC 0.4 in 2013
1912-24-9	Atrazine	Herbicide, banned in 2004 (Commission Decision 2004/248/EC)	0.6	2	
71–43-2	Benzene^a^	Industrial solvent, very volatile. Restricted use^a^	10	50	
42,576–02-3	Bifenox	Herbicide	0.012	0.04	Added in 2013
7440-43-9	Cadmium and its compounds	Broadly related to urban/industrial uses as well as agricultural fertilizers	0.08 to 0.25	0.45 to 1.5	
470–90-6	Chlorfenvinphos	Insecticide banned in 2002 (Regulation (EC) No 2076/2002)	0.1	0.3	
85,535–84-8	Chloroalkanes, C10–13	Complex mix of different compounds used in industrial applications and related to urban/industrial uses	0.4	1.4	
67–66-3	Trichloromethane (chloroform)^b^	Industrial solvent, very volatile, related to urban/industrial uses. Restricted use^b^	2.5		
2921-88-2	Chlorpyrifos (Chlorpyrifos-ethyl)	Insecticide	0.03	0.1	
52,315–07-8	Cypermethrin	Insecticide	0.00008	0.0006	
117–81-7	Di(2-ethylhexyl)phthalate (DEHP)	Plasticizer, related to urban/industrial uses	1.3		
75–09-2	Dichloromethane	Industrial solvent, very volatile, related to urban/industrial uses	20		
62–73-7	Dichlorvos	Insecticide banned in 2007 (Commission Decision 2007/387/EC)	0.0006	0.0007	Added in 2013
115–32-2	Dicofol	Insecticide banned in 2008 (Commission Decision 2008/764/EC)	0.0013		Added in 2013
330–54-1	Diuron	Herbicide, also extensively used in urban green areas, infrastructure, etc.	0.2	1.8	
115–29-7	Endosulfan	Insecticide banned in 2005 (Commission Decision 2005/864/EC)	0.005	0.01	
206–44-0	Fluoranthene	Mainly a by-product related to urban/industrial uses	0.1	1	AA 0.006, MAC 0.12 in 2013
76–44-8/ 1024-57-3	Heptachlor and its epoxide	Insecticide banned in 2004 (Regulation (EC) No 850/2004)	0.0000002	0.00003	Added in 2013
118–74-1	Hexachlorobenzene	Insecticide and by-product of chloro-alkali industry banned in 2004 (Regulation (EC) No 850/2004)	0.01	0.05	AA replaced by Biota in 2013
87–68-3	Hexachlorobutadiene	By-product of chloro-alkali industry	0.1	0.6	AA replaced by Biota in 2013
608–73-1	Hexachlorocyclohexane	Insecticide banned in 2004 (Regulation (EC) No 850/2004)	0.02	0.04	
34,123–59-6	Isoproturon	Herbicide, also extensively used in urban green areas, infrastructure, etc. banned in 2016 (Regulation (EU) 2016/872)	0.3	1	
7439-92-1	Lead and its compounds	Broadly related to urban/industrial uses	7.2	14	AA 1.2 in 2013; MAC added in 2013
7439-97-6	Mercury and its compounds	Broadly related to urban/industrial uses. Plant protection products banned in 1979 (Directive 79/117/EEC). Biocidal products banned in 2007 (Commission Regulation (EC) No 1451/2007).	0.05	0.07	AA replaced by Biota in 2013
91–20-3	Naphthalene	Industrial use	2.4	130	AA 2 in 2013; MAC added in 2013
7440-02-0	Nickel and its compounds	Broadly related to urban/industrial uses	20	34	AA 4 in 2013; MAC added in 2013
–	Nonylphenols and Nonylphenolethoxylates	Detergents and their degradation products. Restricted use.^c^ Constituent of formulations of plant protection products, banned in 2002 (Regulation (EC) No 2076/2002).	0.3	2	
608–93-5	Pentachlorobenzene	By-product of chloro-alkali industry	0.007		
87–86-5	Pentachlorophenol	Insecticide/disinfectant. Banned as plant protection product in 2002 (Regulation (EC) No 2076/2002).	0.4	1	
124,495–18-7	Quinoxyfen	Fungicide	0.15	2.7	Added in 2013
122–34-9	Simazine	Herbicide banned in 2004 (Commission Decision 2004/247/EC)	1	4	
886–50-0	Terbutryn	Herbicide banned in 2002 (Regulation (EC) No 2076/2002)	0.065	0.34	Added in 2013
–	Tributyltin compounds	Plant protection product, banned in 2002 (Regulation (EC) No 2076/2002). Biocidal agent (anti-fouling) banned in 1998 (Directive 98/8/EC). Treatment of industrial waters, banned in 2006 (Regulation (EC) No 1907/2006).	0.0002	0.0015	
1582-09-8	Trifluralin	Herbicide, banned in 2010 (Commission Decision 2010/355/EU)	0.03		

a Benzene is not permitted in toys or parts of toys as placed on the market where the concentration of benzene in the free state is in excess of 5 mg/kg of the weight of the toy or part of toy. It shall not be used in concentrations equal to, or greater than, 0,1% by mass in substances or preparations placed on the market. 3. However, paragraph 2 shall not apply to: (a) motor fuels which are covered by Directive 98/70/EC; (b) substances and preparations for use in industrial processes not allowing for the emission of benzene in quantities in excess of those laid down in existing legislation; (c) waste covered by Council Directive 91/689/EEC of 12 December 1991 on hazardous waste (1) and Directive 2006/12/EC.

b Chloroform shall not be used in concentrations equal to or >0,1% by weight in substances and preparations placed on the market for sale to the general public and/or in diffusive applications such as in surface cleaning and cleaning of fabrics. Preparations containing them in concentrations equal to or >0,1% shall be legible and indelibly marked as follows: ‘For use in industrial installations only’. By way of derogation this provision shall not apply to: (a) medicinal or veterinary products as defined by Directive 2001/82/EC and Directive 2001/83/EC; (b) cosmetic products as defined by Directive 76/768/EEC.

c Nonylphenol ethoxylates shall not be placed on the market, or used, as substances or in mixtures, in concentrations equal to or >0,1% by weight for the following purposes: (a)industrial and institutional cleaning except: controlled closed dry cleaning systems where the washing liquid is recycled or incinerated, and cleaning systems with special treatment where the washing liquid is recycled or incinerated. (b) domestic cleaning; (c) textiles and leather processing except: processing with no release into waste water, and systems with special treatment where the process water is pre-treated to remove the organic fraction completely prior to biological waste water treatment (degreasing of sheepskin); (d) emulsifier in agricultural teat dips; (e) metal working except uses in controlled closed systems where the washing liquid is recycled or incinerated; (f) manufacturing of pulp and paper; (g) cosmetic products; (h) other personal care products except spermicides; (i) co-formulants in pesticides and biocides. However, national authorizations for pesticides or biocidal products containing nonylphenol ethoxylates as co-formulant, granted before 17 July 2003, shall not be affected by this restriction until their date of expiry.

In principle, load estimates for different dates at a given location should be averaged into one estimate. However, due to the spatial and temporal heterogeneity of the IPCheM data and the different abundance of samples for different substances, it was decided to consider all estimates of loads as part of a single statistical population in further analyses. The product of observed concentration of a chemical and river water discharge estimated as described above is referred to hereinafter as “observed load”, implying the uncertainty on water discharge to be unimportant for the objectives of this study. A supporting information document (SI) provides additional elements to appreciate the impact of hydrological uncertainty on the results.

It must be stressed that, in this exercise, we refer exclusively to the dissolved phase concentration of contaminants and, consequently, emissions and concentrations that we model are those in dissolved phase. Modelling concentrations in particulate phase would require knowing the concentration of suspended solids at all samples, which is not the case. For substances primarily in dissolved phase, this does not represent a significant limitation. Substances that tend to partition to solids generally undergo much more complex environmental fate and transport. For these, modelling the dissolved phase can be seen as an initial step to be complemented by further analysis.

We computed a best-fit linear model of a set of observed loads using the weighted least squares (WLS) method (e.g. Strutz, 2016) using one load proxy at a time as the explanatory variable. Weights were computed as the square root of the “observed” load. The best-fit linear model uncertainty was estimated through a bootstrap resampling procedure iterated 1000 times for each substance. We identify the load proxy best representing observed loads using the criterion of minimizing, in the intercept/explained variance (R^2^) space, the normalized Euclidean distance from the ideal point (R^2^ = 100%, intercept = 0). The slope of the linear model corresponding to the load proxy closest to the ideal point identified for each chemical is taken as an estimate of the emission factor, while the intercept is ignored.

In the above procedure, we implicitly assume that observed loads reflect only diffuse emissions, i.e. those associated to a given emission pattern, while in reality point emissions may be locally relevant. The E-PRTR inventory contains data on point emissions from industrial installations for 29 of the 36 chemicals studied here (excluding 7 chemicals, namely Bifenox, Cypermethrin, Dichlorvos, Dicofol, Hexachlorocyclohexane, Quinoxyfen and Terbutryn). These emissions may be used to compute the corresponding load for each chemical as:

(4) $$L_{p,J}\left(x,y\right)=\int_{A\left(x,y\right)}P_{J}\left(\xi ,\eta \right)e^{-\frac{\ln2}{\mathrm{DT}50_{J}}t\left(\xi ,\eta \right)}\;\mathrm{d\xi}\;\mathrm{d\eta}$$

where P_*J*_(*ξ*, *η*) is the emission of the J-th chemical from point sources at point (*ξ*, *η*) reported in *E*-PRTR.

If the observed loads used to estimate the emission factors are significantly affected by these point source emissions, we may expect that the estimates of the diffuse emission factors can be consequently distorted. In order to appreciate the impact of the E-PRTR point sources, we compute the ratios of point loads (Eq. 4) to diffuse loads (Eq. 2) for all chemicals, and we use as indicators of impact the maximum of point source contribution to total loads among all substances (Z), and the number of substances with a contribution to the total higher than 25% (W), i.e.:

(5) $$\begin{array}{c} Z\left(x,y\right)=\max_{J\in \left\{1,\ldots ,N\right\}}\left(\frac{L_{p,J}\left(x,y\right)}{L_{J}\left(x,y\right)}\right)\\ W\left(x,y\right)=\sum_{J\in \left\{1,\ldots ,N\right\}}B\left(\frac{L_{p,J}\left(x,y\right)}{L_{J}\left(x,y\right)}>0.25\right) \end{array}$$

where N is the number of chemicals, and function B(−) is 0 if the argument is false, and 1 otherwise. High values of indicators Z and W at the sites of observed concentrations would suggest the need to correct observations for the effect of point source emissions.

### 2.2 Verification of the emission inventories

The best load proxy selected for each chemical defines, in principle, its diffuse emission pattern, the corresponding emission factor and DT50, and enables computing diffuse emissions (Eq. 1) as well as chemical concentrations at each point in the stream network:

(6) $$C_{J}\left(x,y\right)=\frac{L_{J}\left(x,y\right)}{Q\left(x,y\right)}$$

where Q(x,y) is river water flow. Assuming loads to be stationary in time, concentrations change in time with water flow, typically showing seasonal as well as event-to-event variability. In order to obtain a representative value of concentration, we limit our analysis here to concentrations obtained using annual average water flow for Q(x,y). Annual average flow is estimated with the popular Budyko equation (Budyko, 1974), which shows being always very close to the average of the LISFLOOD daily simulated discharges used to derive “observed loads” (see SI for additional details). Concentrations computed in this way can be then compared with independently measured concentrations. As our estimates assume uniform emission factors across Europe, a comparison cannot be made with observations pointwise, but only in terms of frequency distributions.

For the sake of model verification, we use more recent data added to IPCheM during the development of the work, relating to a sampling period from 2009 to 2014, totalling 1,976,035 records and 6532 sampling stations in 25 EU countries (excluding Spain, Hungary, Romania) of which 3147 with geographic coordinates available. This dataset has a relatively small number of stations in common with the dataset used for the estimation of emission factors, and no overlap in time (see additional details in the SI). Hence it can be safely regarded as independent.

The maps of estimated concentrations can be also compared with the spatial distribution of the cases of exceedance of EQS for each chemical, as reported by EU Member States in the second round of their River Basin Management Plans (EEA, 2018). We draw a comparison following the “prediction rate” approach (Chung and Fabbri, 2003): first we sort all sub-basins in CCM2 by decreasing estimated concentration, and then we compute the cumulative percentage of reported exceedances of EQS for each PS, along the sorted list of sub-basins. The plot of the cumulative number of exceedances as a function of the frequency of exceedance of the corresponding concentration (the “prediction rate”) should lay above the 45° (1:1) line if concentration is a better predictor of exceedances than a random extraction, and the better concentration is a predictor, the closer the curve to the y axis. This plot can be regarded as a “receiver-operator characteristic” (ROC) curve, or plot of true positive rate versus false positive rate (Swets, 1988; Fawcett, 2006). ROC curves can be also built with the same logics for *observed* concentrations, limiting the calculation of cumulates to exceedances reported only in sub-basins with an observed value of concentration. Consequently, in this case the number of reported exceedances that are considered is typically much smaller and may be zero (i.e. there are no observations in any sub-basin with reported exceedance), which results in no curve to be possibly drawn, and generally in less smooth ROC curves.

## 3 Results and discussion

### 3.1 Emissions and loads

Table 2 summarizes the emission factor estimated for each chemical. The DT50 corresponding to the best performing linear model in the R^2^/intercept space, for the assumed emission pattern, is indicated in the table as “modelled DT50”. In the SI, we report the plots of explained variance and intercept of the various load proxies for each of the 36 substances considered here. Moreover, for each chemical we report the emission factors for the different values of DT50 assuming agricultural land and population as emission patterns.

**Table 2:** Inverse modelling results for the selected priority substances. The explained variance is: high if R^2^ ≥ 0.6; medium if R^2^ > 0.4; low if R^2^ ≤ 0.4. Emission patterns (EP) are agricultural land (A), population (P), livestock (L) or collected population (C). An uniform (U) pattern denotes catchment area alone. The "best EP" is the EP with highest R^2^ and lowest intercept (more than one "best EP" for a chemical means more EP have similar performance)

CAS number	Name of priority substance	R^2^	Best EP	Assumed EP	Modelled DT50 (days)	Reported DT50 (days)	Selected DT50 (days)	EF Pop. (ng/ind/s)	EF Agri. (ng/km2/s)
107–06-2	1,2-dichloroethane	Low	A, U, P	P	10	Hours to days^a^	10	16.8	–
15,972–60-8	Alachlor	High	A, L	A	20	18–37^b^	20	–	203.7
120–12-7	Anthracene	Medium	A, U, P	P	7	<1^b^	7	0.3	–
1912-24-9	Atrazine	High	U	A	1000	50–100^b^	1000	–	325.1
71–43-2	Benzene	Low	P	P	1000	About 10^b^	10	13.95	–
42,576–02-3	Bifenox	High	U	A	1000	0.11^c^	3	–	598.08
7440-43-9	Cadmium and its compounds	Low	A, U	A	1000	–	1000	–	4897.06
470–90-6	Chlorfenvinphos	Low	A	A	100	50^b^	100	–	78.2
85,535–84-8	Chloroalkanes, C10–13	High	A, L, U	P	1000	1000^b^	1000	218.7	–
67–66-3	Trichloromethane (chloroform)	Low	A, U, P	P	10	1–5^b^	10	9.3	–
2921-88-2	Chlorpyrifos (Chlorpyrifos-ethyl)	Low	A	A	10	5^b^	10	–	118.1
52,315–07-8	Cypermethrin	Medium	L, U	A	1000	50–100^f^	1000	–	128.6
117–81-7	Di(2-ethylhexyl)phthalate (DEHP)	Low	P, U	P	1000	100–500^b^	1000	31.4	–
75–09-2	Dichloromethane	Low	P	P	10	1–5^b^	10	38.9	–
62–73-7	Dichlorvos	Medium	A, U	A	1000	<1^g^	3	–	335.52
115–32-2	Dicofol	High	P, C, U	A	1000	10–50^b^	10	–	181.41
330–54-1	Diuron	High	P, U, A	A	20	100^b^	20	–	549.1
115–29-7	Endosulfan	Low	P, A	A	10	10^b^	10	–	100.8
206–44-0	Fluoranthene	Medium	P	P	10	10^b^	10	1.2	–
76–44-8/ 1024-57-3	Heptachlor and its epoxide	Low	L, U, A	A	3	1000^b^	1000	–	21.47
118–74-1	Hexachlorobenzene	Low	A, U	A	1000	1000^b^	1000	–	33.56
87–68-3	Hexachlorobutadiene	Low	A, U, P	P	1000	100–500^b^	1000	1.1	–
608–73-1	Hexachlorocyclohexane	High	A	A	20	–	20	–	70.2
34,123–59-6	Isoproturon	Medium	P, U	A	1000	100–500^b^	1000	–	418.3
7439-92-1	Lead and its compounds	High	P,U	P	1000	–	1000	142.0	–
7439-97-6	Mercury and its compounds	High	P, U	P	100	–	100	4.9	–
91–20-3	Naphthalene	Medium	P	P	5	7–150^h^	5	2.7	–
7440-02-0	Nickel and its compounds	High	P, U	P	100	–	100	127.6	–
–	Nonylphenols	Low	A, L, U, P	P	1000	100–500	1000	24.1	–
608–93-5	Pentachlorobenzene	Low	A, U, P	P	1000	1000^b^	1000	0.4	–
87–86-5	Pentachlorophenol	Low	P, A	A	3	5^b^	3	–	299.0
124,495–18-7	Quinoxyfen	High	L	A	1000	127^e^	1000	–	230.0
122–34-9	Simazine	Low	P, U, A	A	7	50–100^b^	100	–	181.56
886–50-0	Terbutryn	Medium	L, U	A	1000	100–500^b^	1000	–	186.8
–	Tributyltin compounds	High	A, U	P	20	–	20	0.2	–
1582-09-8	Trifluralin	Medium	U	A	10	100–500^b^; <1^d^	10	–	87.8

a http://www.eurochlor.org/media/49227/8-11-4-1_marine_ra_edc.pdf*.*

b Compilation of Vink and van der Zee, 1997, Norwegian Pollution Control Authority, 2005, Aronson et al., 2006, and Environment Canada, 1993.

c https://circabc.europa.eu/webdav/CircaBC/env/wfd/Library/framework_directive/thematic_documents/priority_substances/supporting_substances/substance:impacts/Bifenox.pdf*.*

d https://www.ospar.org/documents?d=6983*.*

e https://sitem.herts.ac.uk/aeru/ppdb/en/Reports/580.htm*.*

f https://www.cdpr.ca.gov/docs/emon/pubs/fatememo/cyperm.pdf*.*

g https://sitem.herts.ac.uk/aeru/ppdb/en/Reports/220.htm

h https://echa.europa.eu/documents/10162/5f0beb6c-575f-4a1b-aff5-b37f06eb3852*.*

Table 2 shows the best-performing emission patterns for each chemical, as well as an indication of the corresponding explained variances (R^2^).

In some cases, the assumed emission pattern does not yield the best performing proxy, and one or more of the other emission patterns considered (besides agriculture and population, collected population and livestock) may perform better; moreover, catchment area is the best descriptor in 10 cases out of 36, suggesting a spatially uniform emission. This may be due to a higher complexity of emission sources, including atmospheric long-range transport especially for the most persistent chemicals, to some extent overriding emissions from population or agriculture in the upstream catchment area.

The “modelled DT50” were also compared with a range of DT50 reported in the literature (see Table 2) in order to check that they retained a physical meaning. Reported DT50 values are inherently uncertain, because they stem from experiments, or from the calibration of models, which may be difficult to generalize; in any case they should be regarded merely as broad indications. For this reason, we expect our values of DT50 to match the range of reported values only by order of magnitude. In the case of Cadmium and Hexachlorobenzene, the assumption of an emission pattern is problematic, because a priori they may be equally associated to population and agriculture. Due to the higher explained variance of observed loads by the latter with respect to the former, we have assumed these chemicals to follow agriculture. However, particularly in the case of Hexachlorobenzene, the difference in explained variance is very small.

For six out of the 36 chemicals (Benzene, Bifenox, Dichlorvos, Dicofol, Heptachlor and Simazine) the DT50 identified by the statistical optimization procedure was excessively distant from reported values. Other substances feature “modelled DT50” which are borderline with respect to the reported range, and must be regarded with attention. It should be noted that the values of DT50 indicated in Table 2 represent the optimal values in a statistical sense, but often retain little physical meaning and should not be interpreted as an estimate of a “real” half-life. Indeed, when the DT50 is unrealistically low or high, often a model with a more realistic DT50 yields a model performance only slightly inferior to the optimal “modelled DT50”, fully justifying the selection of a DT50 different from the modelled one on the basis of expert judgment.

Finally, Table 2 displays the emission factor computed with the assumed emission pattern and the selected DT50. This is in general the “modelled DT50”, except for the 6 substances mentioned above. In these cases, a DT50 deemed more realistic was assigned ad hoc, after checking that the variance explained by the load proxies did not deteriorate significantly. Although other emission patterns may perform better in some cases, we only use population or agricultural land as initially assumed (based on the expected use of each chemical in agriculture, or in other human activities), because of their simplicity, robustness and ease of interpretation; this is acceptable considering that their performance is always reasonably close to that of the best performing emission patterns in each case.

Emission patterns (agriculture or population), emission factors and DT50 values in Table 2 enable computing diffuse emissions and corresponding loads. These can be compared with point source emissions and loads using Eq. 4 (see Table 3). In the SI, we show that indicators Z and W (Eq. 5) are usually low, particularly at the sites of observed loads. Consequently, the diffuse emission factors estimated above are expected not to be distorted by the influence of point sources. Therefore, although the contribution of point source emissions may be very significant locally, the frequency distribution of overall concentrations estimated across the EU is expected to be well approximated by the frequency distribution of concentrations stemming from diffuse sources only.

**Table 3:** Point source emissions of the 29 E-PRTR substances, as a fraction of diffuse emissions (statistics across all EU RBDs). Values for individual RBDs are mapped in the Supporting Information.

Substance	5%ile	Median	95%ile	RBDs with contribution > 0 (out of 202 RBDs)
1_2_dichloroethane	0.6%	5.2%	26.0%	42
Alachlor	0.2%	3.2%	17.8%	8
Anthracene	1.2%	15.8%	99.4%	33
Atrazine	0.3%	3.5%	88.7%	24
Benzene	3.8%	25.5%	94.6%	28
Cadmium	0.7%	7.0%	46.6%	107
Chlorfenvinphos	1.4%	4.6%	52.9%	7
Chloroalkanes_C10_13	0.0%	0.1%	0.5%	26
Chloroform	2.6%	13.2%	63.5%	65
Chlorpyrifos	1.1%	8.5%	98.6%	9
Di_2_ethylhexyl_phthalate	0.2%	4.7%	29.6%	88
Dichloromethane	0.4%	2.5%	28.4%	52
Diuron	0.4%	4.1%	93.8%	55
Endosulfan	0.8%	6.1%	44.4%	7
Fluoranthene	0.5%	4.9%	98.8%	52
Heptachlor	2.3%	29.5%	44.2%	3
Hexachlorobenzene	1.2%	14.4%	48.8%	12
Hexachlorobutadiene	0.1%	3.8%	35.5%	20
Isoproturon	0.3%	2.4%	86.2%	24
Lead	0.9%	7.2%	39.9%	117
Mercury	1.4%	6.9%	50.2%	110
Naphtalene	2.4%	35.2%	91.3%	39
Nickel	2.3%	15.4%	48.7%	126
Nonylphenol	0.1%	1.8%	58.5%	82
Pentachlorobenzene	3.5%	26.4%	57.0%	8
Pentachlorophenol	0.6%	6.8%	77.9%	37
Simazine	0.2%	2.6%	99.1%	25
Tributyltin	0.9%	11.4%	97.3%	20
Trifluralin	1.5%	8.2%	33.4%	4

**Fig. 1:**
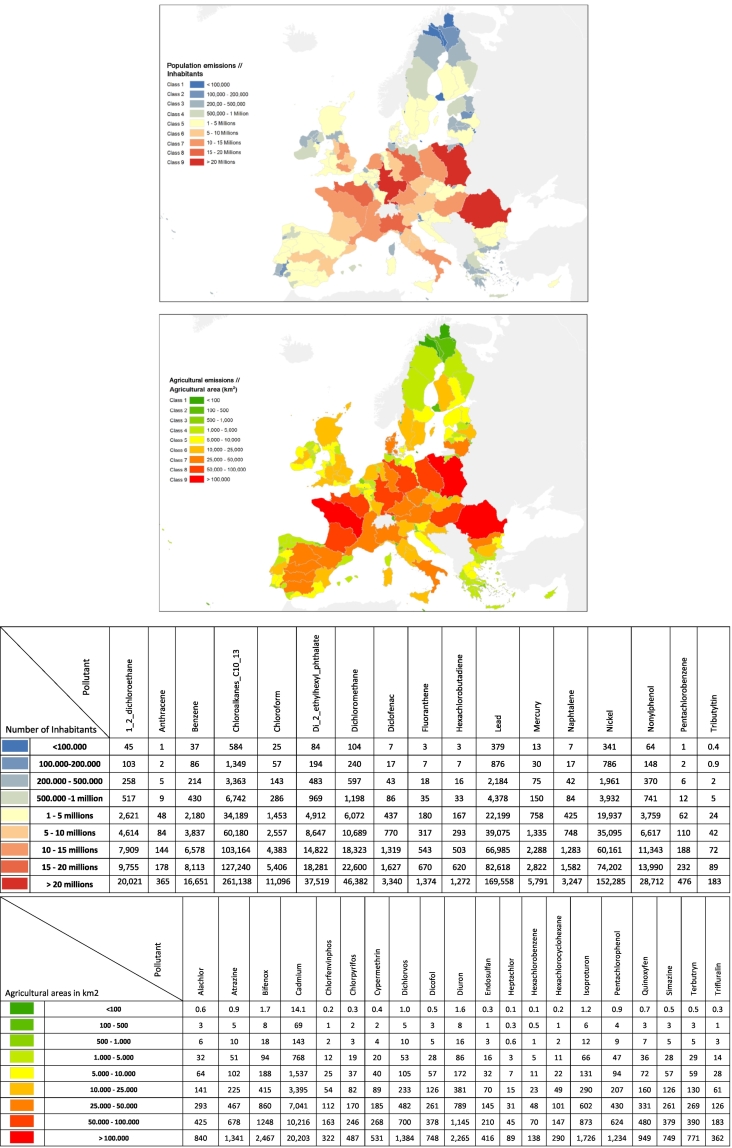
Diffuse emission maps (above) following population; (below) following agriculture. Emissions of chemicals are proportional to emission patterns according to the tables under the maps – values of emissions per RBD for each substance in kg/y.

Following the European guidance document on emission inventories (EC, 2012), we present emissions aggregated at river basin district sub-unit level. The sum of the emission pattern (population or agricultural land) within each mapping unit, times the corresponding emission factor yield diffuse emissions. Point sources, on the contrary, are specific to each substance. Fig. 1 shows diffuse emissions by river basin district (RBD), while point source emissions are provided in the SI as a percentage of total emissions by RBD, for the 29 substances covered in E-PRTR. Contributions from point sources may be very important in certain RBDs, but generally remain below one third of total emissions; moreover, when contributions are high they are generally concentrated in a limited number of RBDs across Europe (see Table 3).

**Fig. 2:**
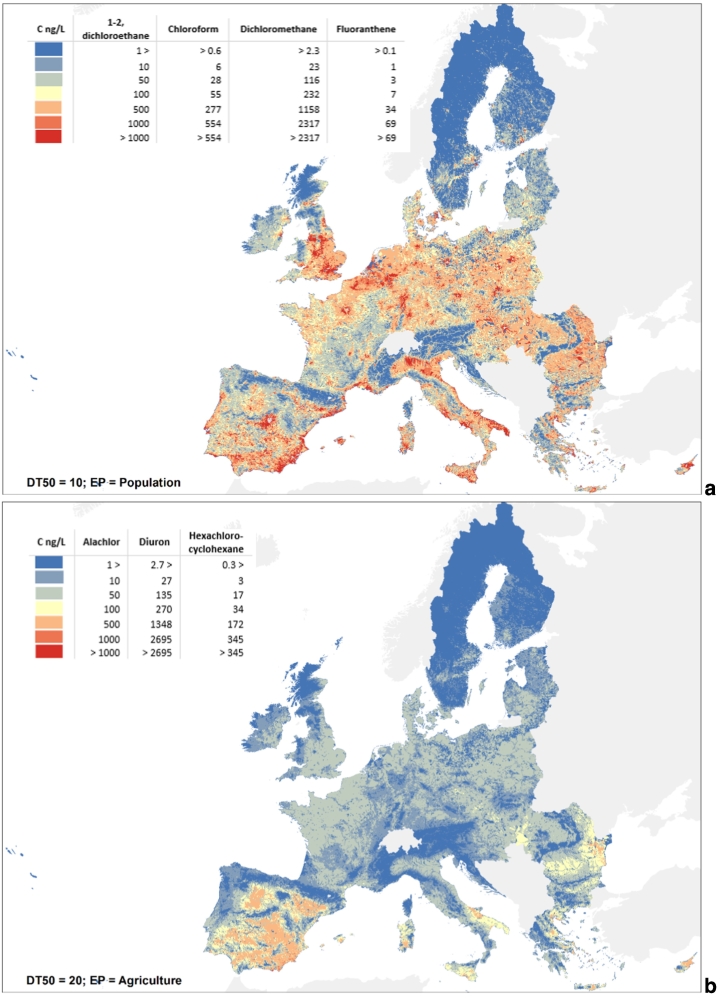
Example maps of concentration due to diffuse sources (a) from population; (b) from agriculture.

Loads and concentrations due to diffuse sources can be estimated with Eq. 2 and Eq. 6 on the basis of the emission patterns and dissipation half-lives, emission factors being uniform scaling constants across Europe. Therefore, substances being described by the same emission pattern and half-life have the same load and concentration distributions except for the scaling constant. Example maps of computed diffuse concentrations are shown in Fig. 2, representing two groups of substances having the same emission pattern and DT50. The maps for the remaining substances are provided in the SI. Contrary to diffuse emissions, point source emissions represented by E-PRTR facility discharges of chemicals generate loads and concentrations which differ from substance to substance and depend strictly on the location of emitting facilities. An overview of the spatial distribution of river stretches significantly affected by point sources is given by the indicator Z of Eq. 5 presented in the SI.

**Fig. 3:**
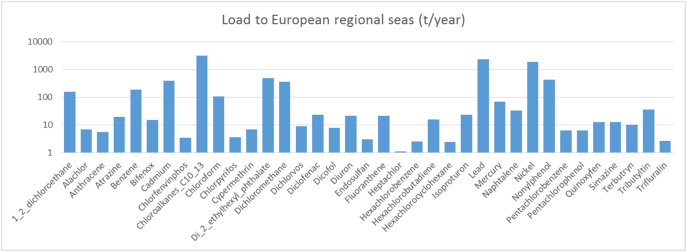
Loads to European seas from the 28 EU member states.

The calculation of loads is particularly informative when focusing on loads conveyed through the stream network to the European regional seas. Diffuse sources represented by emission factors and DT50 of Table 2 , combined with point sources from E-PRTR, yield for most substances loads in the range of a few tonnes per year, with only certain substances exceeding the levels of 100 tonnes per year (Fig. 3 and SI).

### 3.2 Model verification

**Fig. 4:**
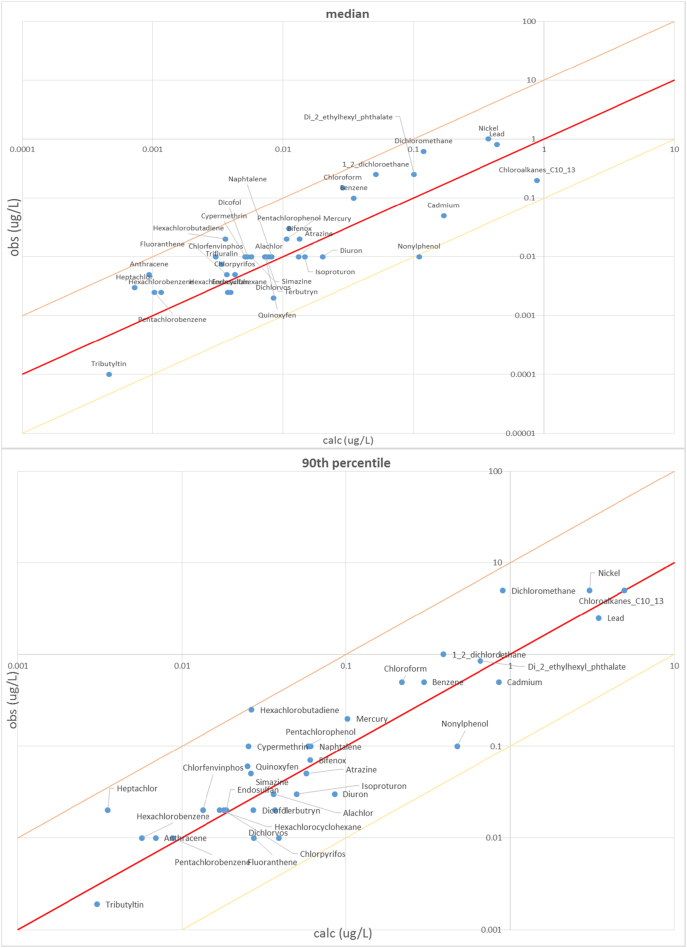

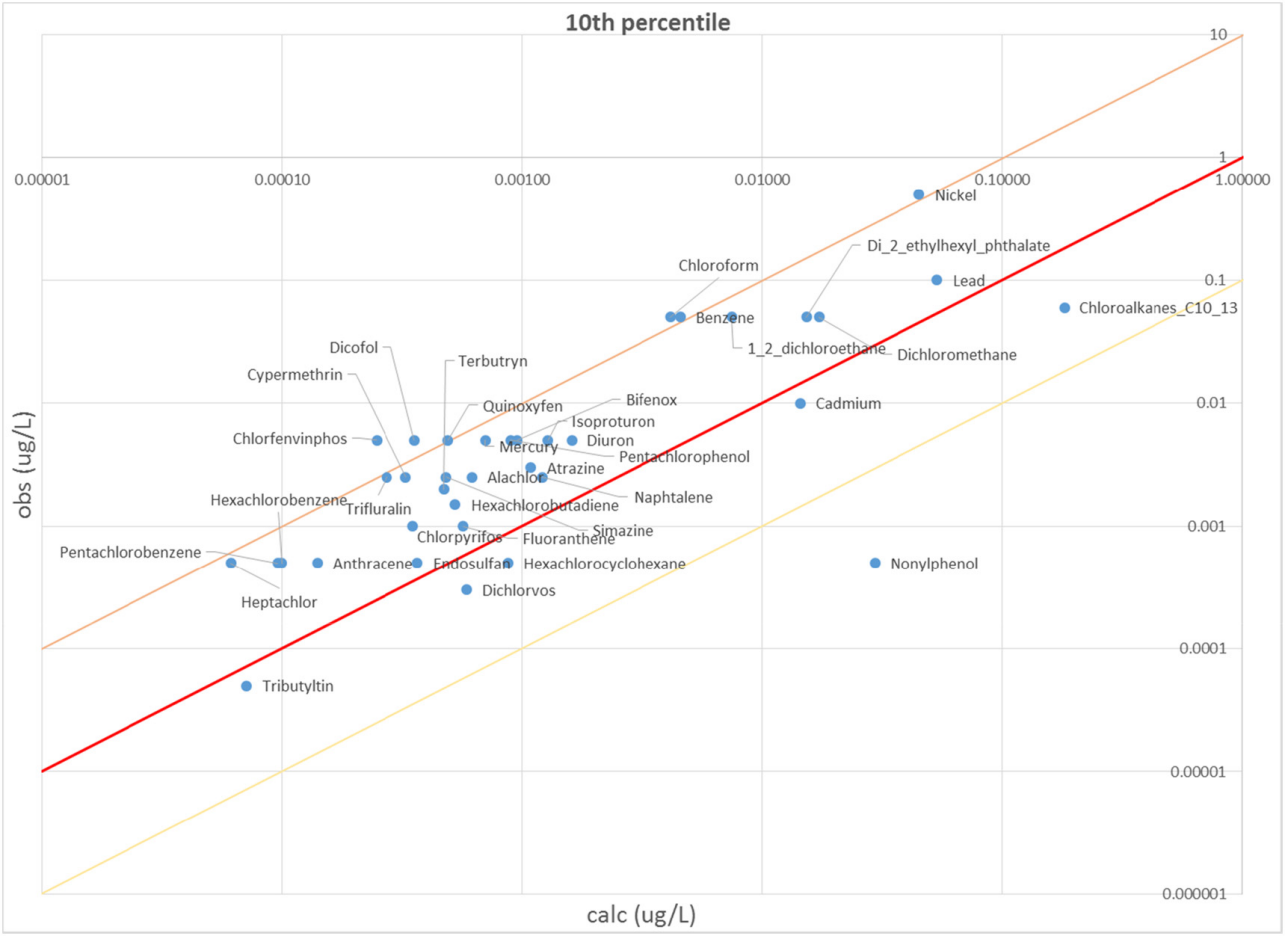
Scatter plot of EU concentrations medians for the 36 PS, considering only sub-basins with emission patterns and discharge values in the range of those in the sampling sites.

In order to verify the representativeness of computed concentrations with regard to the monitoring data used to estimate emission factors, we plotted the EU median, 10th and 90th percentiles computed on the population of observed (post-2009) and estimated concentrations for each of the 36 substances (Fig. 4). The percentiles of estimated concentrations are evaluated considering only the stream segments in the same range of drainage area as those with observations available. Overall, the model interprets observations reasonably well, with discrepancies within a factor of 10 except for the 10th percentile of a few substances. Unlike for medians and 90th percentiles, 10th percentiles also show a tendency to underestimation by the model.

Table 4 summarizes the prediction rate (percentage of reported EQS exceedances falling in the X % of rivers with highest concentration, with X = 10, 20, 50) of model-predicted concentrations for the 27 substances for which exceedances of EQS are reported in the EU. In the same table, prediction rates of observed concentrations from pre- and post-2009 monitoring sites are also reported. The full ROC curves for all substances, from which the prediction rates are estimated, are provided in the SI. Although, conceptually, the concentrations predicted by the model are annual averages and should be consistently compared with exceedances of annual average (AA) EQS, we conduct a comparison with exceedances of maximum admissible concentration (MAC) EQS as well. Most ROC curves appear to be clearly above the 1:1 line, hence show some capacity to predict reported exceedances. Chemicals with very weak model prediction both on AA and on MAC exceedances include Chlorfenvinphos, Diuron, Isoproturon, Pentachlorobenzene and Pentachlorophenol. Weak prediction is shown in the case of Chlorpyrifos, DEHP, and Hexachlorocyclohexane. Mercury is the only chemical for which, while the prediction of MAC exceedances is very weak, the prediction of AA exceedances is inverted (ROC curve below the 1:1 line), indicating that the model systematically predicts lower concentrations at sites with reported exceedances. For Cadmium and Hexachlorobenzene, the ROC curves obtained assuming population as an emission pattern (not shown here) would perform significantly better than those (shown in the SI) assuming emissions to follow agriculture. This further confirms that, for these two priority substances, the attribution to one of the two patterns is problematic.

**Table 4:**
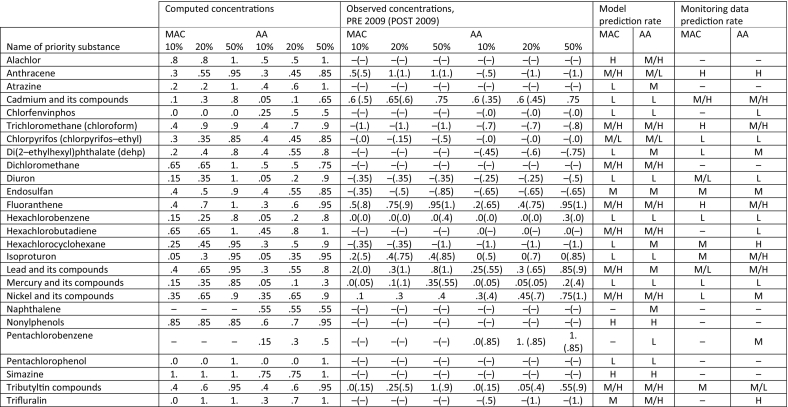
Prediction rates of reported EQS exceedances by modelled concentrations for 27 of the 36 substances, for which EQS exceedances are reported by Member States. For each priority substance, we show the share of total exceedances of both maximum acceptable concentration (MAC) and annual average (AA) EQS, falling within the 10%, 20% and 50% of highest concentration among EU rivers. For chemicals with IPChem samples available in the rivers with exceedances, we also show the share of exceedances corresponding to the highest 10%, 20% and 50% of measured concentrations. The table includes a qualitative judgment of the model prediction rate performance (H = high, L = low, M = medium, M/H, M/L is for intermediate performance).

All other substances show a ROC curve fairly above the 1:1 line for both AA and MAC exceedances, and in some cases (Alachlor, Nonylphenols and Simazine) even rather good predictions. The prediction performance does not seem to be related to the explained variance, nor to the identification of the emission pattern of the model. Comparison of Table 2 and Table 4 shows that relatively good predictions are obtained with relatively poor model's explained variance and vice versa, and irrespective of whether the assumed emission pattern is the best performing one. When inspecting the prediction rates based on monitoring data, both pre- and post-2009, it also appears that the prediction rate of observed concentrations with reported exceedances may be very weak or weak, comparably with modelled concentrations. This indicates that reported exceedances tend sometimes to occur at sites with relatively low observed concentrations, while there are less than expected reported exceedances at sites with higher observed concentrations, suggesting a need for an in-depth consistency check of reported exceedances and monitoring data.

## 4 Conclusions

We have used a set of monitoring stations from before year 2009 to identify emission patterns and dissipation half-lives, and to calibrate diffuse emission factors of WFD priority substances. Half-lives and emission factors were assumed to be uniform across Europe, corresponding to the case that use and discharge to the environment of these chemicals are similar in all EU countries. This enabled modelling a spatial distribution of concentrations, of which the frequency distribution is in agreement with similar, but independent monitoring data recorded from year 2009 onwards, within one order of magnitude: while the model's capacity to describe chemical pollution at a point is forcefully limited, it generally reconciles emissions with observed concentrations. Concentrations modelled assuming uniform emission factors and simple patterns (population and agriculture) also reasonably predict reported EQS exceedances for some chemicals. In other cases, predictions were on the contrary weak to very weak. However, also observed concentrations at monitoring sites highlighted very weak or weak predictions for certain substances. This suggests that the available monitoring data and reported EQS exceedances are not necessarily consistent. Indeed, reported exceedances refer to a period posterior to that of the monitoring, and arguably reflect a partly changing situation. Assuming uniform use and environmental discharge of priority substances across Europe may also be inappropriate. However, a comparison of predicted and observed concentrations in each EU Member State does not highlight clear patterns suggesting other drivers of emissions, such as climate or socioeconomic trends, to play a role across the EU (in this regard, the SI provides further details on the model errors found in different countries). Hence the variability of concentrations arguably owes more to the inherent variability of chemical emissions within any EU river basin than to apparent regional differences in chemical use.

Despite the limitations discussed above, the simple model of Eq. 2 and Eq. 4, with a uniform emission factor across Europe and assuming population or agriculture alone as diffuse emission pattern for a chemical substance, retains some usefulness for a first representation of emissions, concentrations and loads, and can be used to derive a consistent picture of chemical pollution, seeming to follow relatively simple drivers such as population and agriculture when examined at the European scale.

All in all, this exercise leads to a first pan-European inventory of emissions from both point and diffuse sources, enabling to appraise the relative importance of the two sources. The relatively minor contribution of point sources to observed concentrations, outside of hot spots near emissions, suggests that priority substances may come from widespread use (also back in the past, for phased-out but environmentally persistent chemicals, see Table 1). Therefore their management may be impossible at the river basin level alone, calling for a broader approach starting from the authorization phase of chemicals.

The emission inventories presented here were compared with existing emission inventories prepared by European Union Member States, as shown in the SI. The comparison highlighted a fair consistency of ours and the Member States' estimates, but also discrepancies whose reasons deserve a more in-depth and case-by-case discussion (see SI). All in all, our emission inventories must be regarded as a starting point. They must be improved using better monitoring data to unveil the variability and peculiarity of pollution sources, forcefully compressed and concealed under the crude assumptions made in this work. Achieving higher accuracy and specificity entails a better understanding of the emission patterns, and arguably requires much more extensive and accurate measurements of loads to appreciate their spatial and temporal variability, as well as their dependence on regionally varying drivers (such as the level of wastewater treatment, reported substance use etc.) across the European Union.

## Appendix A Supplementary data

Supplementary material 1 Image 1

## Footnotes

1 http://prtr.ec.europa.eu
