# Supplementary material for: River pollution by priority chemical substances under the Water Framework Directive: A provisional pan-European assessment
Source: Sci Total Environ. 2019 Apr 20;662:434–45. doi: 10.1016/j.scitotenv.2018.12.354 (PMC6391594; doi:10.1016/j.scitotenv.2018.12.354)
Supplement: Supplementary file 1 — Supplementary material 1 [file mmc1.docx]

Supporting information

Contents

[Summary of IPChem datasets: sample stations and observations 2](#_Toc535578586)

[Hydrological modelling 3](#_Toc535578587)

[Emission rates 5](#_Toc535578588)

[Model selection for the Pan-European study 8](#_Toc535578589)

[Statistics on point sources influence on overall pollution 15](#_Toc535578590)

[Point Emissions maps 20](#_Toc535578591)

[Comparison with national inventories 25](#_Toc535578592)

[Concentrations from diffuse sources (emissions patterns: agriculture and population) 29](#_Toc535578593)

[Total loads to the European seas 35](#_Toc535578594)

[Receiver-Operator Characteristics (ROC) curves of predicted and observed concentrations concentrations 42](#_Toc535578595)

[Statistics of model errors by EU Member states 56](#_Toc535578596)

All URLs cited in the text were last accessed at the time of publication of the paper.

## Summary of IPChem datasets: sample stations and observations

The following table summarizes the availability of observations in the IPChem datasets used in the study.

| **Chemical Name** | **Number of Stations pre-2009** | **Number of Stations post-2009** | **Number of observations pre-2009** | **Number of observations post-2009** | **Number of overlapping stations (same sub-basin)** |
| --- | --- | --- | --- | --- | --- |
| 1,2-dichloroethane | 440 | 2479 | 11171 | 46598 | 101 |
| Alachlor | 555 | 2401 | 11994 | 56082 | 120 |
| Anthracene | 355 | 2547 | 5794 | 49257 | 90 |
| Atrazine | 683 | 2469 | 16384 | 56206 | 124 |
| Benzene | 509 | 2472 | 10891 | 47057 | 101 |
| Bifenox | 353 | 833 | 8637 | 7322 | 171 |
| Cadmium (and inorganic compounds) | 1142 | 3035 | 28313 | 72167 | 113 |
| Chlorfenvinphos | 337 | 2385 | 4806 | 52914 | 84 |
| Chloroalkanes, C10-13 | 61 | 2026 | 539 | 36043 | 32 |
| Chloroform | 560 | 2489 | 12938 | 47262 | 100 |
| Chlorpyrifos | 403 | 2419 | 6981 | 53250 | 99 |
| Cypermethrin | 408 | 773 | 9525 | 6647 | 129 |
| Di(2-ethylhexyl)phthalate | 230 | 1984 | 2319 | 19872 | 51 |
| Dichloromethane | 413 | 2233 | 7952 | 44050 | 96 |
| Dichlorvos | 554 | 1360 | 13182 | 12470 | 230 |
| Dicofol | 336 | 1119 | 8676 | 8879 | 142 |
| Diuron | 380 | 2288 | 5705 | 52236 | 103 |
| Endosulfan | 199 | 992 | 3119 | 10116 | 31 |
| Fluoranthene | 527 | 2532 | 9666 | 48239 | 72 |
| Heptachlor (including epoxide) | 559 | 1412 | 13967 | 21151 | 195 |
| Hexachlorobenzene (HCB) | 641 | 2478 | 11303 | 51709 | 91 |
| Hexachlorobutadiene | 520 | 2388 | 9085 | 46371 | 74 |
| Hexachlorocyclohexane - all isomers | 147 | 967 | 731 | 10035 | 11 |
| Isoproturon | 360 | 2172 | 5590 | 51973 | 99 |
| Lead (and inorganic compounds) | 1018 | 3051 | 24291 | 73287 | 102 |
| Mercury (and inorganic compounds) | 1091 | 2881 | 26848 | 64213 | 101 |
| Naphtalene | 406 | 2498 | 7333 | 48491 | 90 |
| Nickel | 949 | 3024 | 23750 | 72003 | 98 |
| Nonylphenol | 108 | 181 | 1387 | 2190 | 0 |
| Pentachlorobenzene | 371 | 2360 | 6776 | 49675 | 77 |
| Pentachlorophenol | 463 | 2206 | 7817 | 49004 | 78 |
| Quinoxyfen | 261 | 1116 | 5643 | 9338 | 142 |
| Simazine | 650 | 2470 | 16187 | 56378 | 123 |
| Terbutryn | 673 | 1137 | 17649 | 10142 | 260 |
| Tributyltin compounds | 160 | 1602 | 1031 | 21567 | 64 |
| Trifluralin | 433 | 2388 | 8996 | 51202 | 89 |

Table 1 SI – summary of monitoring stations and data points available from IPCheM

## Hydrological modelling

The Budyko equation is well-established in the hydrological literature, and acknowledged to provide a satisfactory prediction when the target is long-term mean annual water availability in a natural river basin. The model has been validated against independent runoff measurements specifically for European scale applications. In this assessment, we refer to the variant proposed by Zhang et al., 2001^[[1]](#footnote-1)^:

| $S=P-\frac{P+wE_{0}}{1+w\frac{E_{0}}{P}+\frac{P}{E_{0}}}$ |  |
| --- | --- |

Where ET is long-term average annual evapotranspiration, P is long-term average annual precipitation, E_0_ is potential evapotranspiration and w is a parameter termed the plant-available water coefficient (Zhang et al., 2001) representing the relative difference in the way plants use soil water for transpiration. In this analysis, we assume the average of E_0_ and P over the period 1990-2010 to represent current conditions. The term $\frac{E_{0}}{P}$, sometimes called the ”aridity index”, depends only on the climate of a region, while w may be related in principle to vegetation and land cover. In this assessment we assume w=1, yielding a “midway” estimation reasonably representative of average European conditions. The model forcing was taken from *Ntegeka et al., 2013^[[2]](#footnote-2)^,* and E_0_ was computed with the Penman-Monteith model using the LISVAP code^[[3]](#footnote-3)^. The cumulative surplus in a catchment represents the annual average discharge Q, which can be computed for the sub-basins considered in this study.

The Budyko equation yields estimates of annual average water surplus which, integrated over the catchment area of each river segment, is an estimate of annual average river water flow. This compares very favorably with annual average discharges over the period 2005-2013 as estimated by the LISFLOOD model, as shown in the graph below. A discrepancy between Budyko and LISFLOOD estimates emerges only below annual average flows of a few m3/s, with a tendency of the Budyko model to predict higher flows.


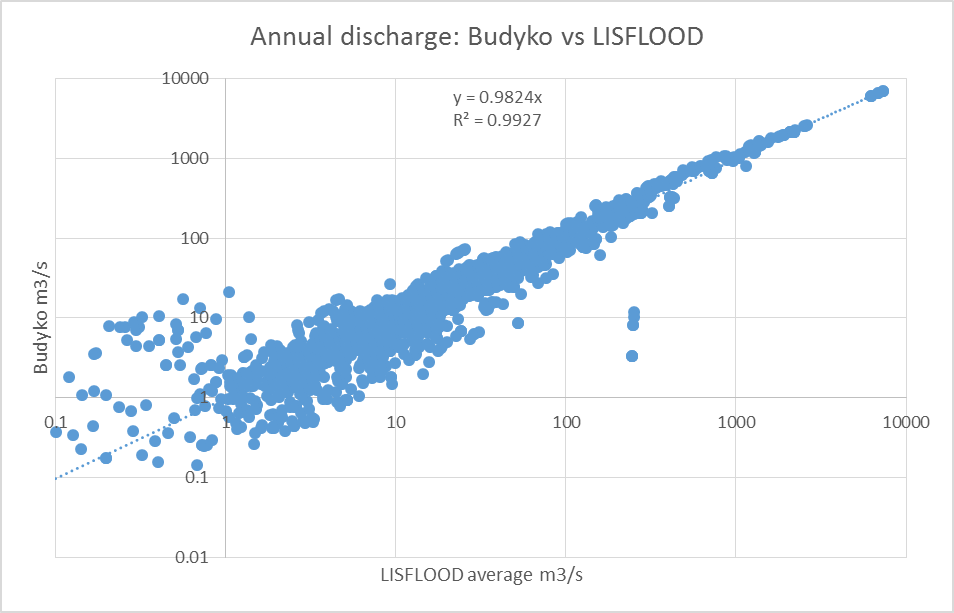


Figure 1 SI – comparison of annual average discharge for the period 2015-2013 from the calibrated LISFLOOD model and the Budyko equation

In order to understand the impacts of hydrological variability on the estimation of concentrations, it may be useful to compare the annual average river flow at a site with the daily flow at the same site during chemical monitoring, the latter being estimated again with the LISFLOOD model. While the correspondence is overall quite good also in this case, the dispersion of the values is apparently higher, as expected (see graph below).


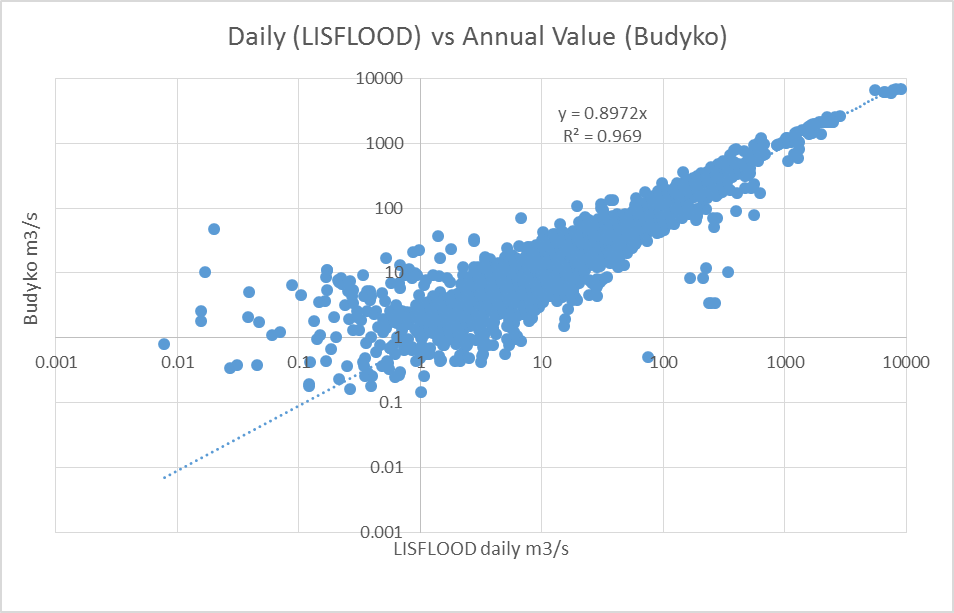


Figure 2 SI – comparison of discharge of the day of sampling from the calibrated LISFLOOD model, and the corresponding annual average discharge from Budyko equation. Each point represents a data point in IPChem (a date for a given monitoring station) for the pre-2009 samples.

The distribution of errors when estimating the daily flow with the corresponding annual average flow is shown in the following histogram. While the mode of the errors is between +/- 20%, about 85% of the cases is well within a factor 2 (+/-100%). This helps putting in perspective any modelling effort aimed at predicting concentrations: even a 100% accurate estimation of loads is likely to fall short of yielding concentrations more accurate than within a factor of 2, unless water flow at the time of sampling is known with sufficient accuracy.


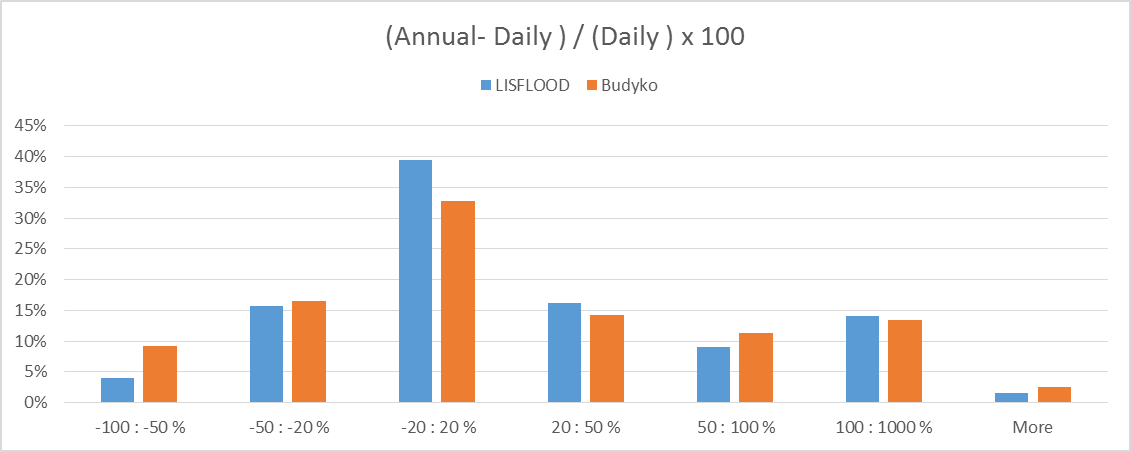


Figure 3 SI – distribution of errors in estimating daily discharge with annual average discharge, with the Budyko equation and the LISFLOOD average.

## Emission rates

Table 2 SI- Emission rates for the 37 Priority Substances considered in the Pan-European case study for different scenarios of DT50. Emissions are reported per person (ng/ind/s) or agricultural land (ng/km2/s). SD refers to Standard Deviation and was calculated as the standard deviation of the 1,000 bootstrap estimates. The reported emission value is also an average of the bootstrap realizations. In bold, most plausible emissions and DT50 values as identified during model selection.

|  |  | DT50 (days) | | | | | | | | |
| --- | --- | --- | --- | --- | --- | --- | --- | --- | --- | --- |
|  |  | 1 | 3 | 5 | 7 | 10 | 20 | 50 | 100 | 1000 |
| 1_2_dichloroethane | pop. ng/ind/s | 40.99 | 25.36 | 20.32 | 18.16 | **16.77** | 13.12 | 11.63 | 11.00 | 10.30 |
|  | SD | 9.18 | 5.34 | 4.83 | 4.28 | **4.13** | 3.15 | 2.91 | 2.77 | 2.54 |
|  | agri. ng/km2/s | 14758.03 | 6605.08 | 5001.82 | **4387.40** | 3920.19 | 3341.36 | 2906.06 | 2760.46 | 2485.56 |
|  | SD | 2252.78 | 1233.04 | 972.76 | **906.67** | 839.38 | 670.73 | 632.29 | 577.47 | 549.74 |
| Alachlor | pop. ng/ind/s | 2.01 | 1.30 | 1.11 | 1.01 | 0.94 | **0.84** | 0.77 | 0.74 | 0.70 |
|  | SD | 0.30 | 0.17 | 0.14 | 0.12 | 0.12 | **0.11** | 0.10 | 0.10 | 0.10 |
|  | agri. ng/km2/s | 695.92 | 373.77 | 301.36 | 264.68 | 239.37 | **203.70** | 179.75 | 169.70 | 154.07 |
|  | SD | 93.89 | 41.62 | 31.90 | 28.72 | 26.85 | **25.13** | 24.35 | 23.47 | 22.28 |
| Anthracene | pop. ng/ind/s | 0.70 | 0.42 | 0.34 | **0.31** | 0.27 | 0.22 | 0.20 | 0.18 | 0.17 |
|  | SD | 0.15 | 0.09 | 0.07 | **0.07** | 0.07 | 0.06 | 0.06 | 0.06 | 0.06 |
|  | agri. ng/km2/s | 220.40 | 102.23 | 78.94 | 69.34 | **61.48** | 51.20 | 45.06 | 41.02 | 38.18 |
|  | SD | 34.26 | 16.40 | 14.33 | 13.39 | **12.92** | 11.34 | 10.98 | 11.14 | 10.97 |
| Atrazine | pop. ng/ind/s | 3.86 | 2.54 | 2.13 | 1.95 | 1.80 | 1.61 | 1.49 | 1.44 | **1.38** |
|  | SD | 0.46 | 0.27 | 0.21 | 0.20 | 0.19 | 0.17 | 0.16 | 0.16 | **0.15** |
|  | agri. ng/km2/s | 1487.72 | 697.49 | 546.68 | 483.17 | 441.22 | 389.13 | 354.18 | 336.05 | **325.11** |
|  | SD | 159.22 | 79.87 | 63.45 | 55.77 | 52.22 | 45.83 | 43.23 | 40.34 | **38.72** |
| Benzene | pop. ng/ind/s | 30.87 | 20.70 | 16.98 | 15.39 | 13.95 | 11.75 | 10.71 | 9.93 | **9.57** |
|  | SD | 5.74 | 3.58 | 3.17 | 2.96 | 2.76 | 2.52 | 2.51 | 2.37 | **2.24** |
|  | agri. ng/km2/s | 11823.62 | 5237.03 | **3949.77** | 3419.53 | 2999.16 | 2397.67 | 2026.50 | 1905.89 | 1763.92 |
|  | SD | 1720.59 | 885.39 | **735.32** | 657.67 | 557.02 | 524.55 | 491.09 | 447.03 | 434.23 |
| Bifenox | pop. ng/ind/s | 4.61 | 2.52 | 2.07 | 1.95 | 1.78 | 1.71 | 1.59 | 1.57 | **1.58** |
|  | SD | 1.67 | 0.89 | 0.73 | 0.72 | 0.71 | 0.68 | 0.66 | 0.65 | **0.63** |
|  | agri. ng/km2/s | 1586.34 | 598.08 | 452.01 | 399.15 | 356.82 | 313.65 | 290.90 | 281.59 | **277.16** |
|  | SD | 144.91 | 68.85 | 58.46 | 58.81 | 53.93 | 52.80 | 49.23 | 50.23 | **49.69** |
| Cadmium | pop. ng/ind/s | 30.16 | 24.42 | 23.75 | 23.98 | 24.57 | 24.68 | 24.53 | 24.25 | **23.59** |
|  | SD | 5.52 | 5.05 | 5.56 | 6.22 | 7.43 | 7.97 | 8.14 | 7.93 | **7.95** |
|  | agri. ng/km2/s | 15222.99 | 8483.09 | 7401.22 | 7221.09 | 6864.73 | 6231.94 | **5603.22** | 5475.76 | 4897.06 |
|  | SD | 2395.83 | 1632.46 | 1730.91 | 1859.82 | 1940.30 | 1926.39 | **1857.33** | 1755.40 | 1564.66 |
| Chlorfenvinphos | pop. ng/ind/s | 1.19 | 0.64 | 0.52 | 0.46 | 0.42 | 0.37 | 0.34 | **0.32** | 0.31 |
|  | SD | 0.25 | 0.09 | 0.06 | 0.05 | 0.04 | 0.04 | 0.03 | **0.03** | 0.03 |
|  | agri. ng/km2/s | 447.56 | 182.82 | 136.17 | 119.71 | 106.91 | 92.03 | 83.07 | **78.17** | 72.53 |
|  | SD | 61.84 | 22.34 | 16.91 | 15.74 | 12.87 | 11.64 | 10.63 | **9.74** | 9.19 |
| Chloroalkanes_C10_13 | pop. ng/ind/s | 279.92 | 426.14 | 343.22 | 317.65 | 296.62 | 261.15 | 234.45 | 226.23 | **218.74** |
|  | SD | 260.27 | 232.95 | 165.90 | 135.38 | 116.92 | 97.80 | 86.31 | 78.72 | **77.58** |
|  | agri. ng/km2/s | 274880.36 | 138600.89 | 93802.32 | 77639.33 | 68440.31 | 56610.00 | 50162.55 | **47444.20** | 44876.21 |
|  | SD | 125887.92 | 42940.66 | 26352.84 | 21090.76 | 17316.52 | 13060.30 | 11381.13 | **10739.43** | 10237.20 |
| Chloroform | pop. ng/ind/s | 18.15 | 13.36 | 11.54 | 10.65 | **9.29** | 7.82 | 6.76 | 6.52 | 6.16 |
|  | SD | 4.18 | 3.24 | 2.92 | 2.74 | **2.54** | 2.25 | 2.17 | 2.12 | 2.09 |
|  | agri. ng/km2/s | 9285.67 | 4385.08 | 3304.13 | **2852.11** | 2527.61 | 2026.49 | 1740.39 | 1622.59 | 1496.14 |
|  | SD | 1477.00 | 860.72 | 680.31 | **657.88** | 578.55 | 509.41 | 475.97 | 456.94 | 399.30 |
| Chlorpyrifos | pop. ng/ind/s | 1.51 | 0.75 | 0.60 | 0.52 | **0.48** | 0.42 | 0.38 | 0.37 | 0.35 |
|  | SD | 0.20 | 0.10 | 0.07 | 0.06 | **0.06** | 0.05 | 0.05 | 0.05 | 0.04 |
|  | agri. ng/km2/s | 485.09 | 198.03 | 150.26 | 130.93 | **118.14** | 101.28 | 90.94 | 86.73 | 83.37 |
|  | SD | 59.31 | 24.35 | 17.80 | 16.07 | **14.20** | 12.67 | 11.35 | 10.90 | 10.47 |
| Cypermethrin | pop. ng/ind/s | 2.82 | 1.24 | 0.96 | 0.87 | 0.81 | 0.71 | 0.69 | 0.67 | **0.68** |
|  | SD | 0.82 | 0.45 | 0.40 | 0.38 | 0.35 | 0.35 | 0.32 | 0.30 | **0.28** |
|  | agri. ng/km2/s | 675.40 | 274.62 | 207.22 | 180.85 | 161.68 | 142.54 | 130.68 | 125.48 | **128.62** |
|  | SD | 71.06 | 30.83 | 27.65 | 25.93 | 25.44 | 24.74 | 22.02 | 22.71 | **22.50** |
| Di_2_ethylhexyl_phthalate | pop. ng/ind/s | 129.48 | 65.62 | 52.59 | 46.46 | 42.96 | 37.54 | 34.57 | 33.04 | **31.43** |
|  | SD | 35.83 | 16.90 | 12.51 | 10.90 | 10.05 | 8.06 | 7.46 | 6.64 | **6.88** |
|  | agri. ng/km2/s | 39418.34 | 16743.69 | 12696.36 | 11409.40 | 10009.30 | 8613.39 | 7849.06 | 7502.63 | **7124.62** |
|  | SD | 8826.52 | 3391.44 | 2413.55 | 2044.04 | 1870.12 | 1577.40 | 1455.14 | 1435.88 | **1305.17** |
| Dichloromethane | pop. ng/ind/s | 95.81 | 59.40 | 49.36 | 43.57 | **38.85** | 33.25 | 29.75 | 27.93 | 25.89 |
|  | SD | 23.98 | 11.97 | 10.03 | 9.40 | **8.88** | 7.82 | 7.25 | 7.54 | 7.33 |
|  | agri. ng/km2/s | 32702.44 | 15438.88 | 11691.00 | **10293.35** | 9097.07 | 7423.84 | 6297.84 | 5938.52 | 5482.18 |
|  | SD | 6407.85 | 3153.92 | 2469.09 | **2408.35** | 2223.15 | 1890.04 | 1626.54 | 1649.52 | 1584.66 |
| Dichlorvos | pop. ng/ind/s | 1.15 | 0.78 | 0.70 | 0.66 | 0.64 | 0.60 | 0.58 | 0.56 | **0.55** |
|  | SD | 0.19 | 0.13 | 0.11 | 0.12 | 0.11 | 0.11 | 0.11 | 0.11 | **0.11** |
|  | agri. ng/km2/s | 725.93 | 335.52 | 267.85 | 242.23 | 215.59 | 190.47 | 175.54 | 171.26 | **164.43** |
|  | SD | 69.96 | 34.40 | 34.21 | 33.52 | 32.14 | 31.15 | 31.69 | 31.30 | **29.96** |
| Dicofol | pop. ng/ind/s | 3.07 | 2.06 | 1.82 | 1.71 | **1.64** | 1.51 | 1.43 | 1.40 | 1.36 |
|  | SD | 1.12 | 0.62 | 0.47 | 0.41 | **0.35** | 0.29 | 0.25 | 0.24 | 0.21 |
|  | agri. ng/km2/s | 629.78 | 277.00 | 218.95 | 193.68 | 181.41 | 160.24 | 151.64 | 147.59 | **145.24** |
|  | SD | 65.11 | 33.35 | 28.07 | 28.76 | 28.11 | 25.77 | 26.23 | 24.02 | **23.66** |
| Diuron | pop. ng/ind/s | 7.46 | 4.67 | 3.73 | 3.37 | 3.03 | 2.63 | 2.35 | 2.20 | **2.08** |
|  | SD | 1.35 | 0.79 | 0.64 | 0.55 | 0.53 | 0.45 | 0.44 | 0.44 | **0.43** |
|  | agri. ng/km2/s | 2565.08 | 1104.09 | 836.11 | 722.74 | 647.51 | **549.07** | 490.86 | 461.84 | 430.81 |
|  | SD | 392.23 | 194.22 | 148.30 | 133.79 | 118.67 | **109.19** | 94.04 | 92.01 | 85.55 |
| Endosulfan | pop. ng/ind/s | 1.47 | 0.73 | 0.59 | 0.52 | **0.48** | 0.13 | 0.13 | 0.12 | 0.12 |
|  | SD | 0.23 | 0.13 | 0.11 | 0.11 | **0.10** | 0.08 | 0.06 | 0.06 | 0.06 |
|  | agri. ng/km2/s | 482.96 | 181.47 | 135.92 | 116.57 | **100.82** | 72.56 | 60.86 | 55.75 | 48.73 |
|  | SD | 88.37 | 39.47 | 29.63 | 27.54 | **24.33** | 18.87 | 16.77 | 16.39 | 15.42 |
| Fluoranthene | pop. ng/ind/s | 2.24 | 1.67 | 1.40 | 1.27 | **1.15** | 0.74 | 0.69 | 0.69 | 0.65 |
|  | SD | 0.56 | 0.33 | 0.27 | 0.24 | **0.23** | 0.24 | 0.22 | 0.21 | 0.19 |
|  | agri. ng/km2/s | 601.60 | 311.75 | 241.31 | 213.31 | **187.75** | 153.07 | 134.74 | 127.66 | 112.31 |
|  | SD | 113.10 | 58.24 | 44.16 | 39.24 | **34.95** | 29.90 | 25.55 | 24.96 | 21.57 |
| Heptachlor | pop. ng/ind/s | 0.33 | 0.14 | 0.11 | 0.10 | 0.09 | 0.08 | 0.08 | 0.08 | **0.08** |
|  | SD | 0.06 | 0.03 | 0.03 | 0.02 | 0.02 | 0.02 | 0.02 | 0.02 | **0.02** |
|  | agri. ng/km2/s | 151.52 | **55.50** | 40.39 | 34.58 | 30.60 | 26.15 | 23.23 | 22.64 | 21.47 |
|  | SD | 20.86 | **7.89** | 6.54 | 5.60 | 5.30 | 4.84 | 4.45 | 4.26 | 4.12 |
| Hexachlorobenzene | pop. ng/ind/s | 0.36 | 0.29 | 0.26 | 0.24 | 0.22 | 0.20 | 0.18 | 0.17 | **0.16** |
|  | SD | 0.08 | 0.05 | 0.05 | 0.04 | 0.04 | 0.04 | 0.03 | 0.03 | **0.03** |
|  | agri. ng/km2/s | 174.33 | 89.47 | 70.53 | **62.36** | 54.98 | 46.02 | 39.62 | 37.55 | 33.56 |
|  | SD | 31.89 | 14.48 | 11.57 | **10.44** | 9.74 | 8.59 | 7.57 | 7.58 | 6.62 |
| Hexachlorobutadiene | pop. ng/ind/s | 2.44 | 2.04 | 1.81 | 1.64 | 1.55 | 1.34 | 1.19 | 1.15 | **1.07** |
|  | SD | 0.63 | 0.50 | 0.45 | 0.42 | 0.40 | 0.36 | 0.36 | 0.36 | **0.36** |
|  | agri. ng/km2/s | 1590.53 | 777.20 | 581.72 | **501.59** | 445.51 | 367.85 | 304.97 | 290.10 | 262.56 |
|  | SD | 334.79 | 163.86 | 132.61 | **119.01** | 116.92 | 101.06 | 91.48 | 95.62 | 85.95 |
| Hexachlorocyclohexane | pop. ng/ind/s | 1.50 | 0.89 | 0.68 | **0.57** | 0.50 | 0.42 | 0.37 | 0.35 | 0.33 |
|  | SD | 0.51 | 0.23 | 0.15 | **0.14** | 0.11 | 0.12 | 0.11 | 0.11 | 0.10 |
|  | agri. ng/km2/s | 286.95 | 150.41 | 113.91 | 96.91 | 84.60 | **70.24** | 61.14 | 57.12 | 49.85 |
|  | SD | 59.03 | 24.52 | 16.14 | 13.06 | 11.33 | **9.97** | 8.68 | 7.67 | 6.96 |
| Isoproturon | pop. ng/ind/s | 8.31 | 4.81 | 3.91 | 3.47 | 3.12 | 2.67 | 2.40 | 2.27 | **2.16** |
|  | SD | 1.39 | 0.75 | 0.56 | 0.52 | 0.47 | 0.42 | 0.41 | 0.39 | **0.37** |
|  | agri. ng/km2/s | 2169.74 | 988.76 | 765.61 | 677.54 | 602.82 | 527.59 | 469.98 | 450.51 | **418.35** |
|  | SD | 359.99 | 176.75 | 136.08 | 117.40 | 104.12 | 90.71 | 84.02 | 75.85 | **75.36** |
| Lead | pop. ng/ind/s | 295.74 | 225.48 | 211.05 | 203.06 | 196.17 | 177.85 | 160.27 | 151.35 | **142.03** |
|  | SD | 46.54 | 40.06 | 40.75 | 41.04 | 37.99 | 33.52 | 27.83 | 27.25 | **24.50** |
|  | agri. ng/km2/s | 107148.92 | 57583.11 | 50574.24 | 46332.81 | 43409.50 | 36702.26 | 31189.19 | **28911.85** | 26168.76 |
|  | SD | 18690.60 | 12407.44 | 10883.06 | 9042.81 | 8302.48 | 6748.56 | 5360.98 | **5095.94** | 4509.06 |
| Mercury | pop. ng/ind/s | 17.18 | 10.70 | 9.00 | 8.31 | 7.72 | 6.08 | 5.23 | **4.85** | 4.38 |
|  | SD | 2.88 | 1.78 | 1.41 | 1.30 | 1.13 | 0.81 | 0.64 | **0.54** | 0.49 |
|  | agri. ng/km2/s | 4721.46 | 2451.52 | 2018.60 | 1785.04 | 1582.93 | 1250.44 | 992.26 | **902.84** | 768.26 |
|  | SD | 552.62 | 301.07 | 248.86 | 213.89 | 193.93 | 128.95 | 106.89 | **99.48** | 87.27 |
| Naphtalene | pop. ng/ind/s | 7.43 | 3.59 | **2.72** | 2.31 | 2.04 | 1.70 | 1.47 | 1.38 | 1.33 |
|  | SD | 1.26 | 0.49 | **0.40** | 0.37 | 0.33 | 0.31 | 0.30 | 0.30 | 0.29 |
|  | agri. ng/km2/s | 1722.60 | 697.03 | 512.46 | **436.37** | 381.49 | 316.84 | 275.03 | 261.17 | 241.48 |
|  | SD | 314.44 | 128.85 | 104.58 | **92.35** | 85.64 | 72.60 | 65.37 | 65.46 | 59.75 |
| Nickel | pop. ng/ind/s | 470.46 | 274.30 | 228.52 | 207.05 | 186.41 | 155.86 | 134.87 | **127.56** | 115.89 |
|  | SD | 52.33 | 24.95 | 23.03 | 23.60 | 22.72 | 18.69 | 15.95 | **14.80** | 12.97 |
|  | agri. ng/km2/s | 157453.13 | 69122.59 | 52579.49 | 45709.11 | 39823.29 | 31472.03 | 25583.79 | **23808.44** | 21203.00 |
|  | SD | 16118.57 | 7444.15 | 6822.24 | 6144.51 | 5421.90 | 4258.31 | 3382.61 | **2836.12** | 2513.88 |
| Nonylphenol | pop. ng/ind/s | 35.97 | 33.04 | 31.24 | 30.51 | 30.24 | 29.42 | 26.02 | 25.07 | **24.05** |
|  | SD | 27.13 | 25.64 | 22.51 | 22.38 | 21.87 | 20.69 | 18.20 | 18.48 | **17.51** |
|  | agri. ng/km2/s | 10509.09 | 7389.96 | 6663.01 | 6164.47 | 5906.57 | 5159.12 | 4449.15 | 4257.53 | **3899.53** |
|  | SD | 6539.08 | 5012.02 | 4844.57 | 4289.30 | 4306.76 | 3666.95 | 3419.05 | 3027.88 | **2960.30** |
| Pentachlorobenzene | pop. ng/ind/s | 1.03 | 0.68 | 0.59 | 0.55 | 0.51 | 0.47 | 0.43 | 0.42 | **0.40** |
|  | SD | 0.25 | 0.14 | 0.11 | 0.09 | 0.08 | 0.08 | 0.06 | 0.06 | **0.06** |
|  | agri. ng/km2/s | 397.27 | 208.14 | 167.37 | 149.80 | 136.08 | **114.70** | 103.88 | 98.18 | 88.44 |
|  | SD | 83.75 | 39.77 | 31.40 | 28.29 | 25.26 | **22.03** | 19.36 | 18.75 | 16.55 |
| Pentachlorophenol | pop. ng/ind/s | 3.02 | **1.31** | 0.96 | 0.82 | 0.71 | 0.48 | 0.43 | 0.42 | 0.40 |
|  | SD | 0.59 | **0.27** | 0.20 | 0.18 | 0.16 | 0.12 | 0.11 | 0.11 | 0.11 |
|  | agri. ng/km2/s | 871.30 | **299.00** | 205.63 | 169.35 | 147.14 | 118.25 | 97.71 | 91.89 | 81.20 |
|  | SD | 152.07 | **54.56** | 40.01 | 34.95 | 34.01 | 30.07 | 27.41 | 26.33 | 24.53 |
| Quinoxyfen | pop. ng/ind/s | 2.37 | 1.58 | 1.44 | 1.34 | 1.32 | 1.26 | 1.24 | 1.23 | **1.26** |
|  | SD | 1.30 | 0.94 | 0.77 | 0.73 | 0.64 | 0.63 | 0.57 | 0.54 | **0.50** |
|  | agri. ng/km2/s | 1114.20 | 427.97 | 335.44 | 295.01 | 265.98 | 240.43 | 223.83 | 217.66 | **230.04** |
|  | SD | 184.66 | 76.96 | 58.44 | 50.76 | 55.05 | 45.38 | 47.52 | 41.49 | **51.95** |
| Simazine | pop. ng/ind/s | 3.07 | 1.80 | 1.45 | 1.29 | **1.17** | 1.00 | 0.89 | 0.85 | 0.80 |
|  | SD | 0.45 | 0.25 | 0.20 | 0.20 | **0.20** | 0.19 | 0.17 | 0.17 | 0.17 |
|  | agri. ng/km2/s | 997.60 | 435.59 | 332.51 | **289.13** | 257.20 | 218.24 | 193.28 | 181.56 | 170.27 |
|  | SD | 124.40 | 62.45 | 51.99 | **47.32** | 43.34 | 39.87 | 36.94 | 39.08 | 35.20 |
| Terbutryn | pop. ng/ind/s | 2.59 | 1.45 | 1.24 | 1.15 | 1.06 | 0.98 | 0.94 | 0.91 | **0.90** |
|  | SD | 0.39 | 0.21 | 0.19 | 0.18 | 0.17 | 0.16 | 0.15 | 0.16 | **0.14** |
|  | agri. ng/km2/s | 931.15 | 400.07 | 308.88 | 270.95 | 245.05 | 215.33 | 198.37 | 192.40 | **186.82** |
|  | SD | 90.39 | 33.52 | 27.67 | 25.95 | 25.25 | 22.22 | 21.08 | 21.04 | **19.45** |
| Tributyltin | pop. ng/ind/s | 0.53 | 0.27 | 0.21 | 0.19 | 0.17 | **0.15** | 0.14 | 0.13 | 0.13 |
|  | SD | 0.09 | 0.05 | 0.04 | 0.03 | 0.03 | **0.02** | 0.02 | 0.02 | 0.02 |
|  | agri. ng/km2/s | 155.66 | 71.21 | 54.47 | 47.46 | 42.37 | **36.08** | 31.88 | 30.32 | 28.56 |
|  | SD | 31.98 | 11.79 | 7.34 | 5.98 | 4.88 | **3.96** | 3.30 | 3.21 | 2.90 |
| Trifluralin | pop. ng/ind/s | 1.06 | 0.58 | 0.45 | 0.40 | 0.36 | 0.31 | 0.28 | 0.27 | **0.26** |
|  | SD | 0.19 | 0.08 | 0.06 | 0.05 | 0.05 | 0.04 | 0.04 | 0.04 | **0.04** |
|  | agri. ng/km2/s | 370.35 | 152.13 | 114.00 | 99.17 | **87.83** | 76.26 | 66.46 | 62.40 | 58.79 |
|  | SD | 48.08 | 21.78 | 16.98 | 15.05 | **13.52** | 12.54 | 11.11 | 10.73 | 10.28 |

## Model selection for the Pan-European study


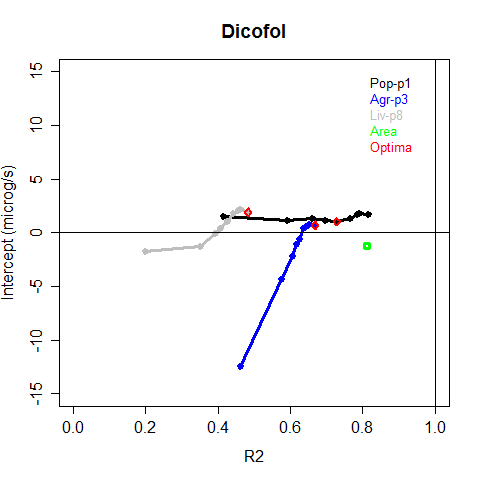

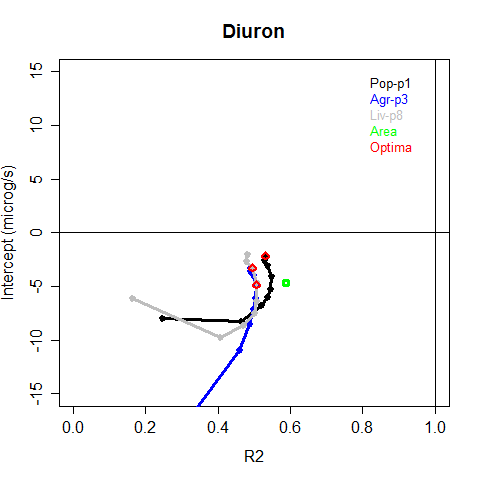

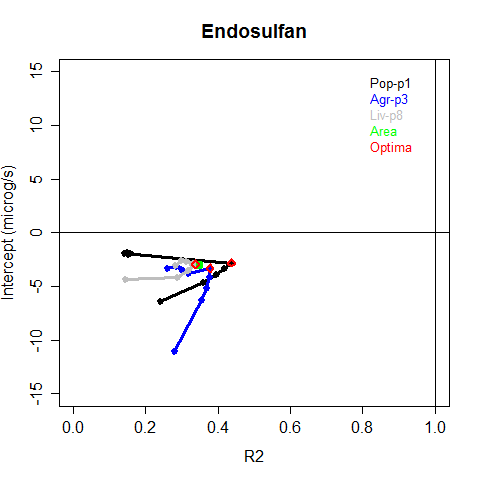

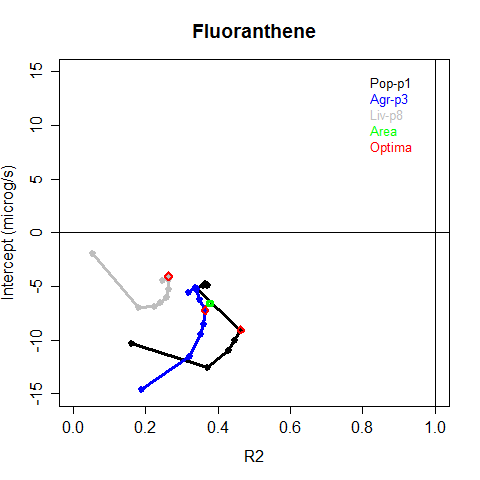

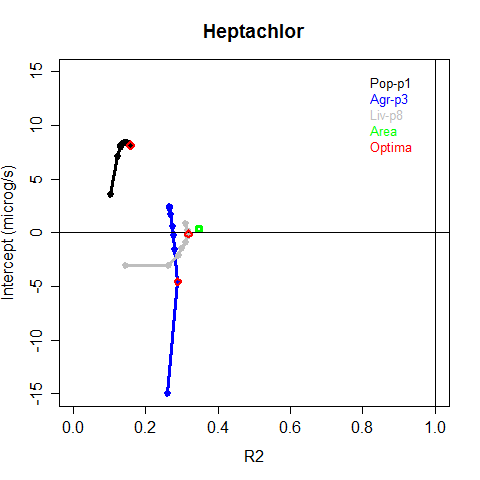

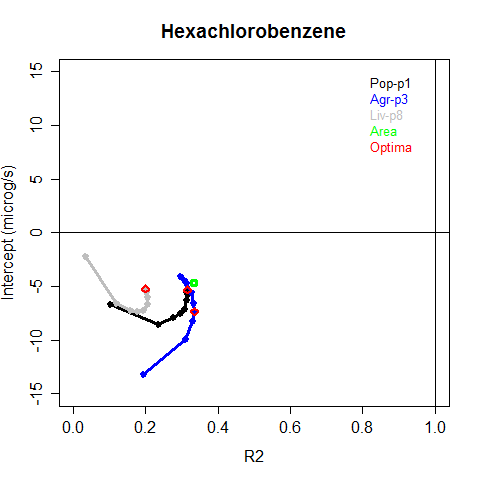

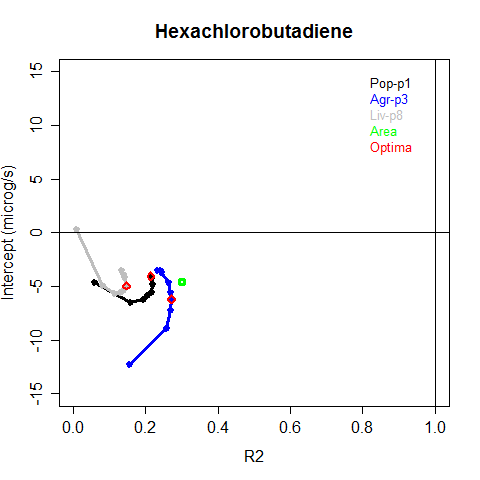

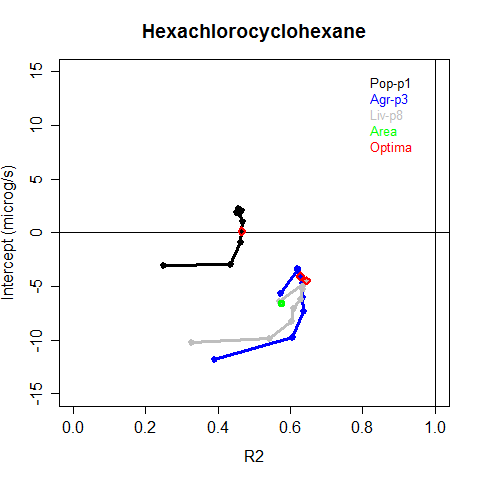

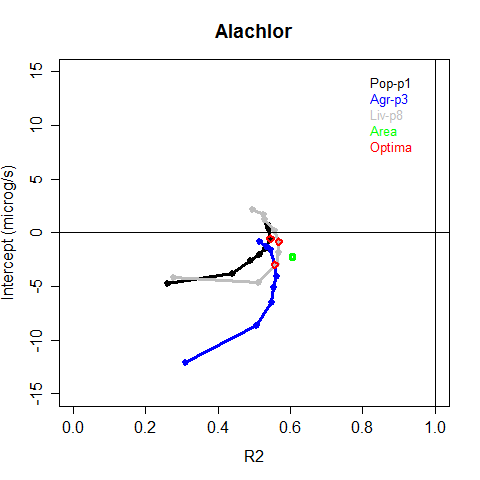

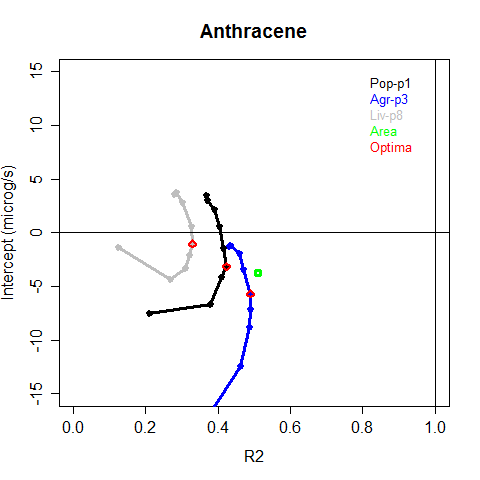

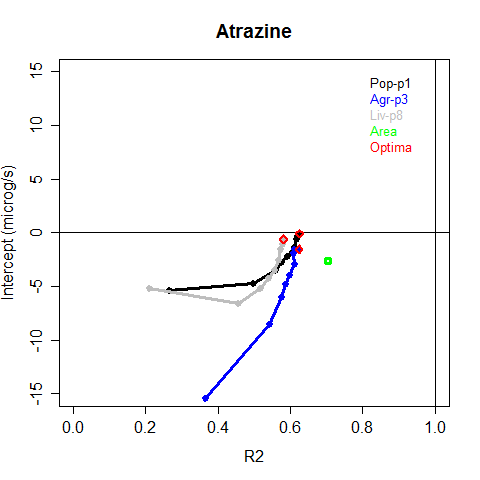

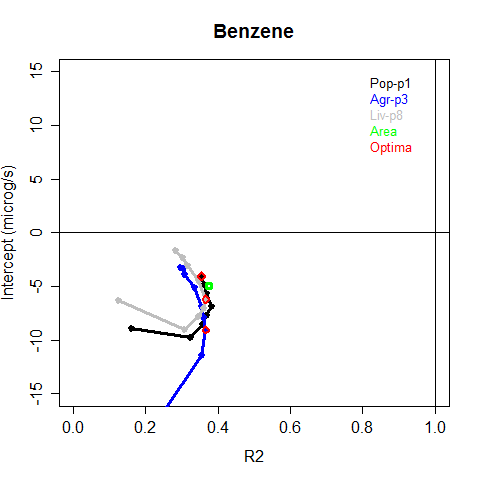

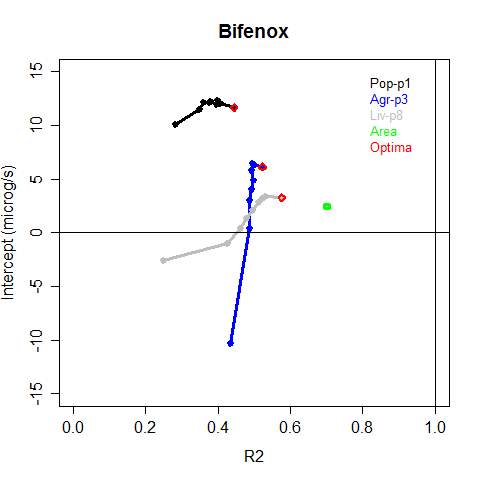

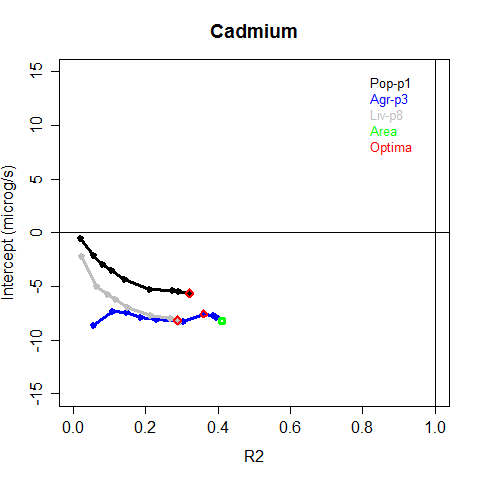

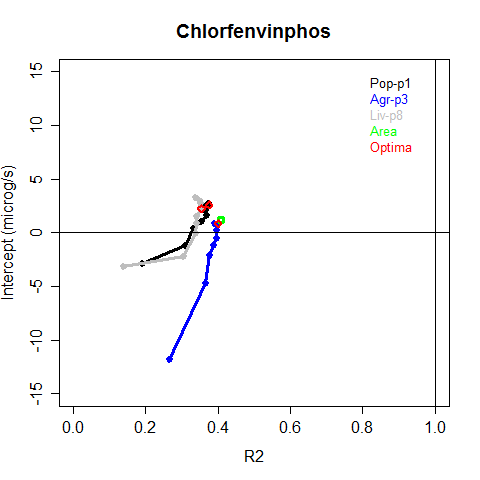

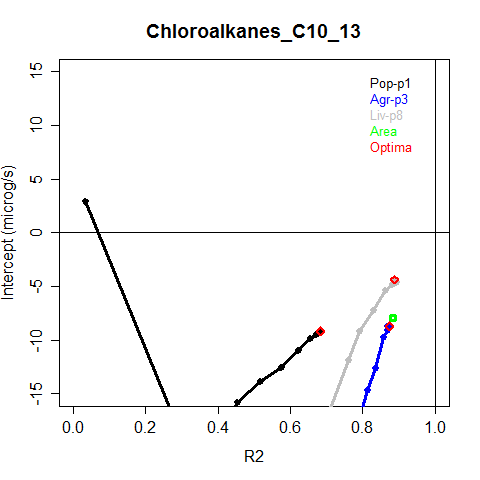

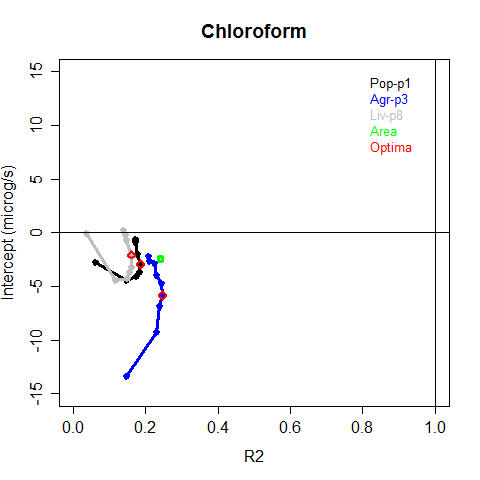

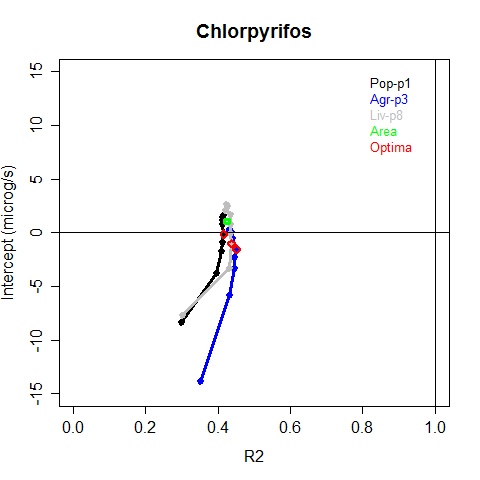

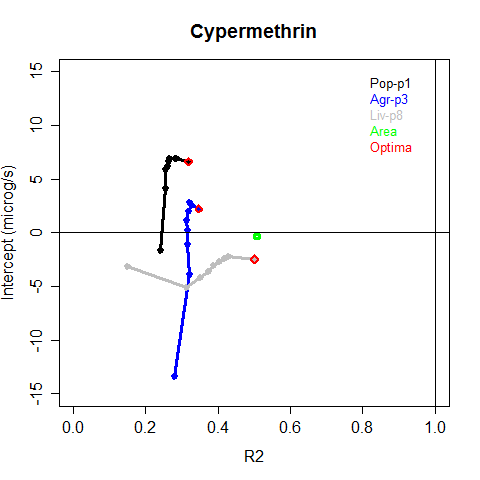

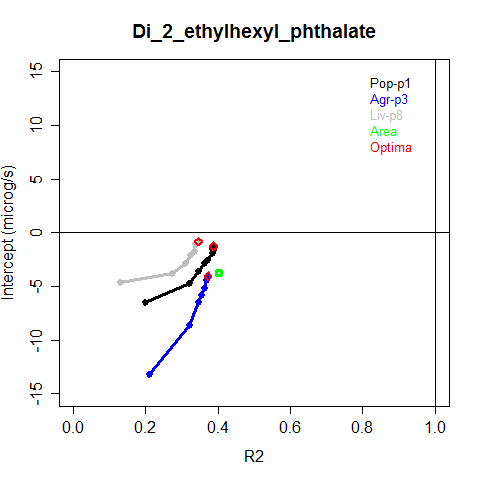

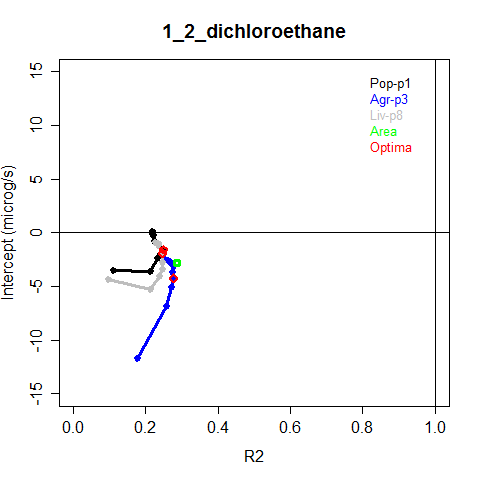

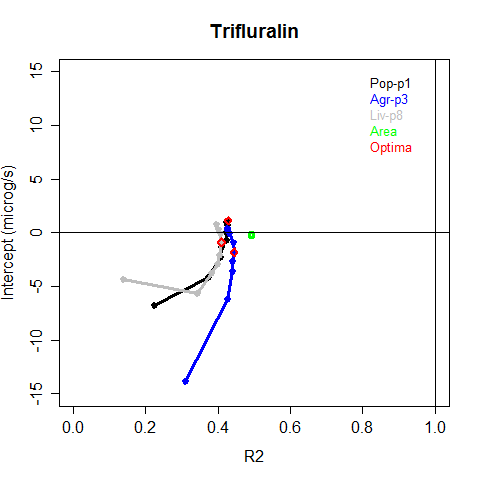

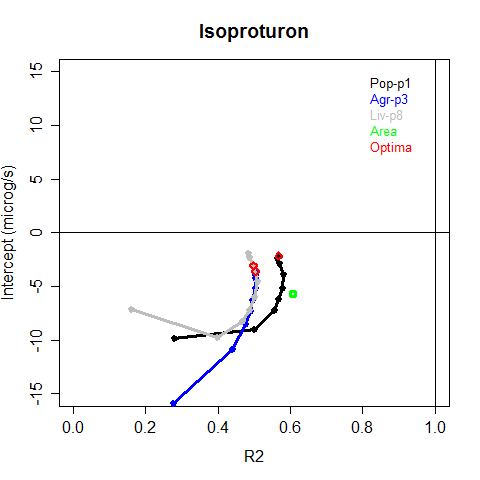

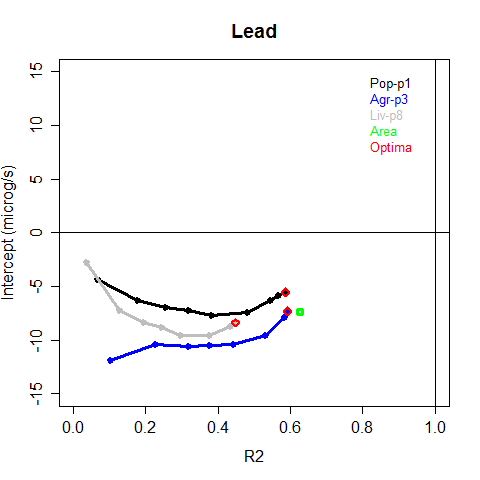

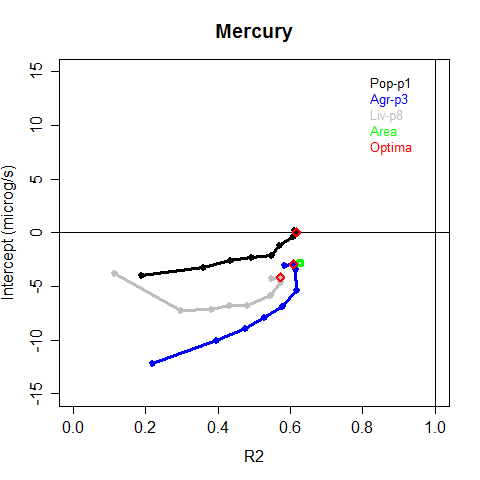

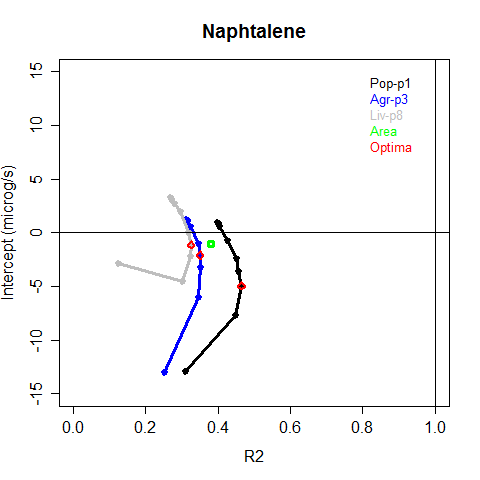

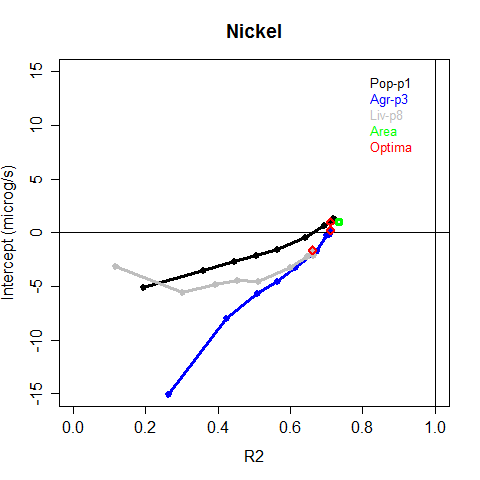

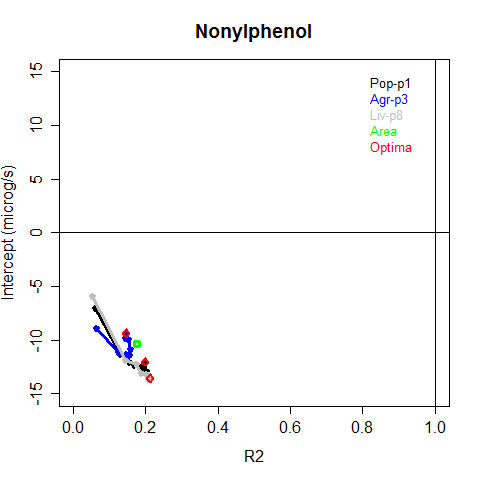

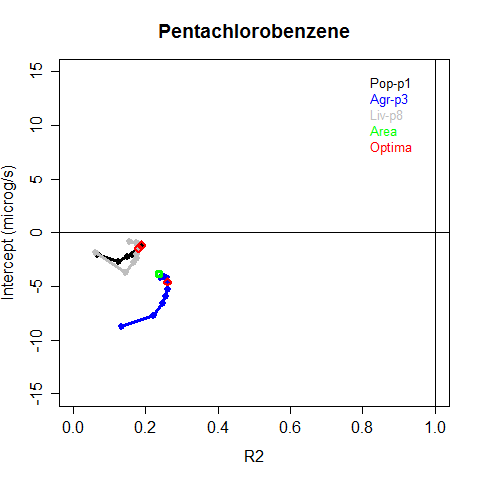

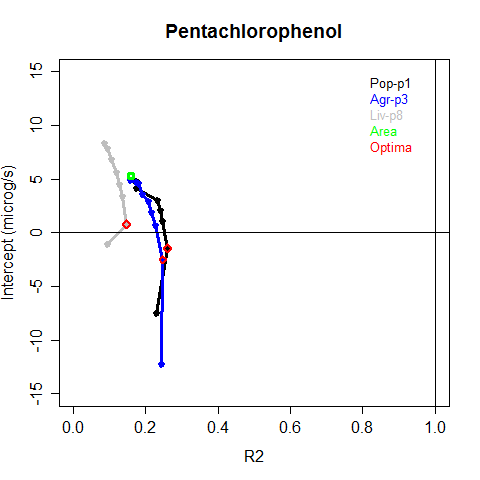

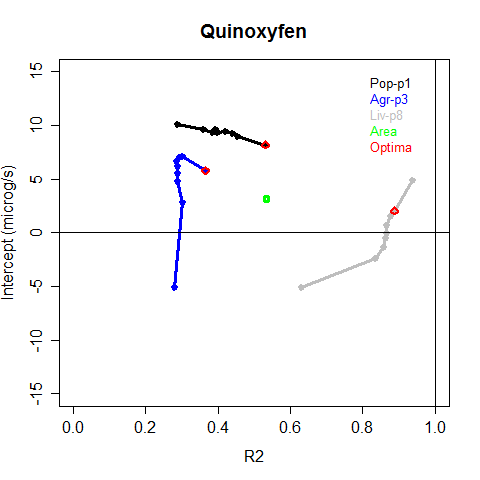

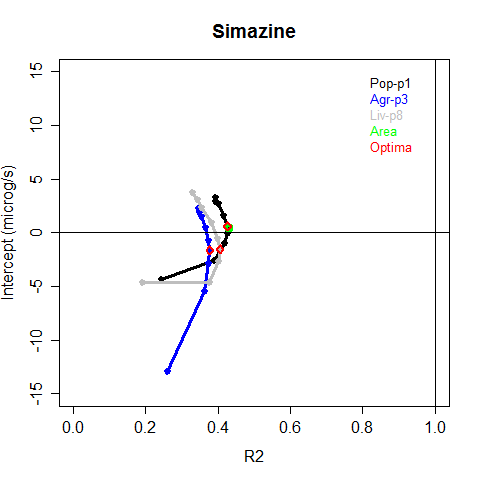

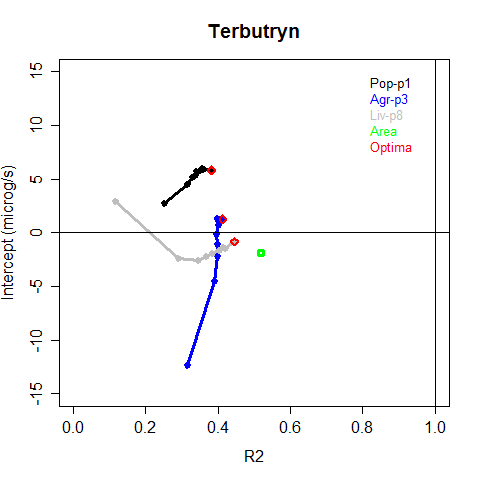

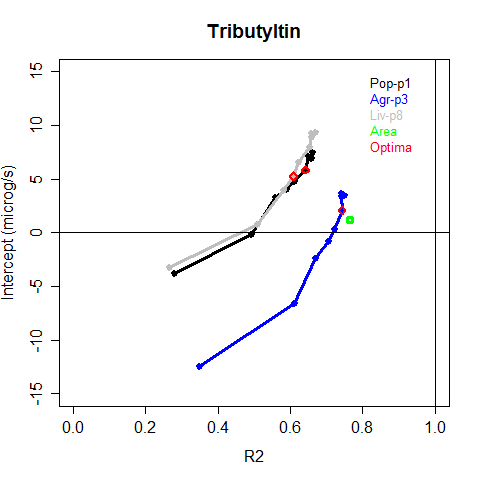

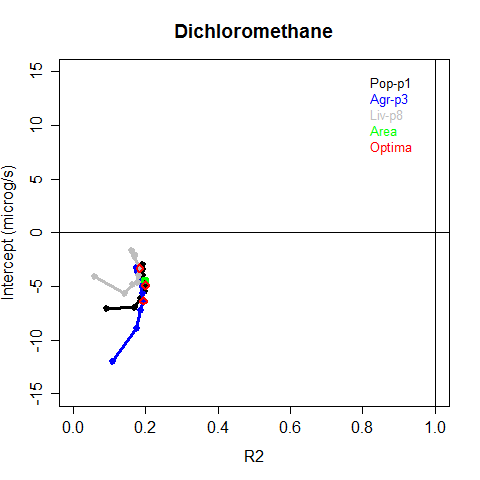

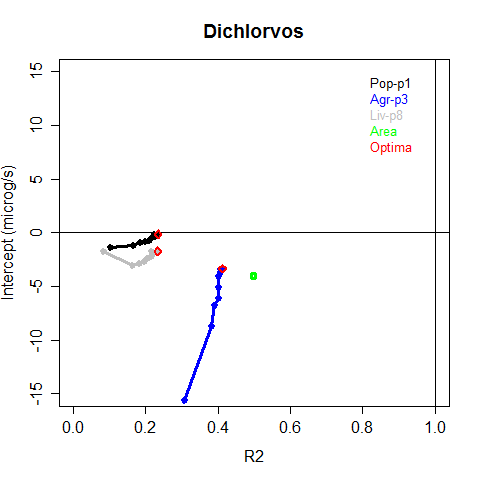


## Statistics on point sources influence on overall pollution

In the following graphs, we plot the distribution of the indicators adopted to describe the impact of point sources on overall pollution by priority substances: the maximum contribution of point source loads to calculated loads, $Z=max(\text{y}_{\text{1}},\ldots\text{y}_{\text{i}})$, where $y=\frac{\frac{L_{point}}{L_{diff}}}{1 + \frac{L_{point}}{L_{diff}}}$, and the number (W) of pollutants with Z value greater than 0.25.


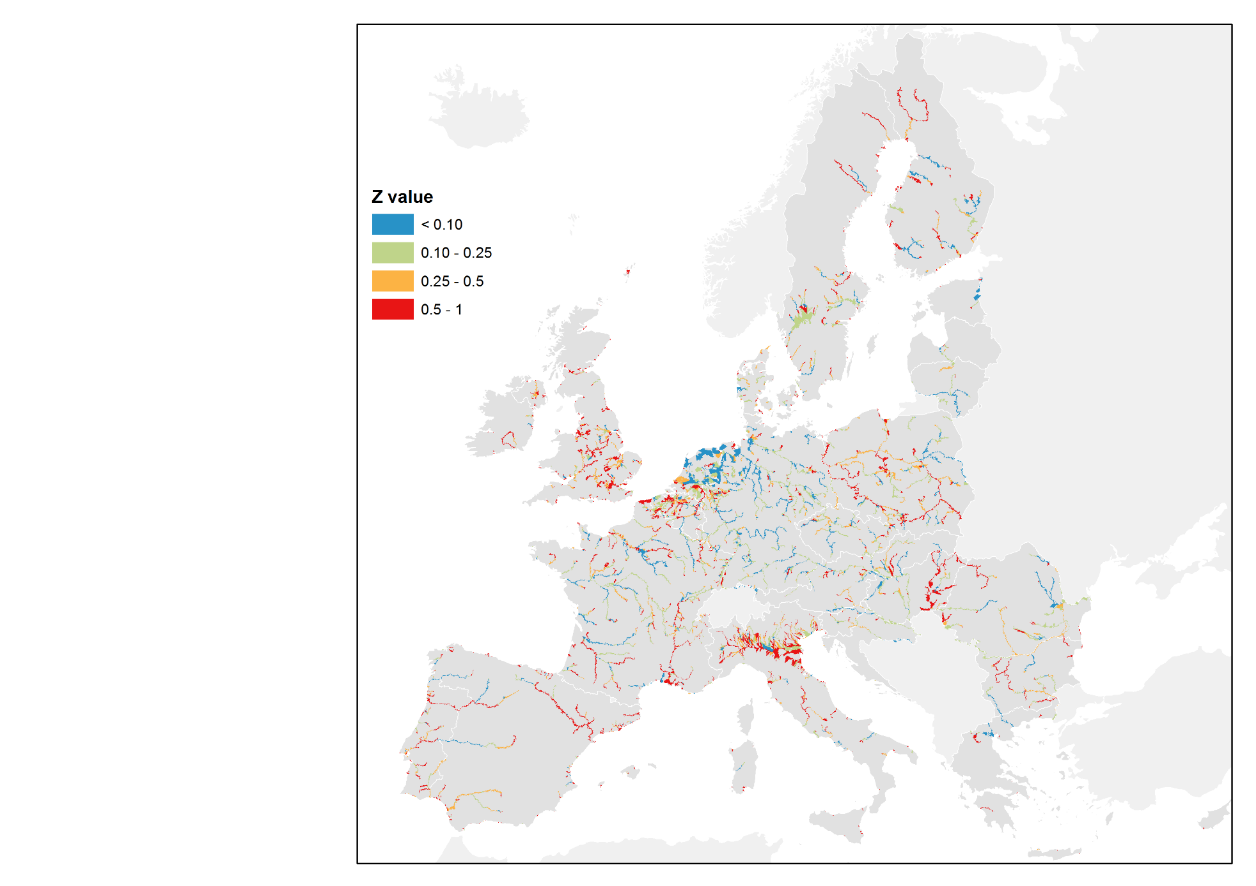
 a


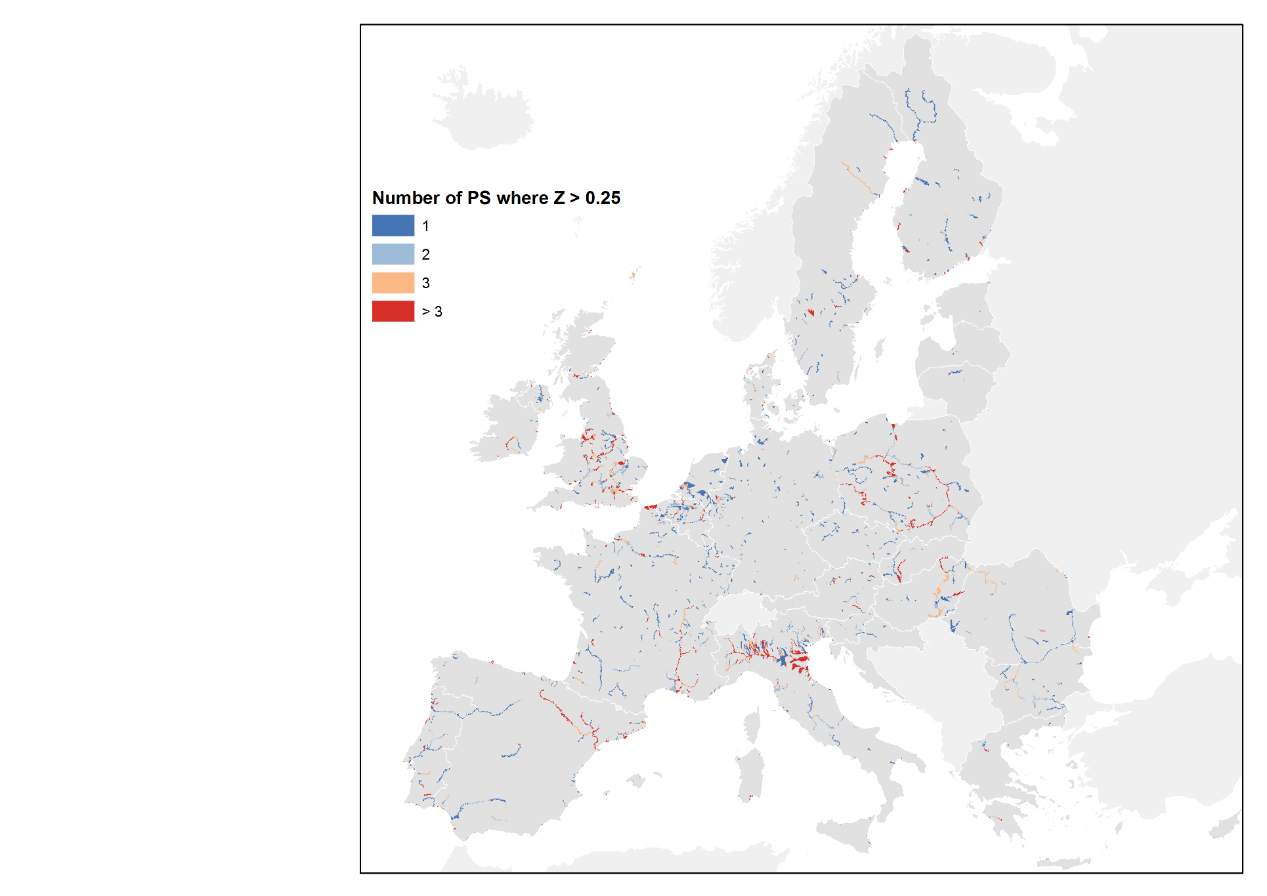
 b

Figure 4 SI – (a) Indicator Z, Maximum among the 29 substances of the ratio of concentration due to point sources on concentration due to total (point to diffuse) sources. (b) Indicator W, Number of priority substances with a point source contribution to concentration above 25% of total.

Indicator Z may be very high in the proximity of emissions, but tends to decrease significantly further downstream; moreover, usually high contributions in each river stretch correspond to one or two substances (W ≤ 2), and only in a few cases to more substances simultaneously.

The frequency distribution of Z in sub-basins with at least one sample for one of the 29 substances shows that the contribution of point sources to river loads (hence concentrations) is usually below 10%, with only a few percent of the samples subject to a potential contribution above 90% (representing a factor of about 2 in loads and concentrations). In addition, for individual substances the contribution of point sources at sampling sub-basins is by construction not larger, and generally rather lower than indicator Z.


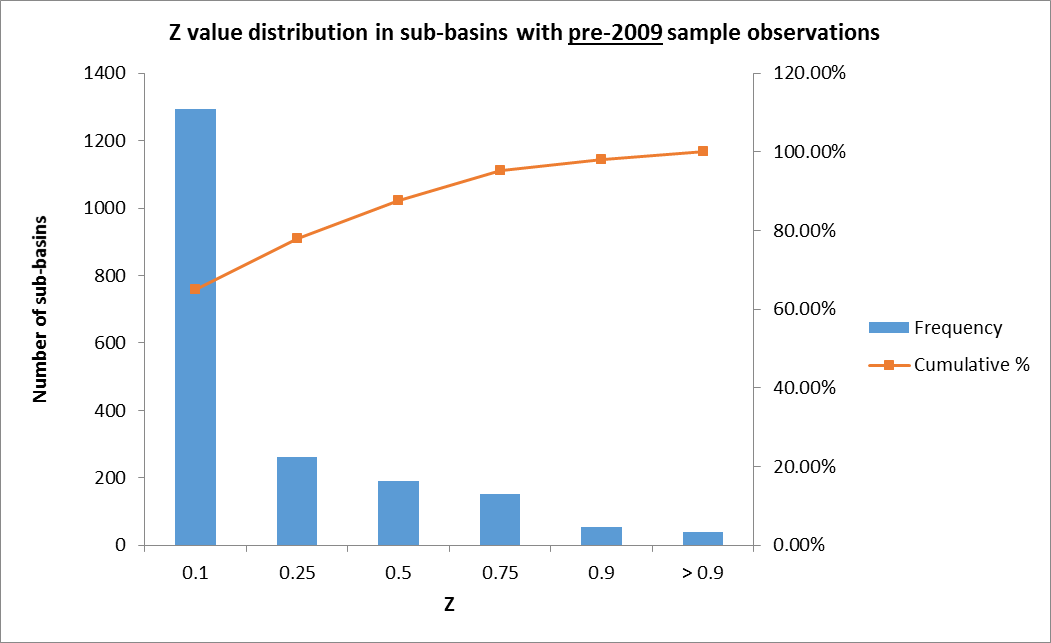


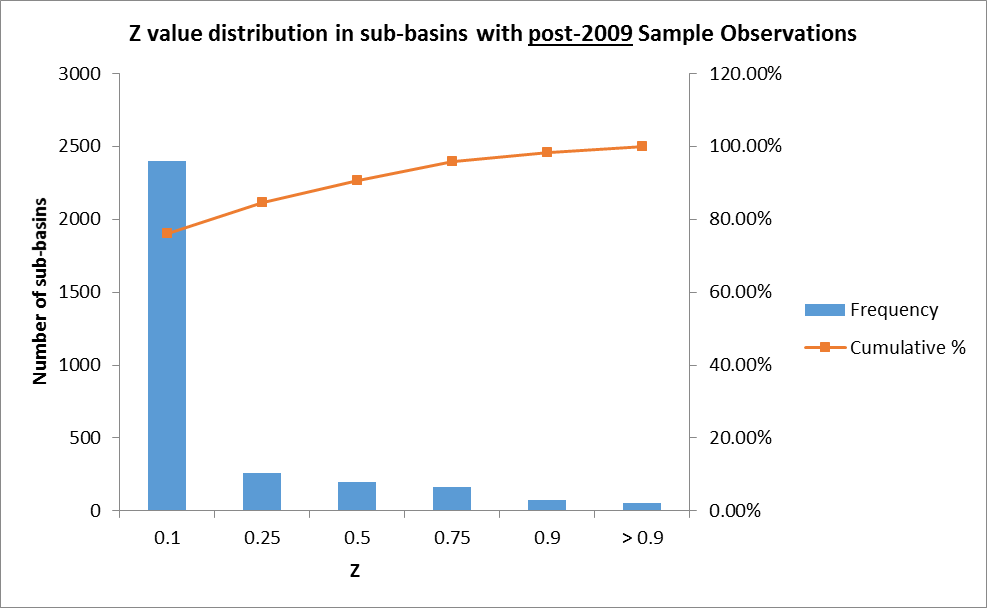


Figure 5 SI– distribution of Z (Figure 4 SI) for the sub-basins with at least one sampling station


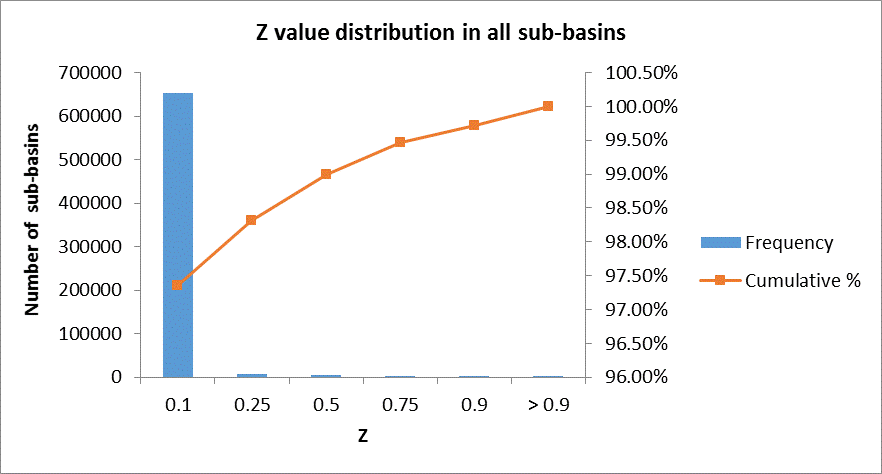


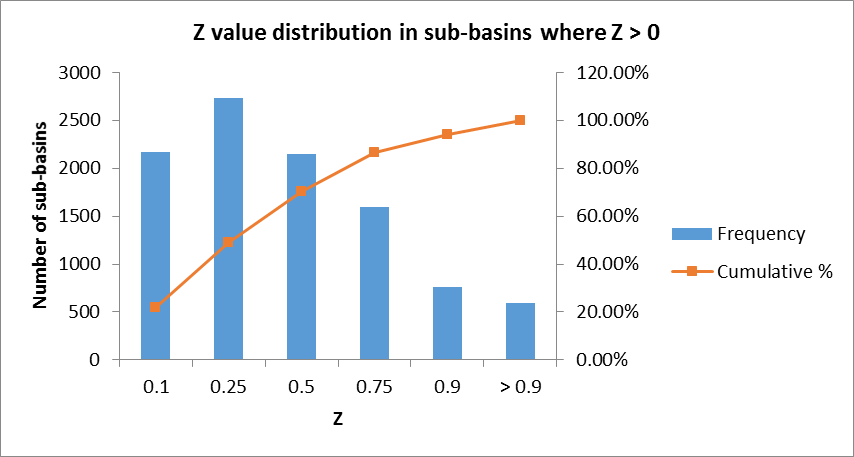


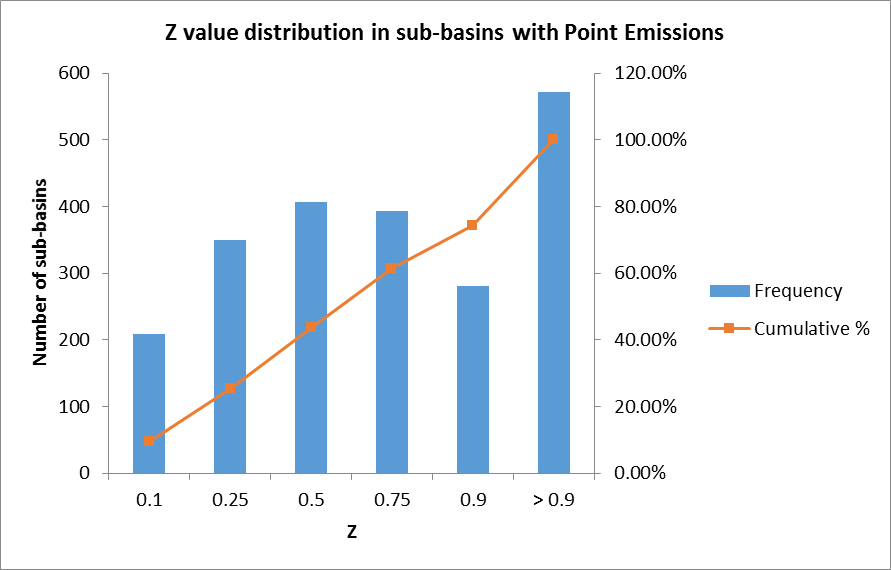


Figure 6 SI– distribution of Z (Figure 4 SI)


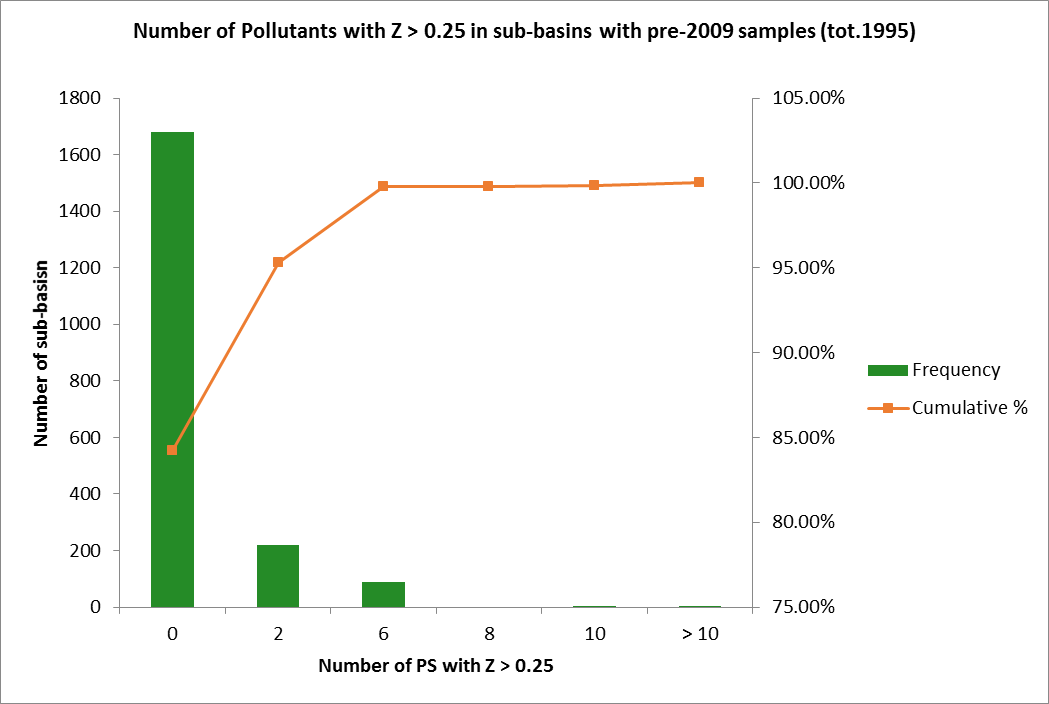


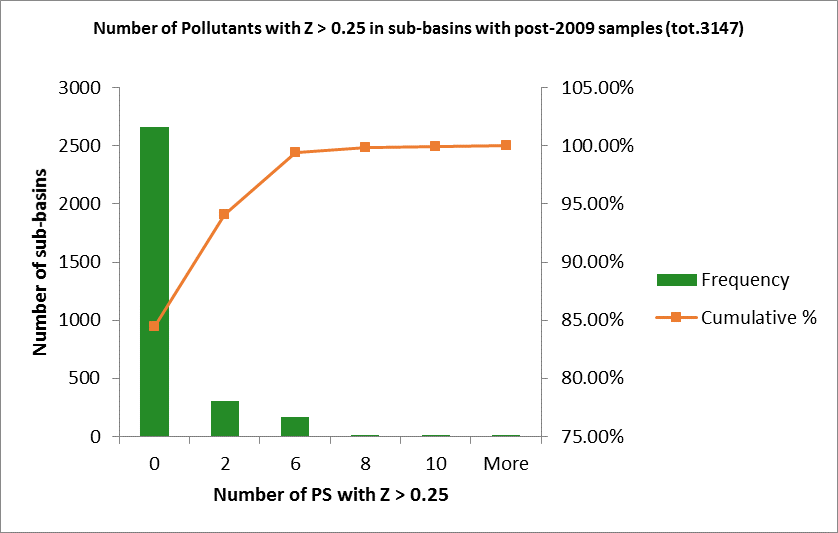


Figure 7 SI– distribution of W (Figure 4 SI) for the sub-basins with at least one sampling station


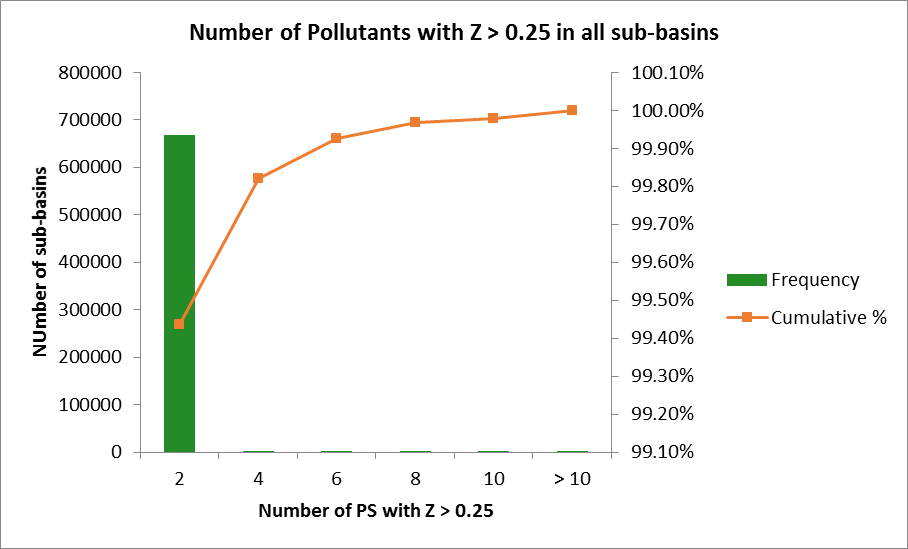


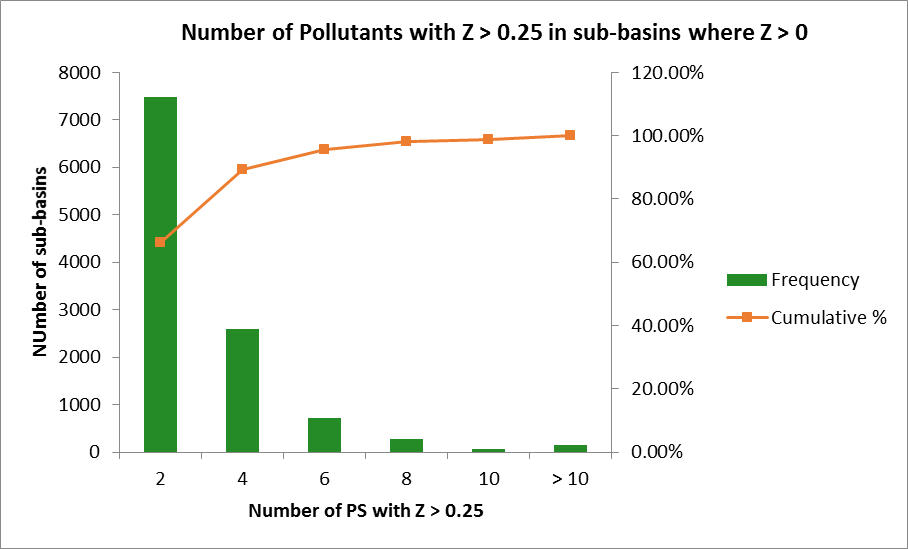


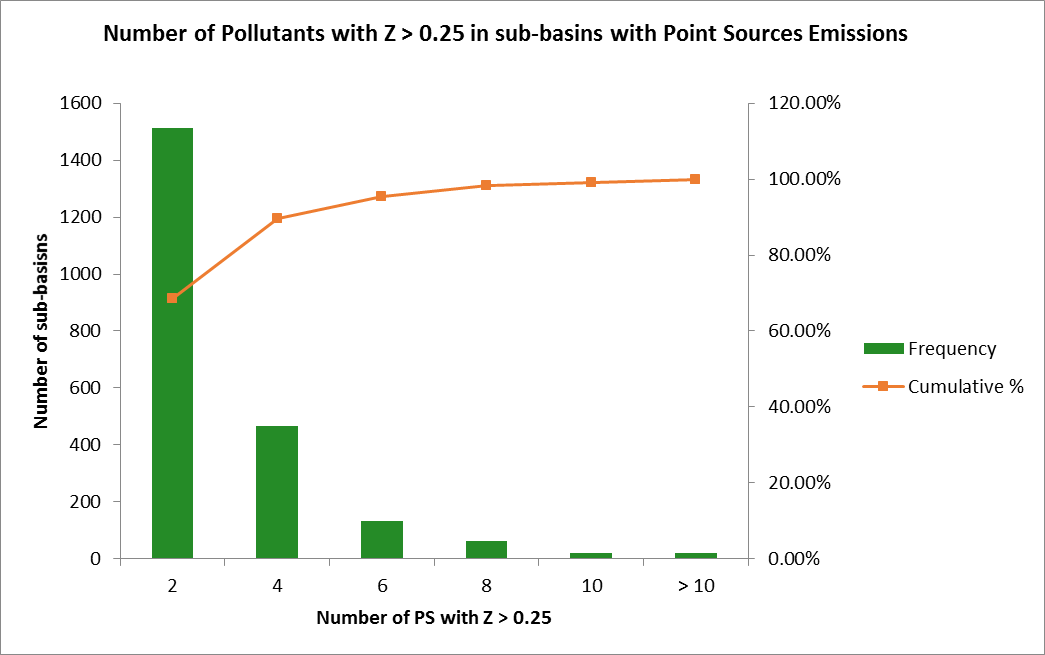


Figure 8 SI– distribution of W (Figure 4 SI)

## Point Emissions maps

| The figures below show the ratio of point emissions to diffuse emissions by river basin district across the EU, for the 29 substances covered by E-PRTR. | 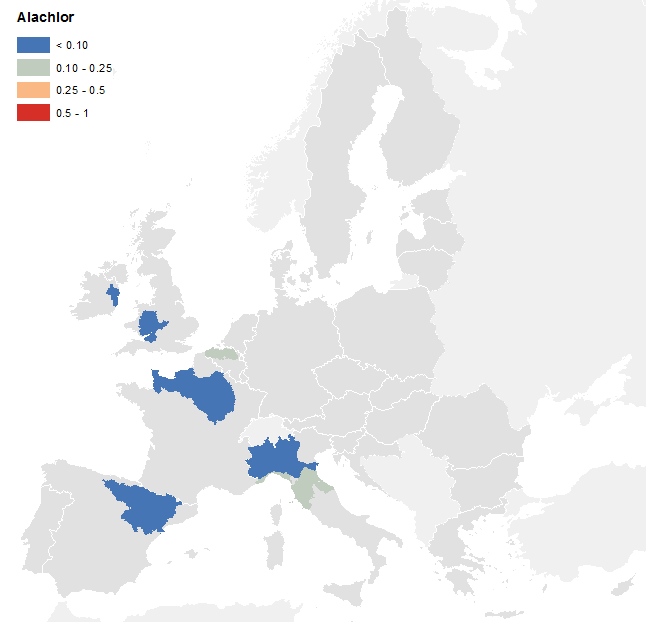 |
| --- | --- |
| 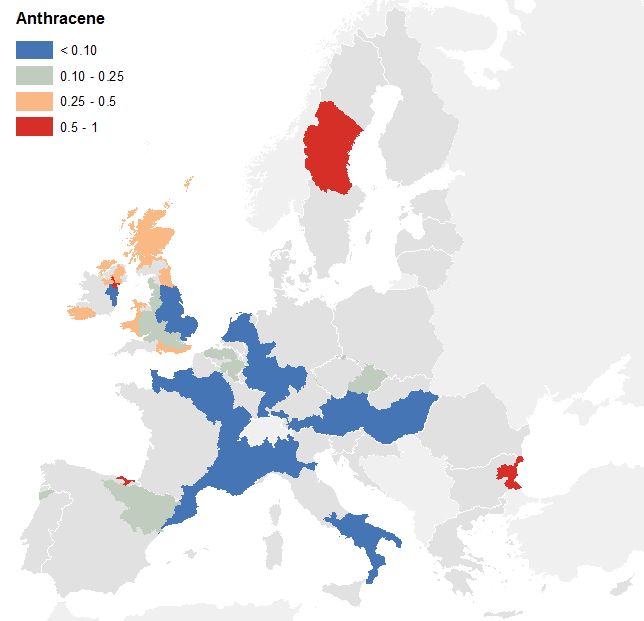 | 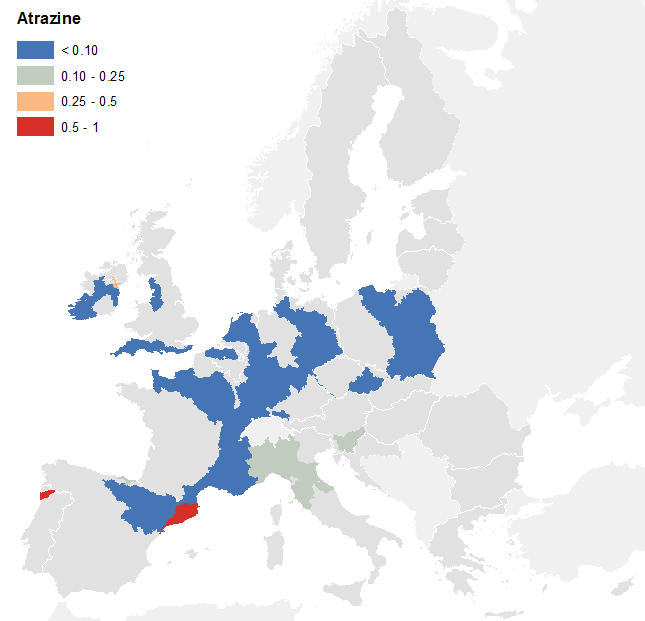 |
| 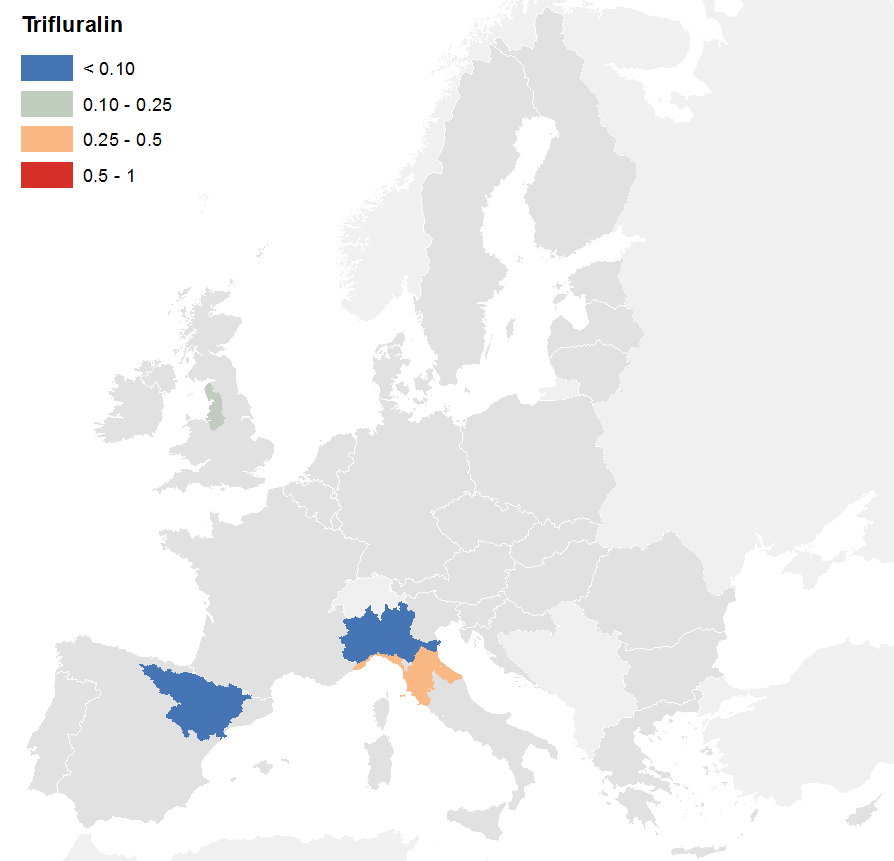 | 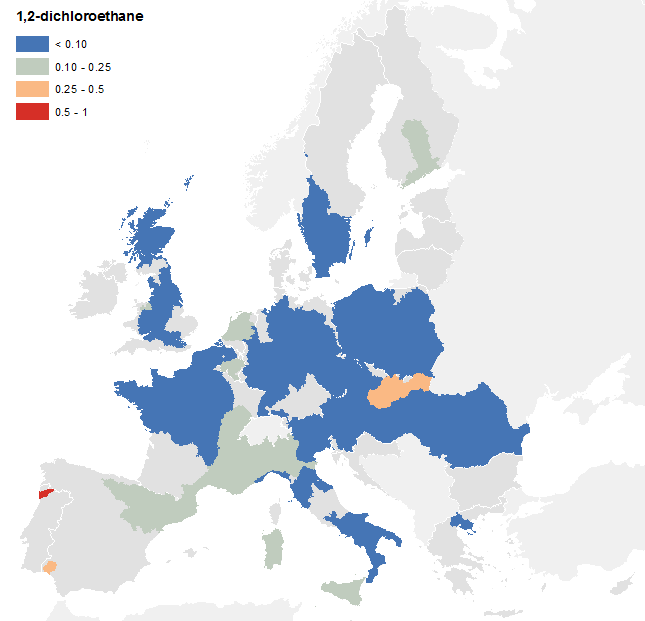 |
| 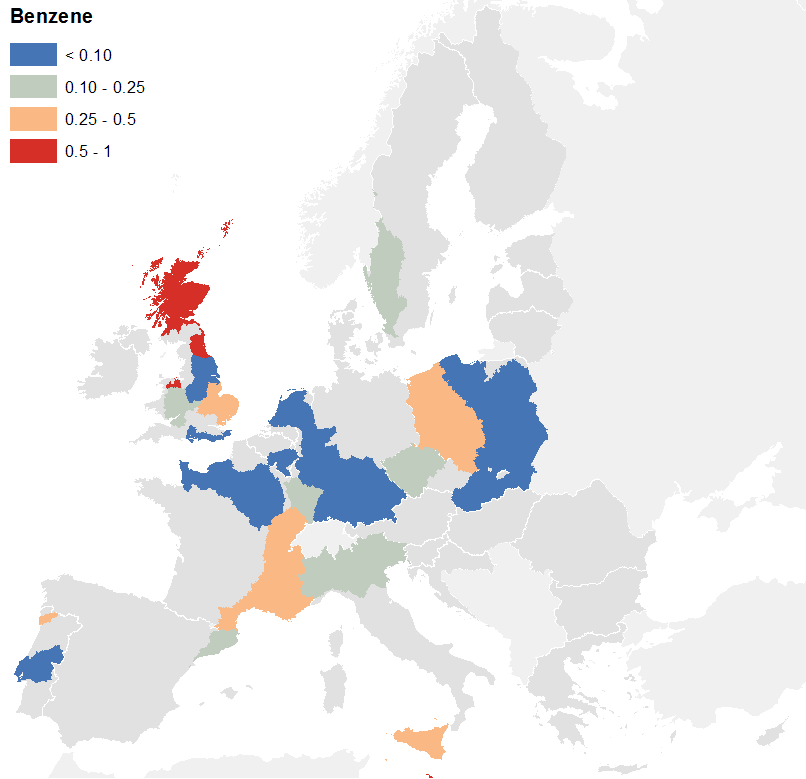 | 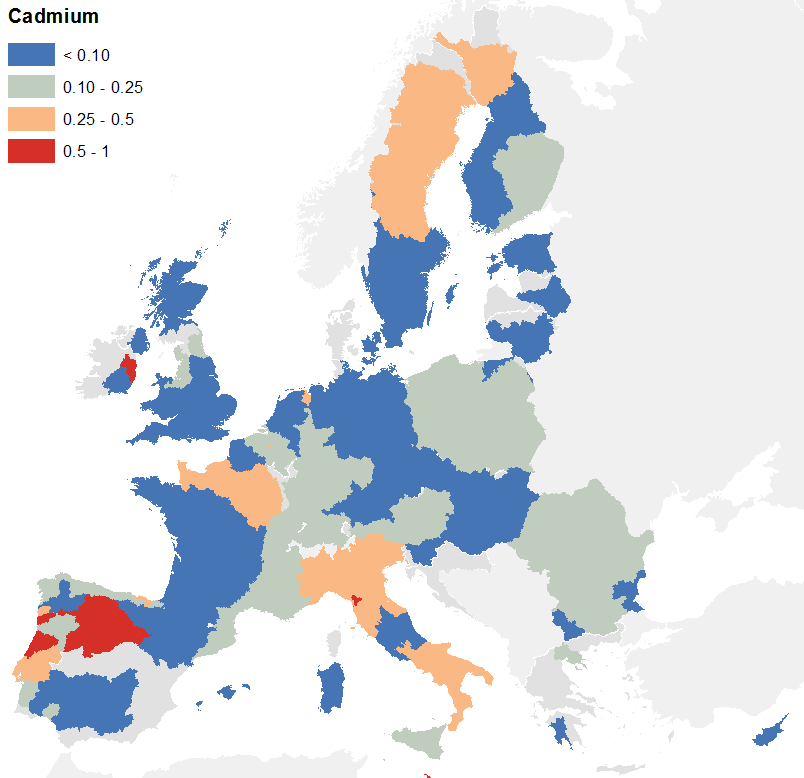 |
| 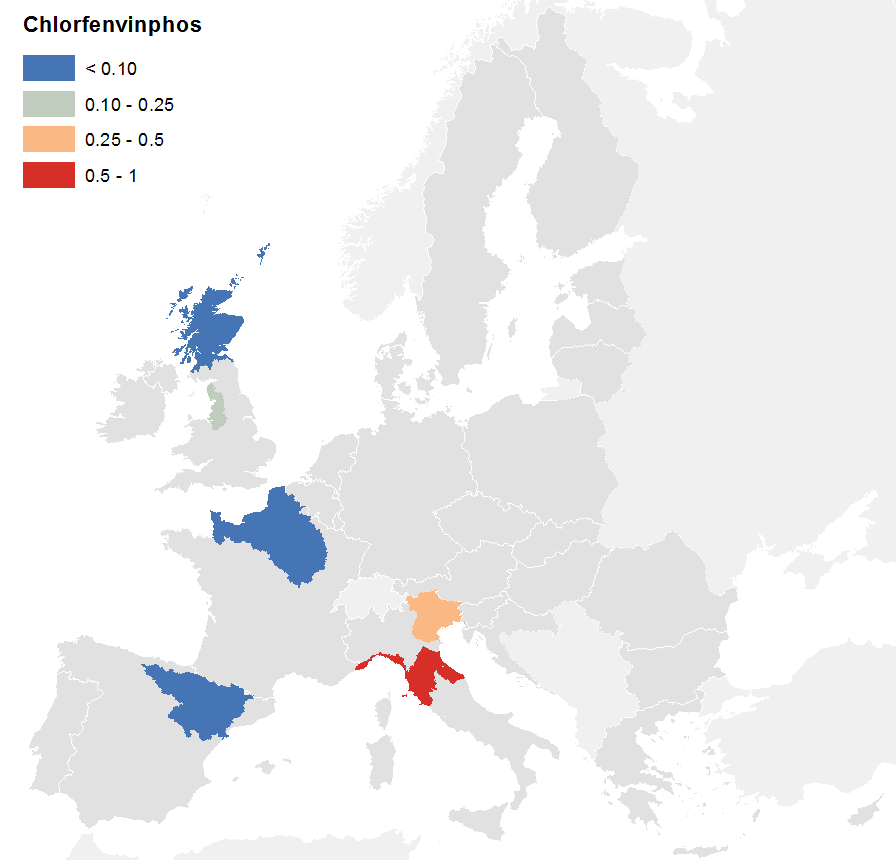 | 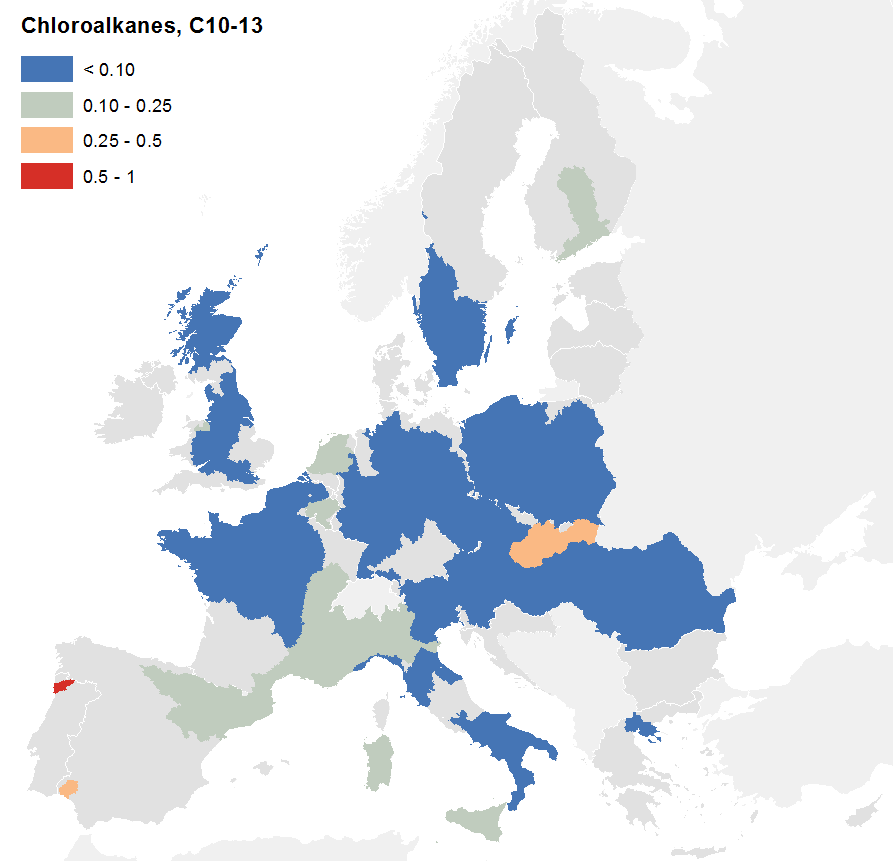 |
| 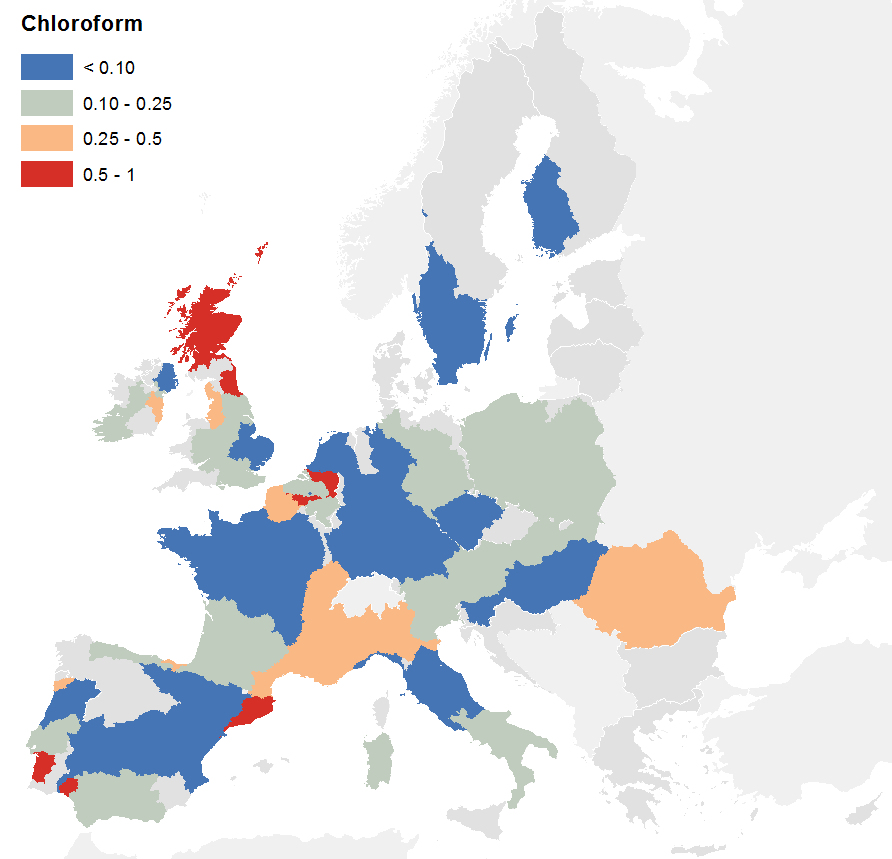 | 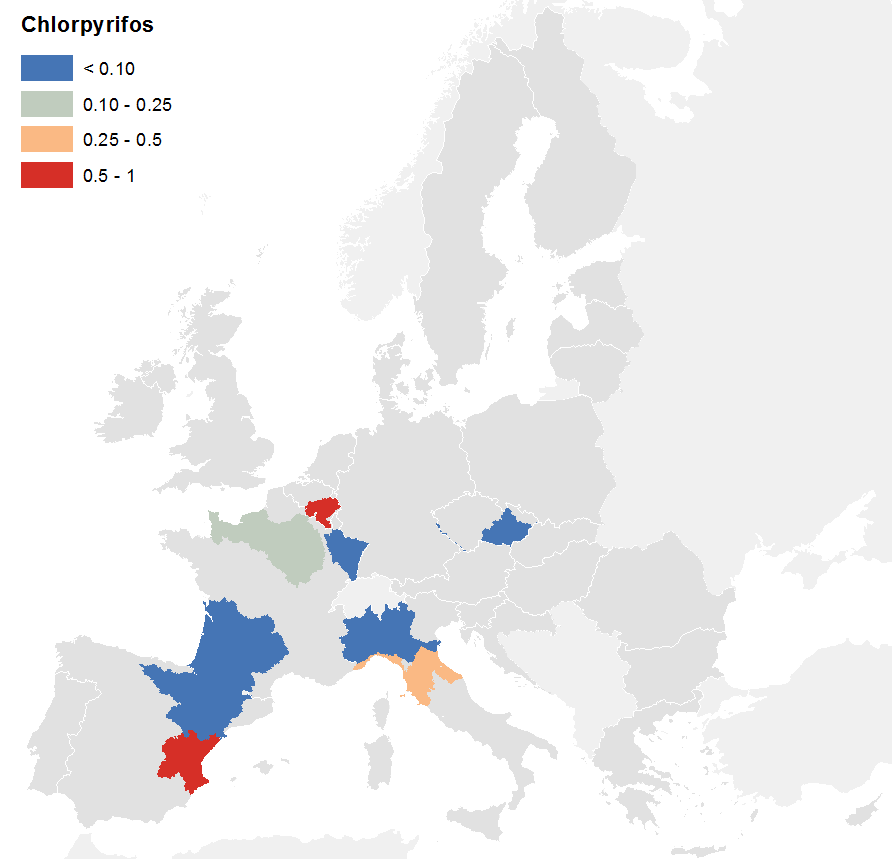 |
| 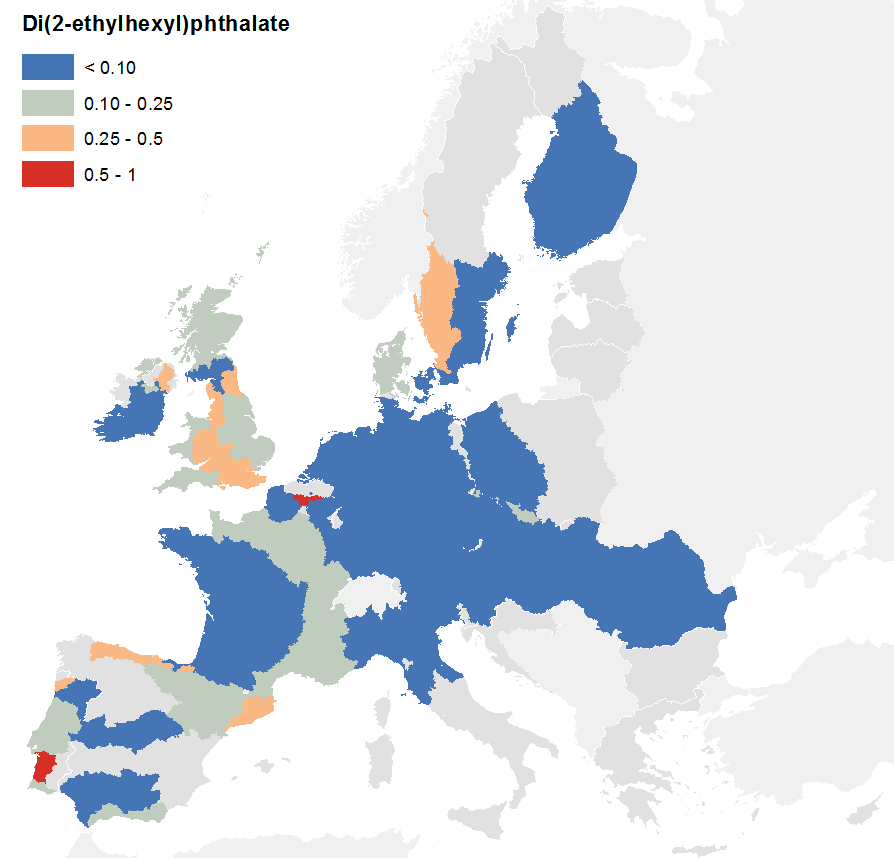 | 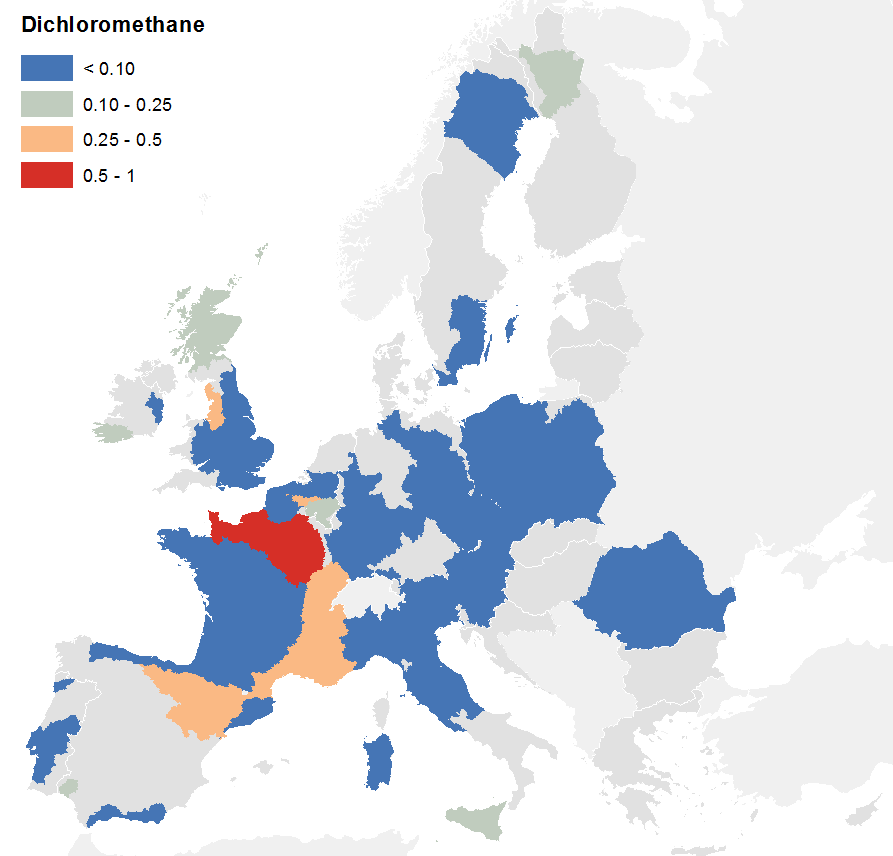 |
| 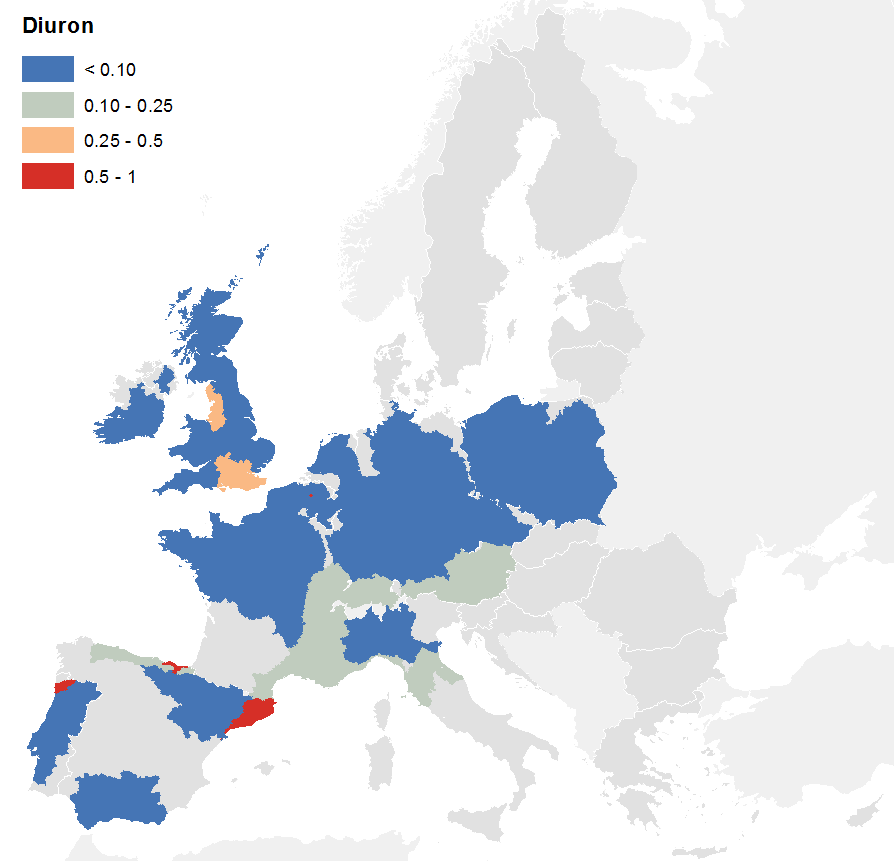 | 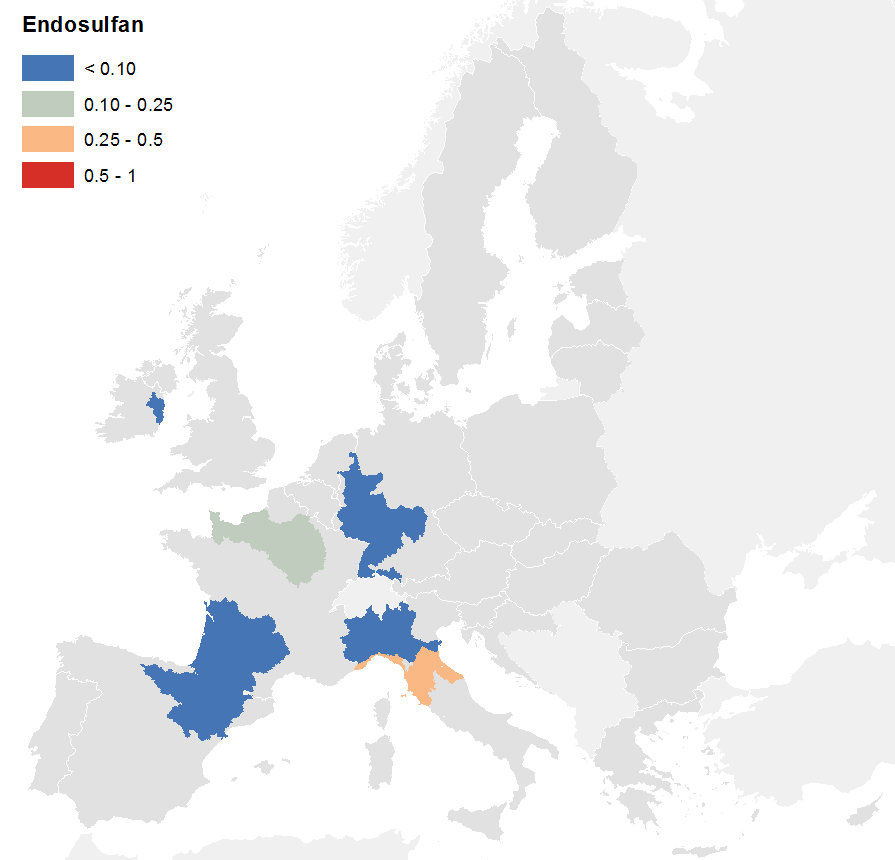 |
| 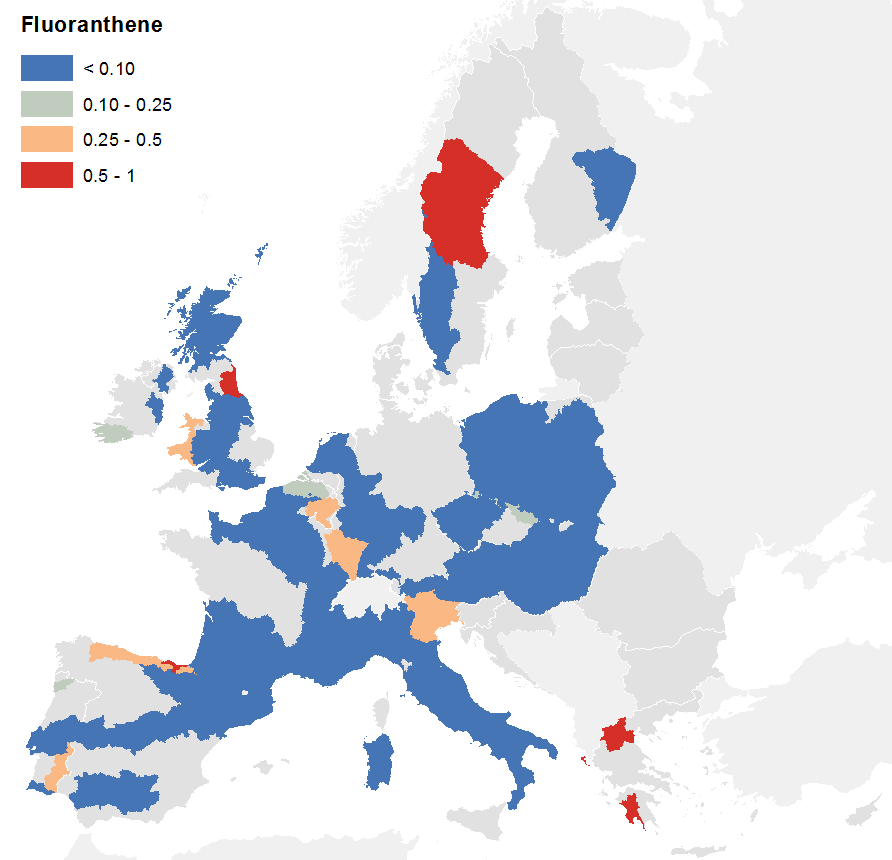 | 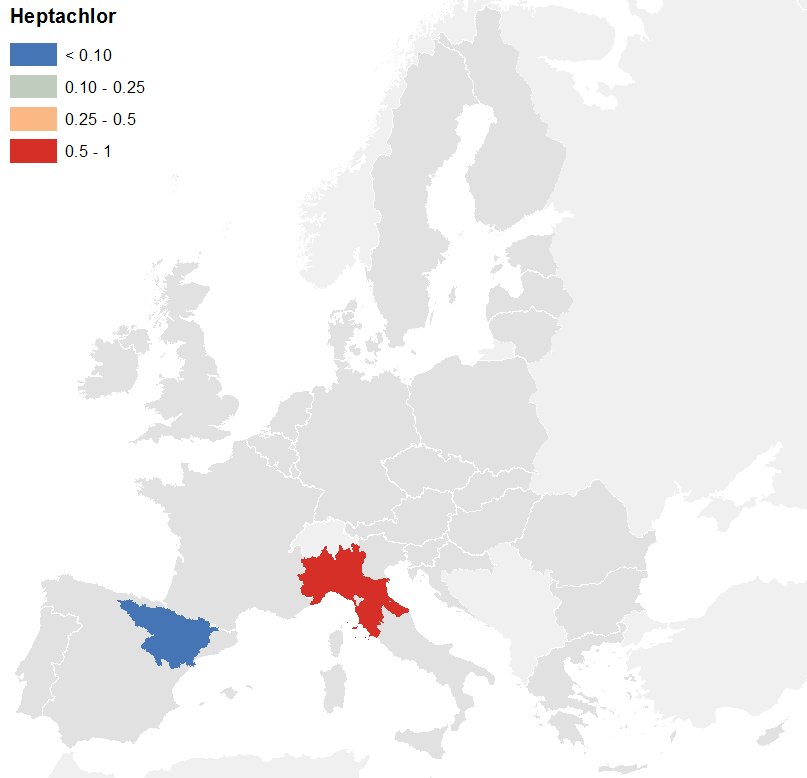 |
| 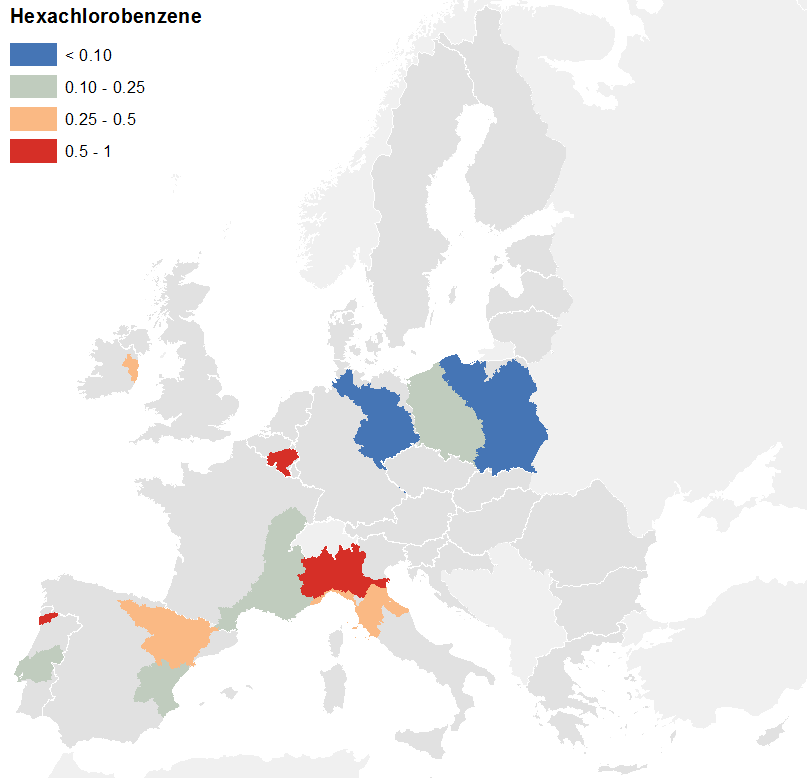 | 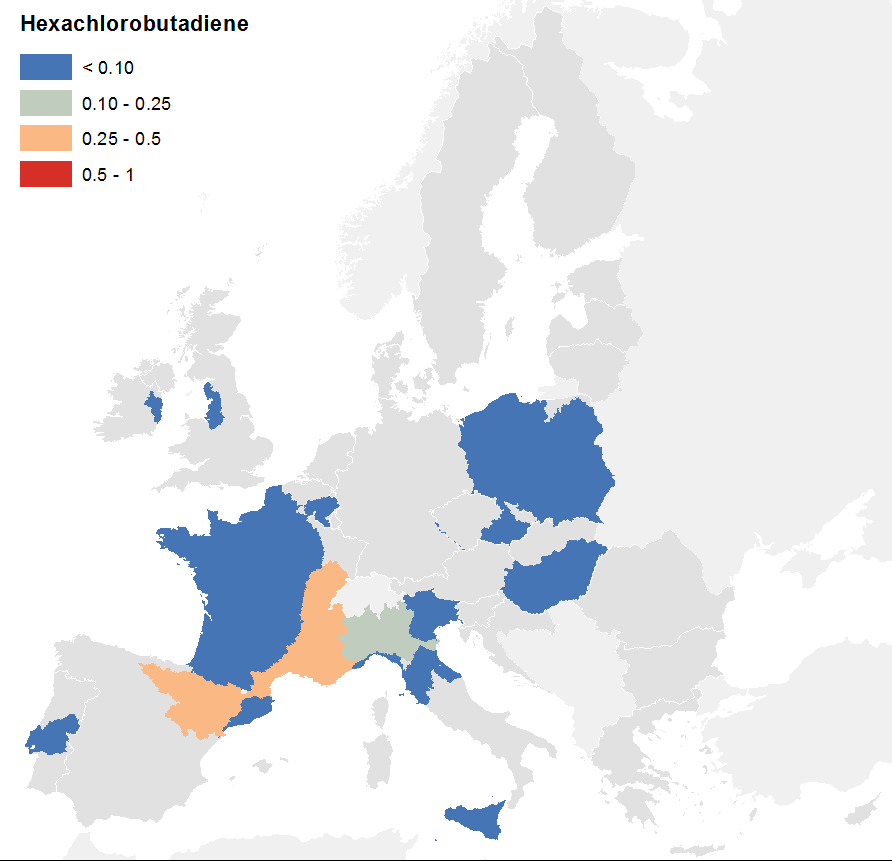 |
| 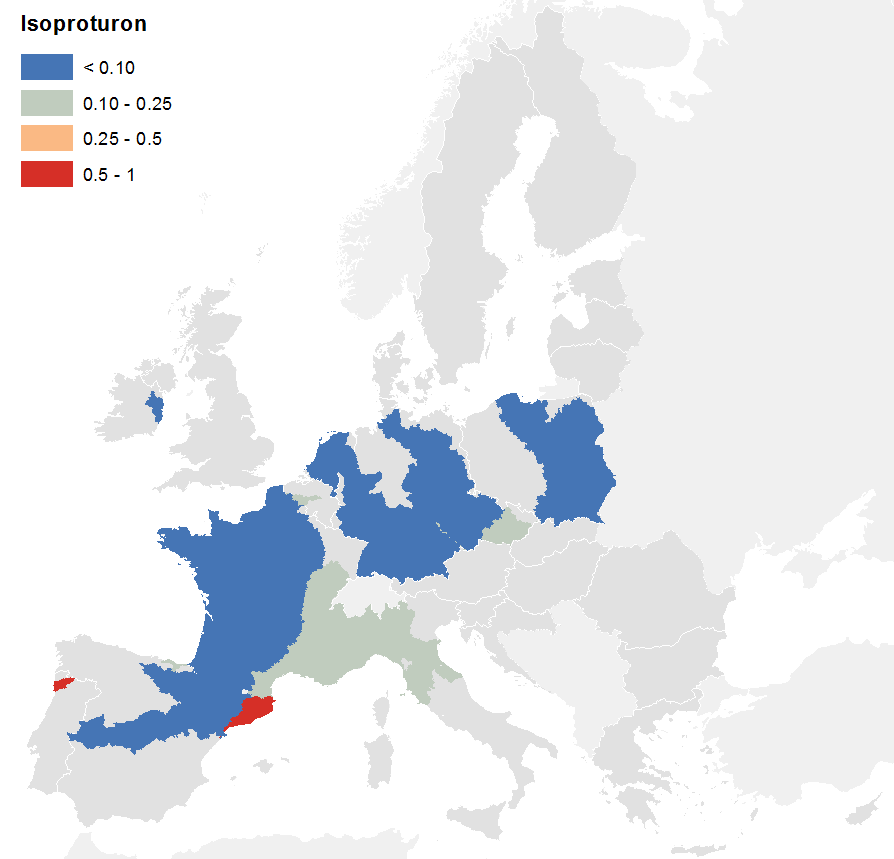 | 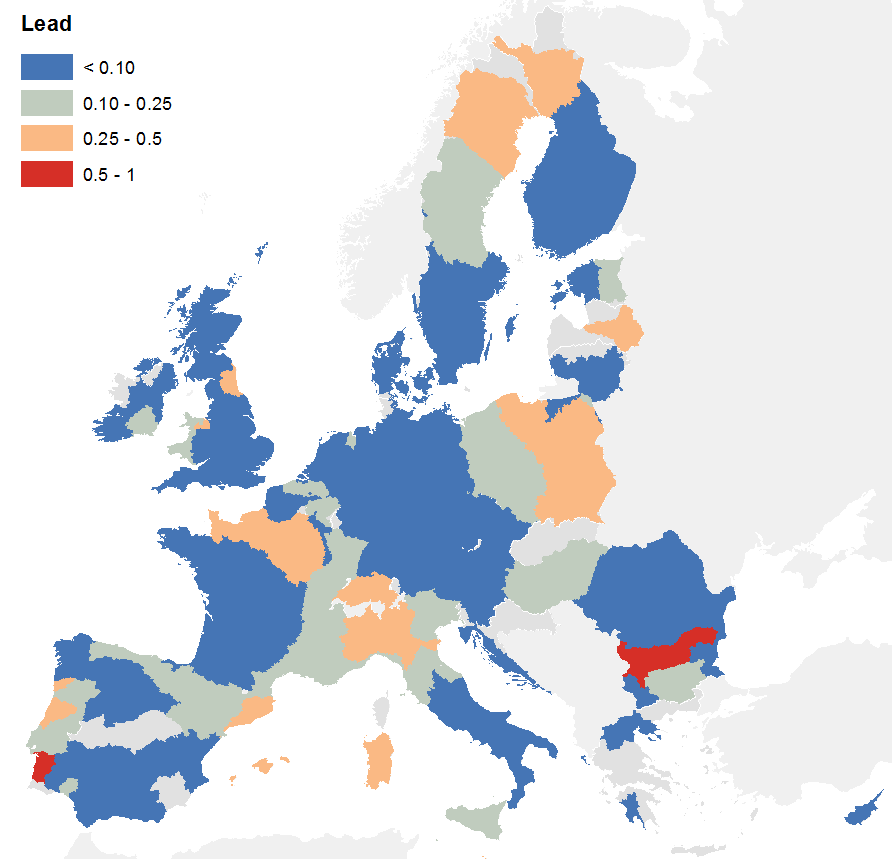 |
| 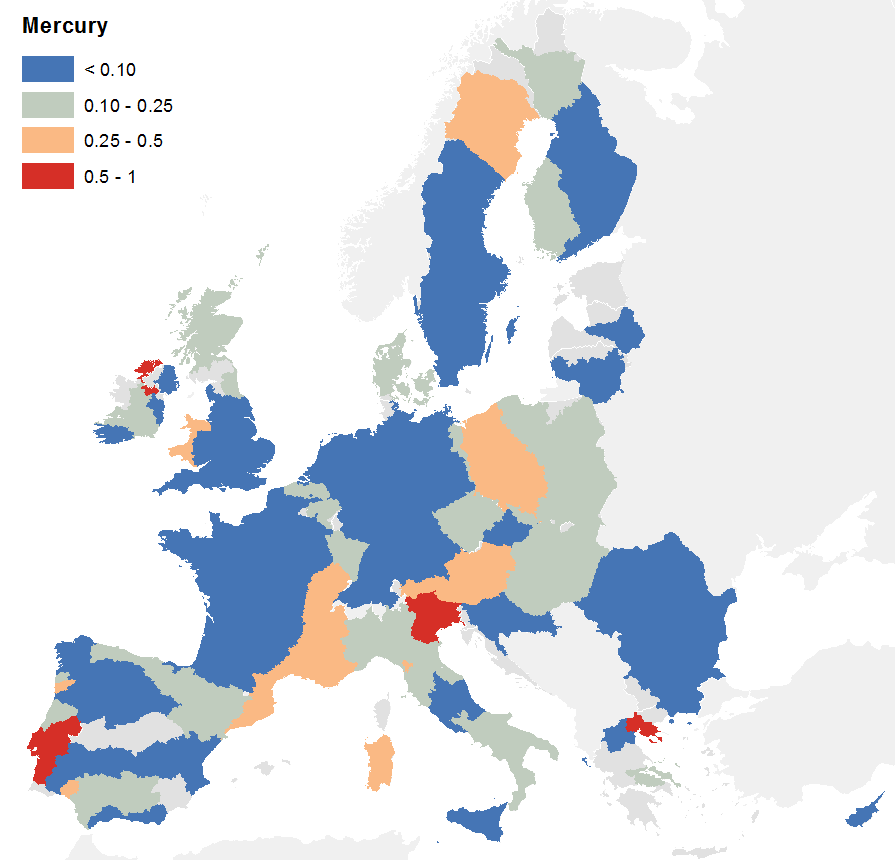 | 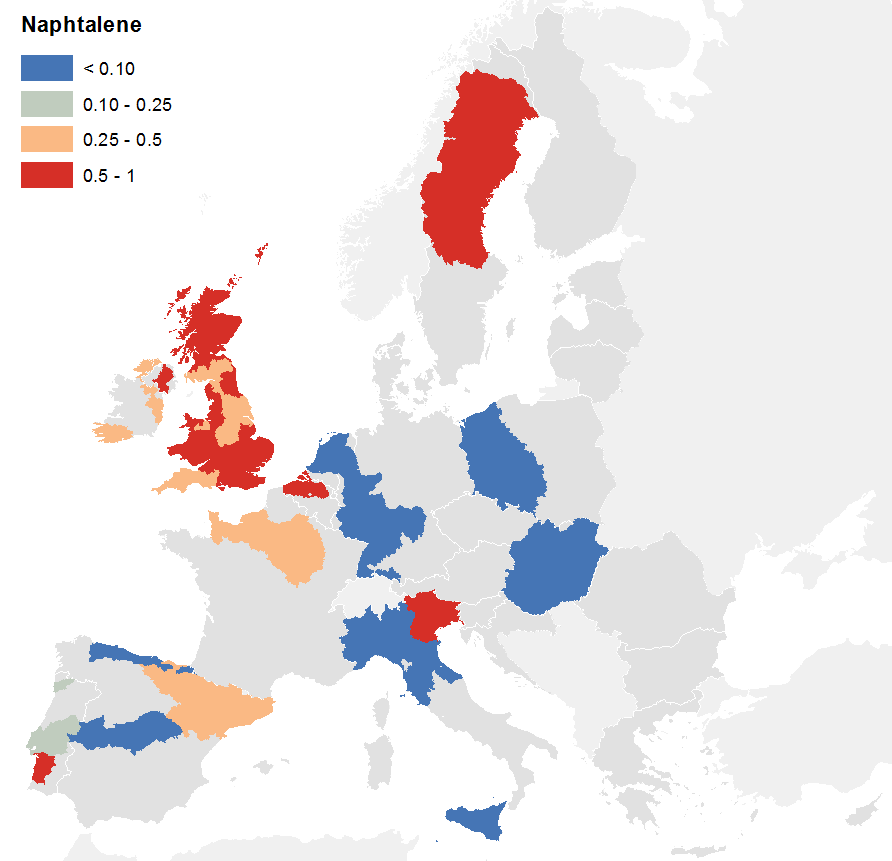 |
| 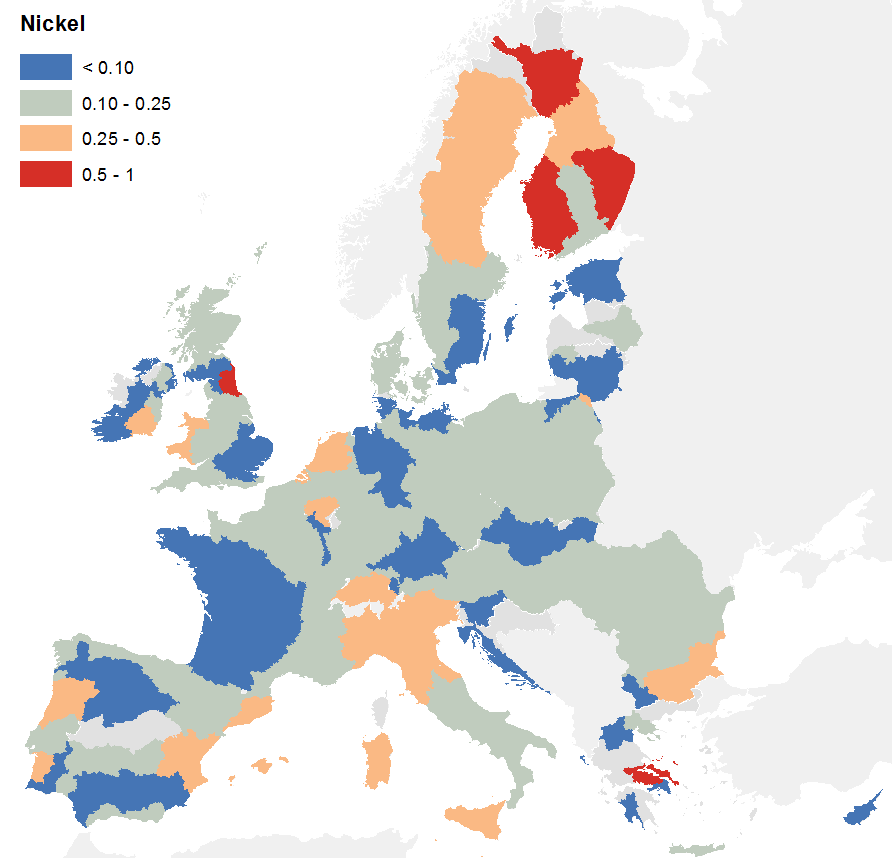 | 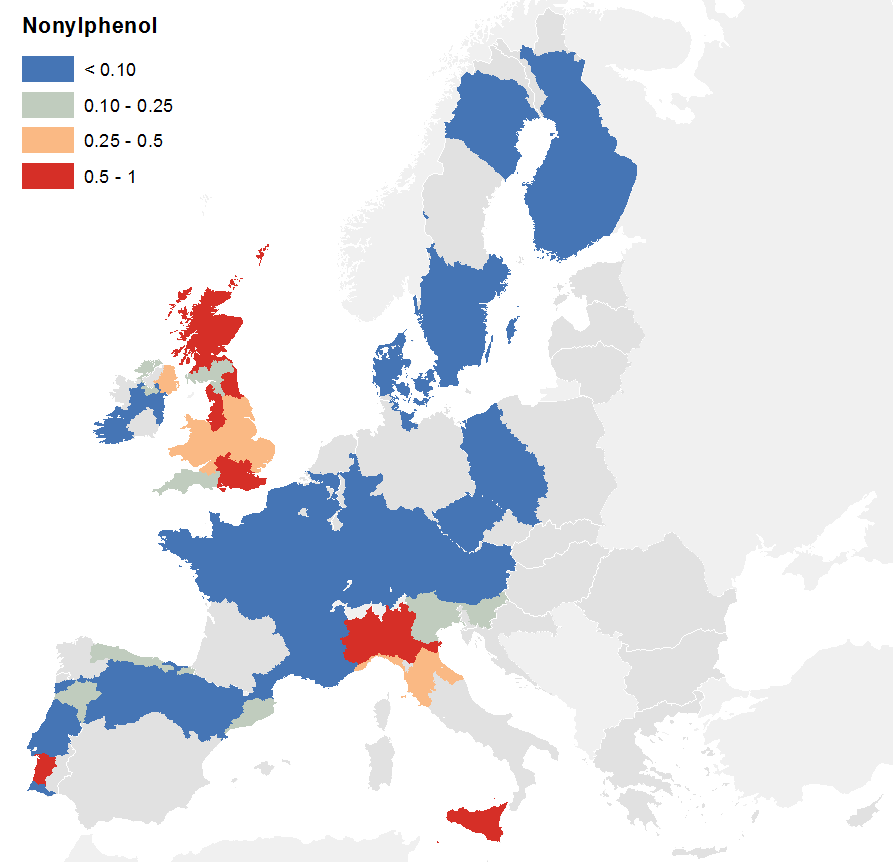 |
| 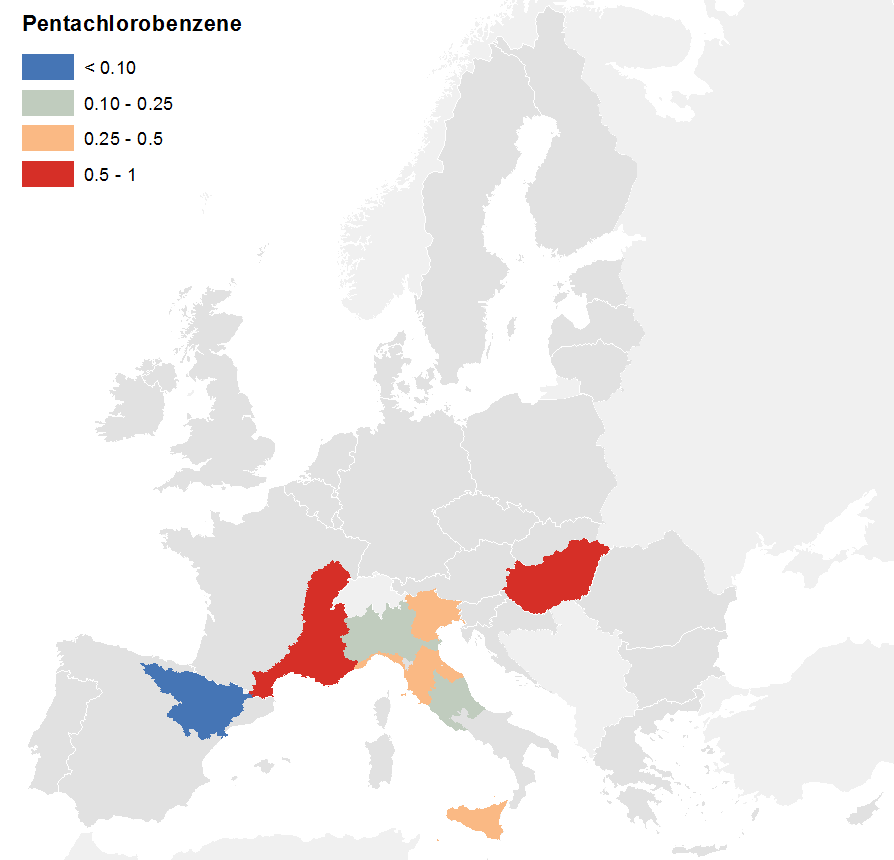 | 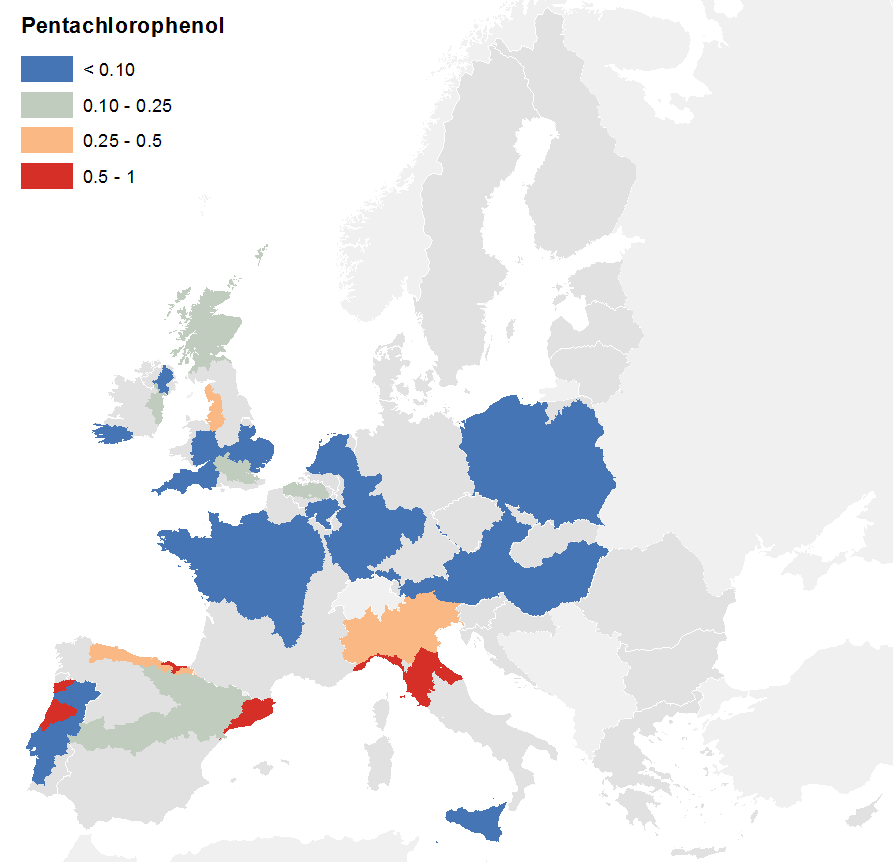 |
| 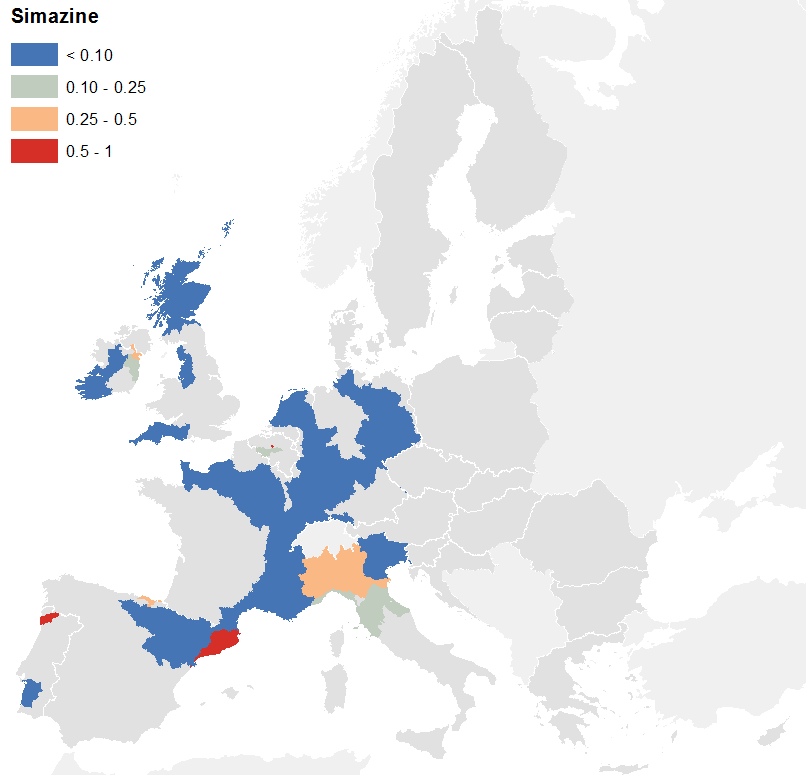 | 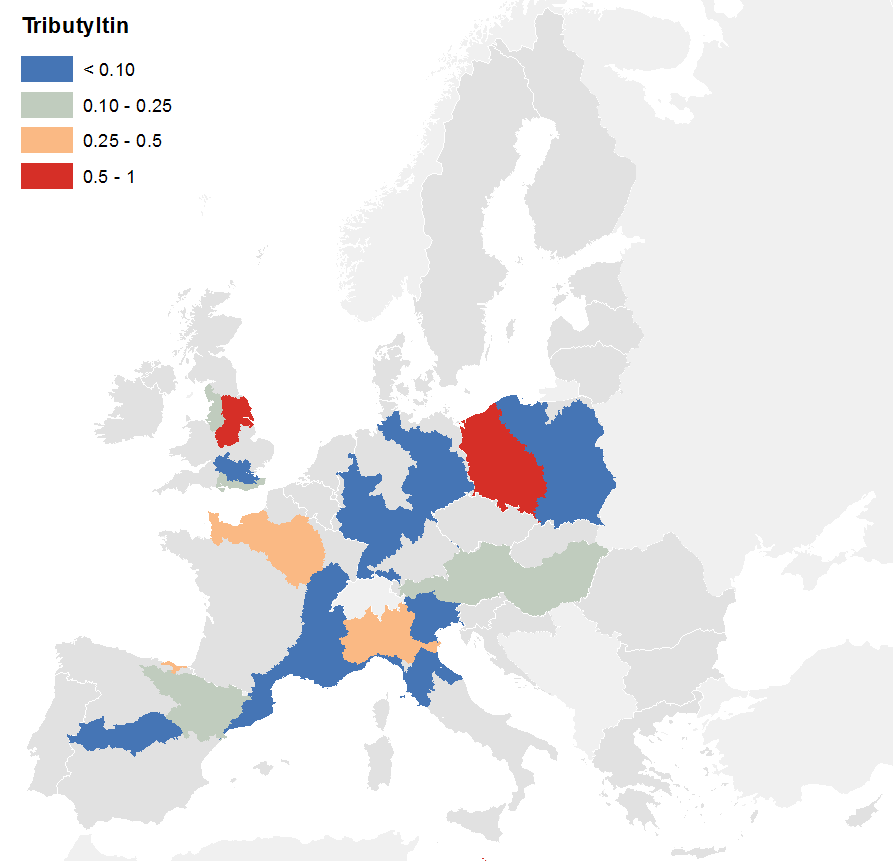 |

## Comparison with national inventories^[[4]](#footnote-4)^

A comparison has been drawn between our emission estimates and those produced by two EU Member States’ competent authorities, namely the Po River Basin District Authority (Italy)^[[5]](#footnote-5)^ and Northern Ireland’s Neagh Bann, North Eastern and North Western river basins (UK)^[[6]](#footnote-6)^. The comparison (Figure 9 SI) highlights that estimates lay within one order of magnitude of each other, with a few exceptions. Emissions reported for the Po river basin district include two substances (Nickel and Lead) evaluated within the river basin management plan (RBMP) at a few river cross sections, and other substances for which emissions are reported to the competent authority by the regions within the river basin district. Reported emissions may include only point sources (which may explain the grossly higher value computed with our approach for Anthracene, Hexachlorobutadiene and Mercury in the Po) or may reflect specific situations, arguably compatible with deviations from the factor-10 consistency found in the majority of the cases.

The Netherlands maintain a national emission inventory^[[7]](#footnote-7)^, reporting inter alia emissions of priority substances. A comparison between the emissions estimated here and those of the Dutch register (Figure 10 SI) highlight generally a good agreement (within one order of magnitude) except for certain substances for which reported emissions are substantially lower than our estimates (Chloroalkanes C10-13, Bifenox and, to a lesser extent, Trifluralin, Simazine, Diuron, Hexachlorobutadiene, 1,2-Dichloroethane and Dichloromethane). The discrepancy may be simply due to the fact that the Netherlands, for these specific chemicals, have emissions below the EU average, in principle reflected in our emission factors.

Many EU Member States report emissions of priority substance in one or both of the following reporting schemes:

1. On a voluntary basis to the European Environment Agency in the scope of the State of Environment reporting, under the Water Information System for Europe (WISE)^[[8]](#footnote-8)^ ;
2. On a mandatory basis under the reporting guidance for the Water Framework Directive (WFD)’s second cycle of river basin management plans^[[9]](#footnote-9)^. These data are equally collected by the European Environment Agency and should in principle correspond to the requirements of Directive 2013/39/EU.

In addition, Member States report emissions under the E-PRTR for many of the priority substances. The data reported under the three abovementioned schemes (WISE, WFD and E-PRTR) generally suffer from incompleteness and several quality limitations^[[10]](#footnote-10)^, but represent anyway a reference for comparison to our estimates. In this study, it was possible to retrieve data on reported emissions for 11 substances of the 36 addressed, thanks to the contribution of the Blue2 study^4^. These are compared with our estimates of diffuse emissions in Figure 11 SI, Figure 12 SI and Figure 13 SI. It can be seen that reported emissions are often significantly lower than our estimates, which confirms the questionability of reporting completeness. The discrepancy is further exacerbated if one adds to our diffuse emissions those reported in the E-PRTR.

Substances for which E-PRTR provides diffuse emission estimates, as well as the inventory of point emissions, the match with our estimates is significantly better (this is the case of metals, anthracene and fluoranthene). Pesticides (hexachlorocyclohexane (HCH) and Isoproturon) are the substances with most apparent mismatch. Phtalate (DEHP), nonylphenols (NP) and tributyltin compounds (TBT) show an intermediate match (with TBT notably consistent in terms of orders of magnitude, although with no correlation between estimates and reported emissions). However, for all substances except pesticides there is a reasonable correspondence between reported and estimated values for a number of European Member states.


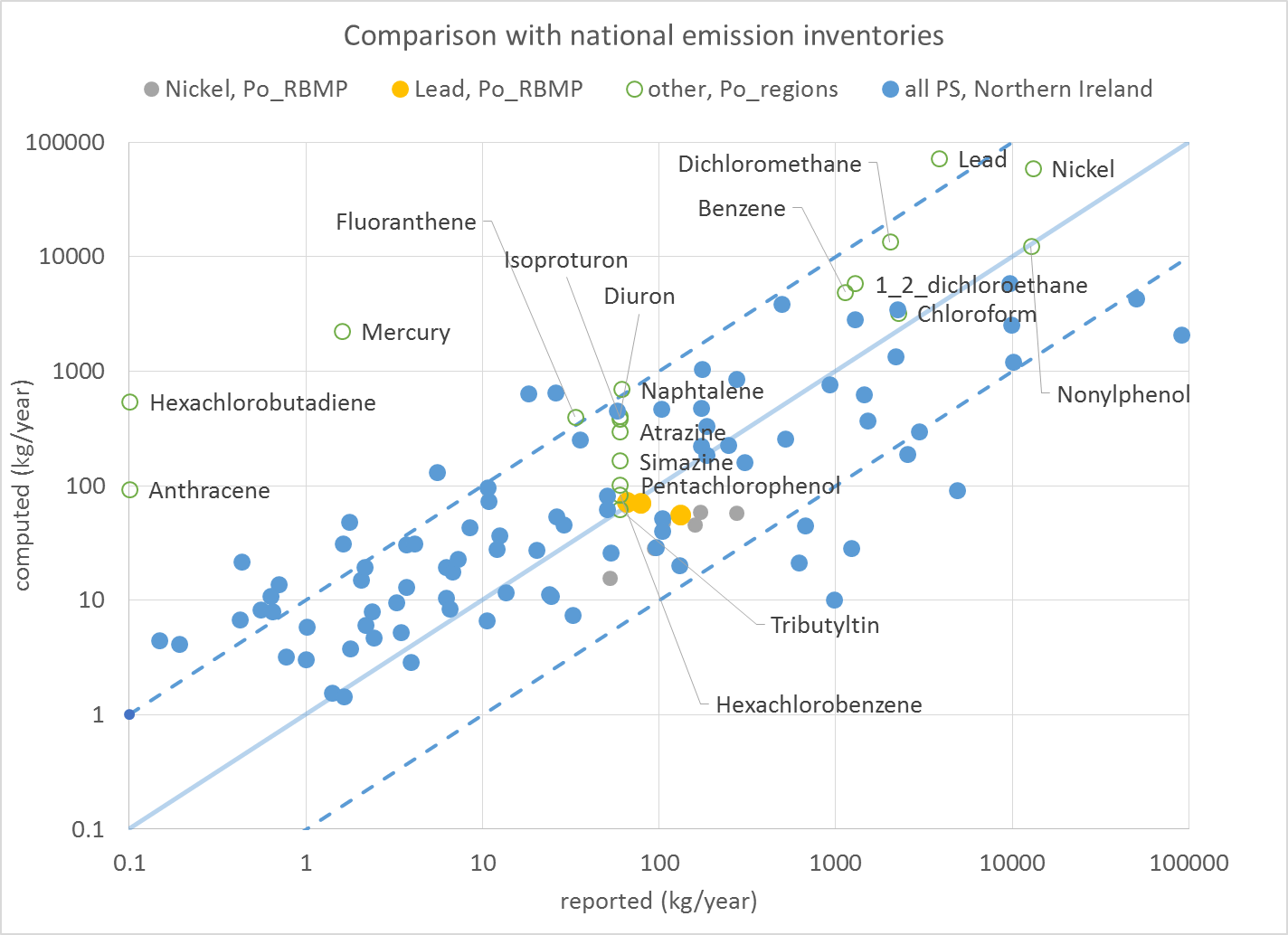


Figure 9 SI – comparison of reported and computed emissions for the Po and Northern Ireland’s river basins.


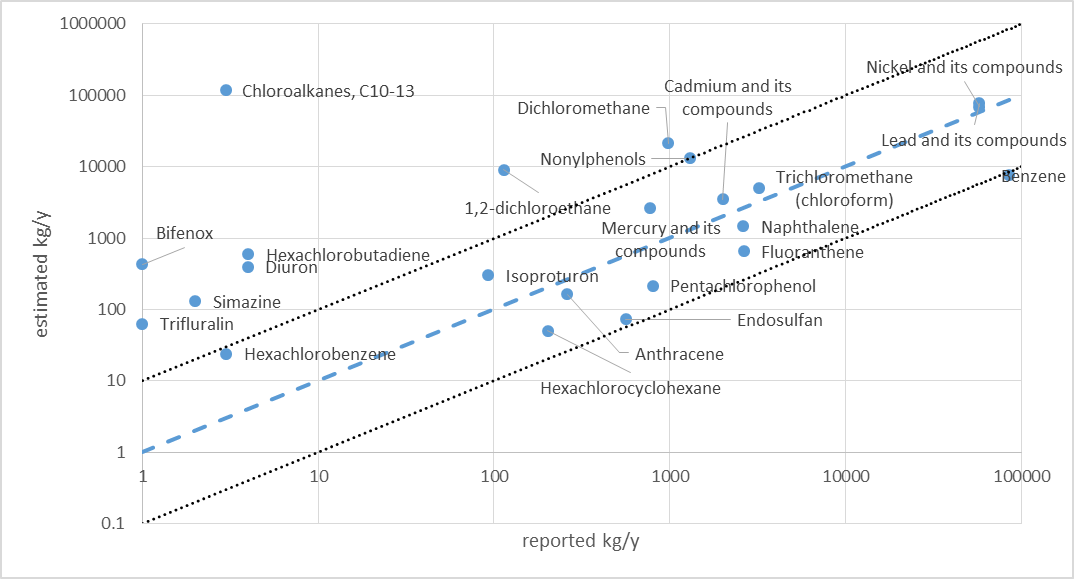


Figure 10 SI – comparison of reported and computed emissions for the Netherlands. Reported data in this graph refer to the year 2016, the most recent available.


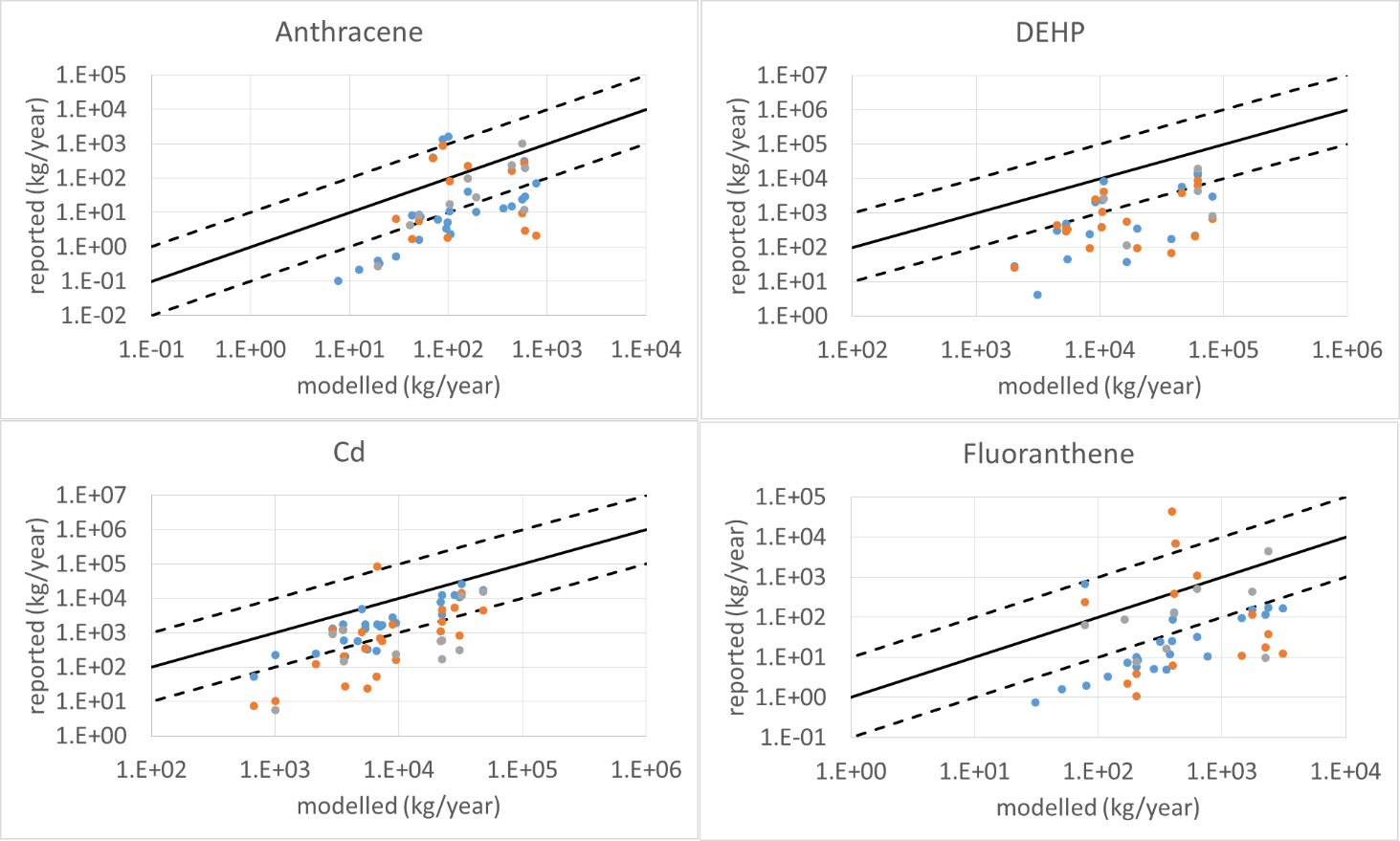


Figure 11 SI – scatter plot of reported country total emissions of selected priority substances, as a function of our estimated emissions for the EU Member States (color-coded as: WISE: Grey; WFD: Orange; E-PRTR: Blue).


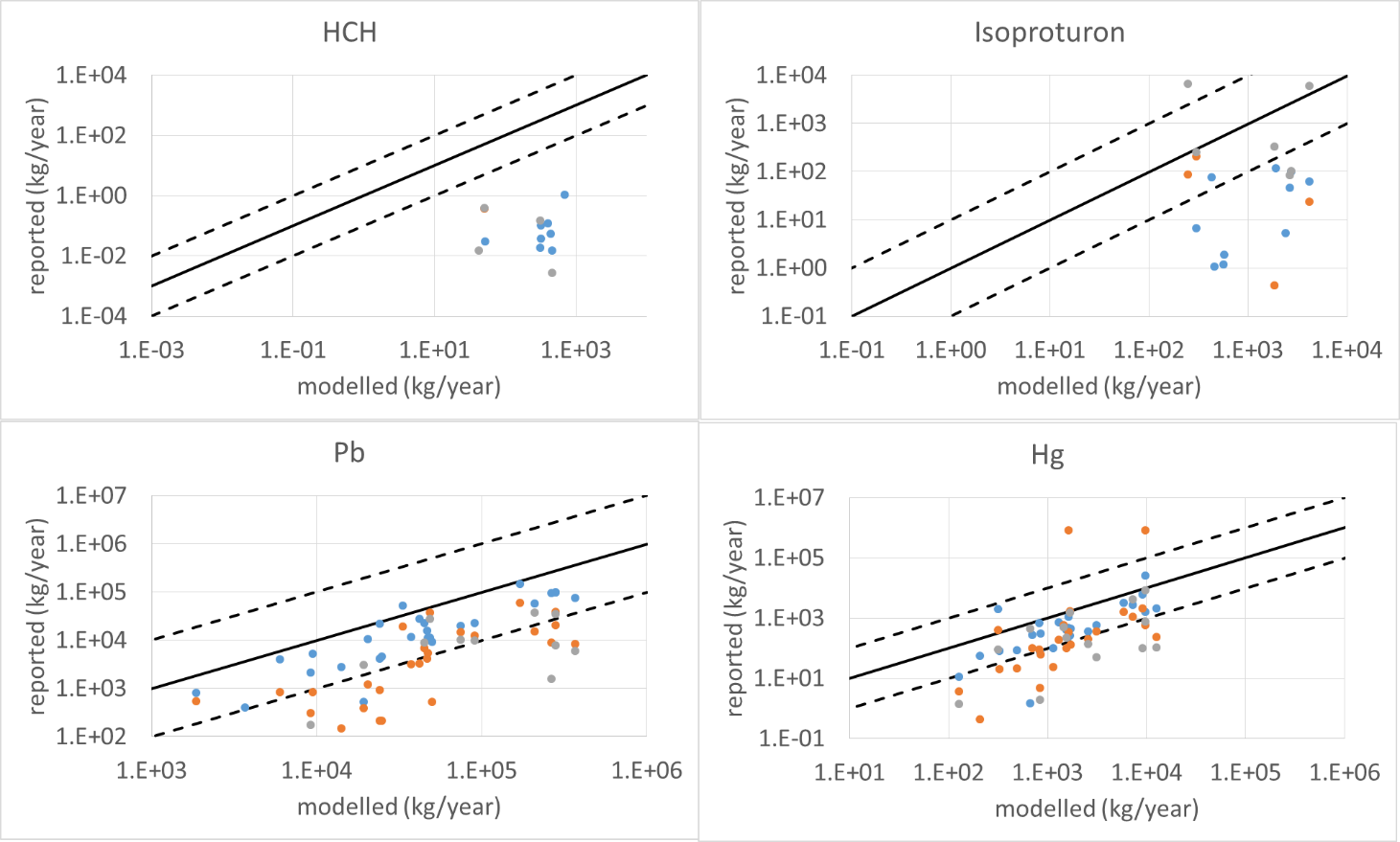


Figure 12 SI – scatter plot of reported country total emissions of selected priority substances, as a function of our estimated emissions for the EU Member States (color-coded as: WISE: Grey; WFD: Orange; E-PRTR: Blue).


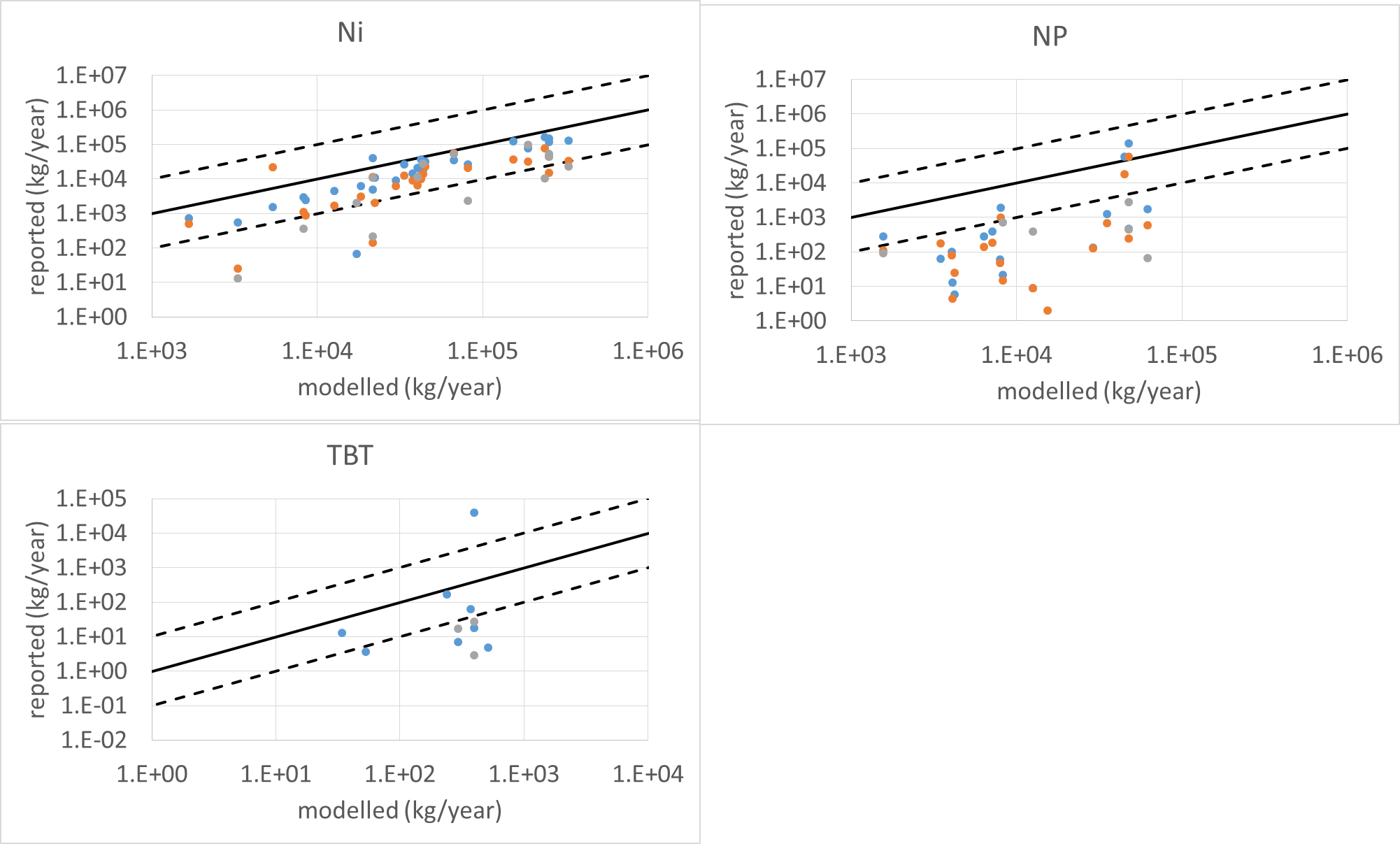


Figure 13 SI – scatter plot of reported country total emissions of selected priority substances, as a function of our estimated emissions for the EU Member States (color-coded as: WISE: Grey; WFD: Orange; E-PRTR: Blue).


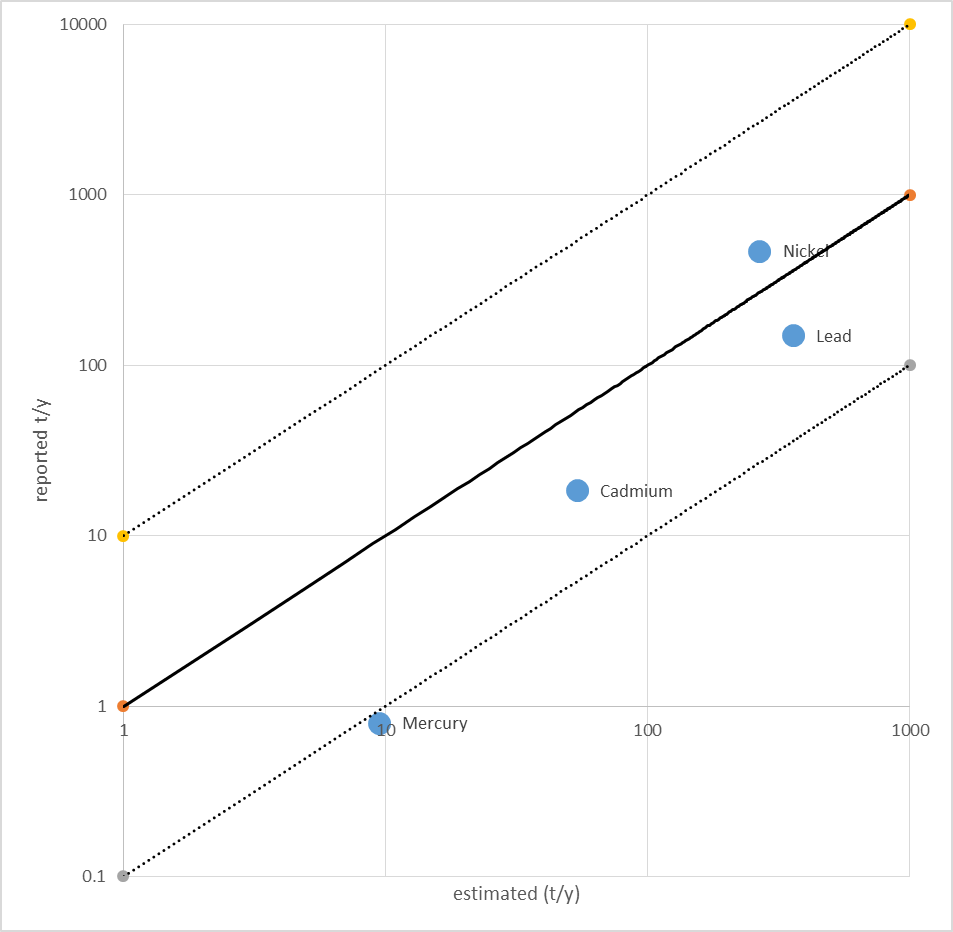


Figure 14 SI – comparison of loads to the Baltic sea estimated in this research, with those reported by HELCOM PLC5

When considering loads to the seas, the Fifth Pollutant load compilation (PLC5) to the Baltic sea prepared by HELCOM in 2011^[[11]](#footnote-11)^ reports inter alia loads for metals (Cadmium, Nickel, Lead and Mercury which compare rather favourably with our estimates (Table 3 SI), as shown in Figure 9 SI.

The above comparisons do not enable a validation of our estimates of emissions; however, they show that inverse modelling of emission factors assuming emissions to follow simple emission patterns yields total emissions reasonably close to existing estimates, and for those cases (such as the Netherlands) where a national emission inventory is available, usually within one order of magnitude.

## Concentrations from diffuse sources (emissions patterns: agriculture and population)

The following maps show the spatial distribution of concentrations for groups of chemicals having the same emission pattern (EP) and dissipation half-life (DT50). One map is provided for each group of chemicals, within-group concentrations differing only for a scaling factor (the emission factors) as shown in the legends.

| 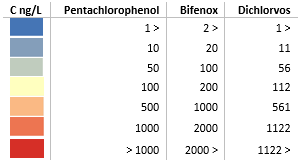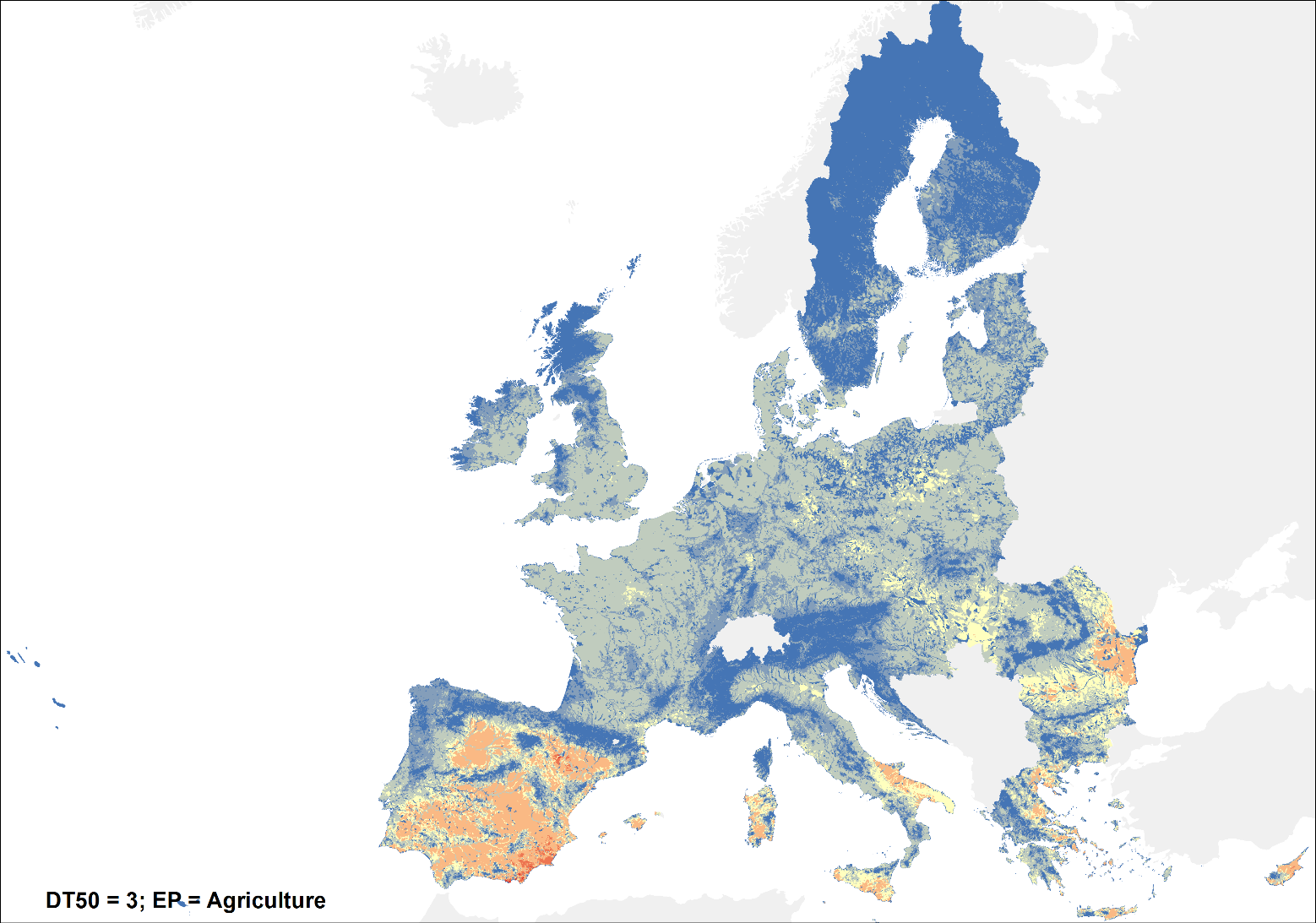 |
| --- |
| 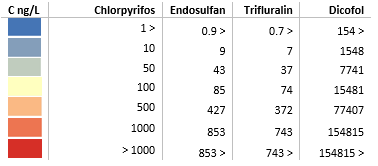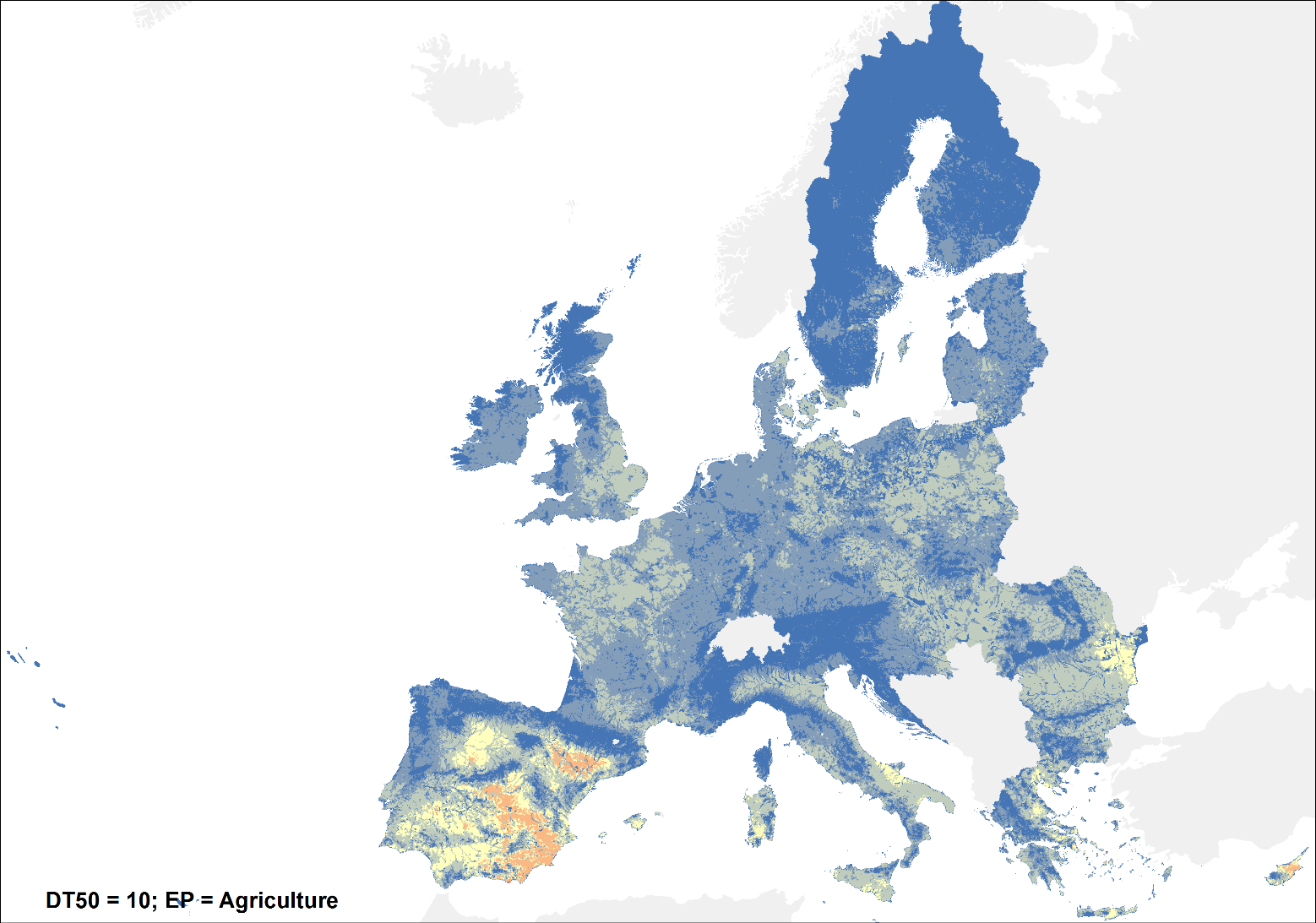 |
| 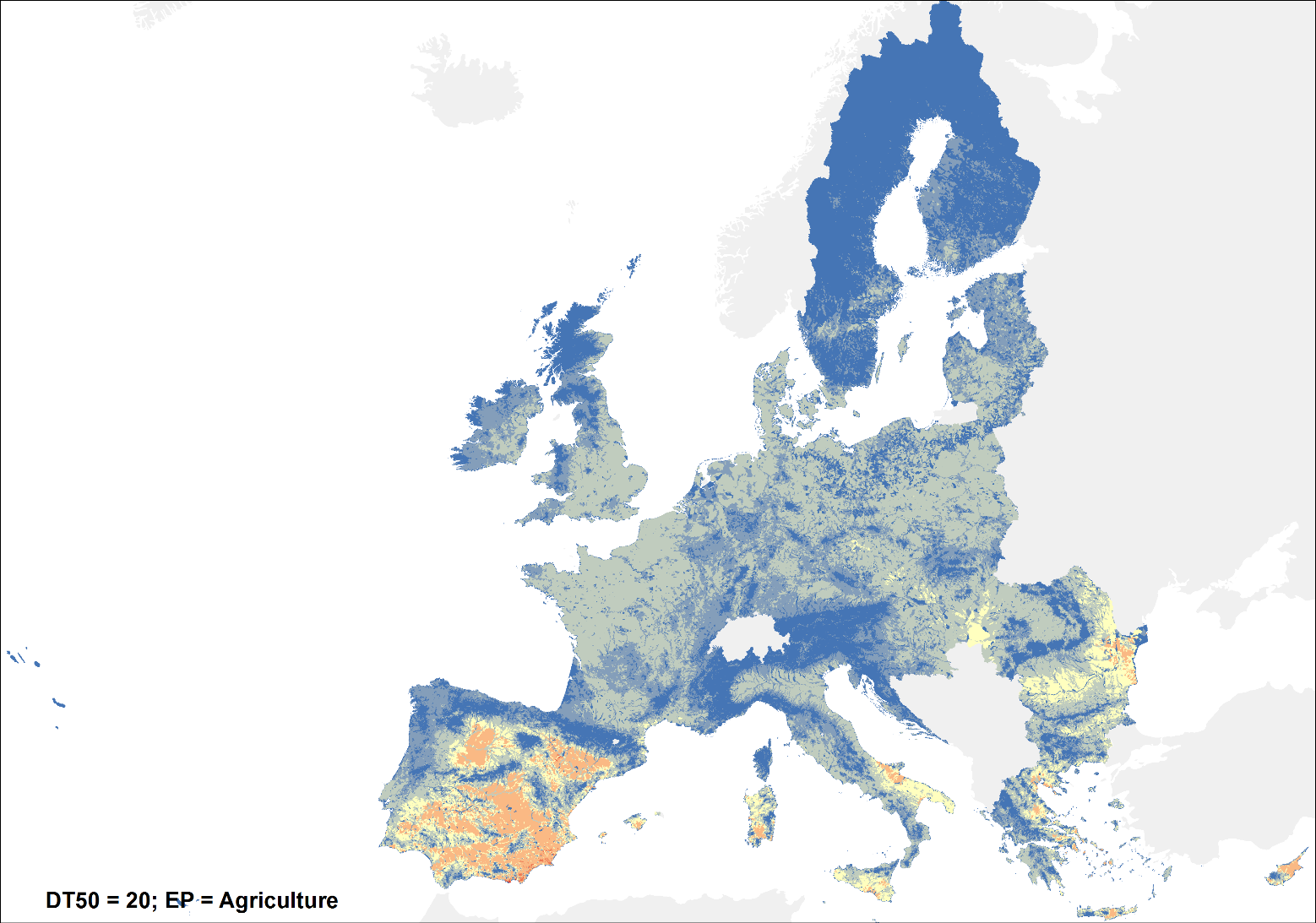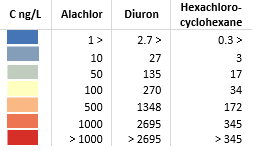 |
| 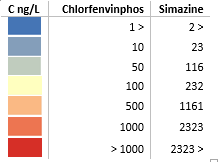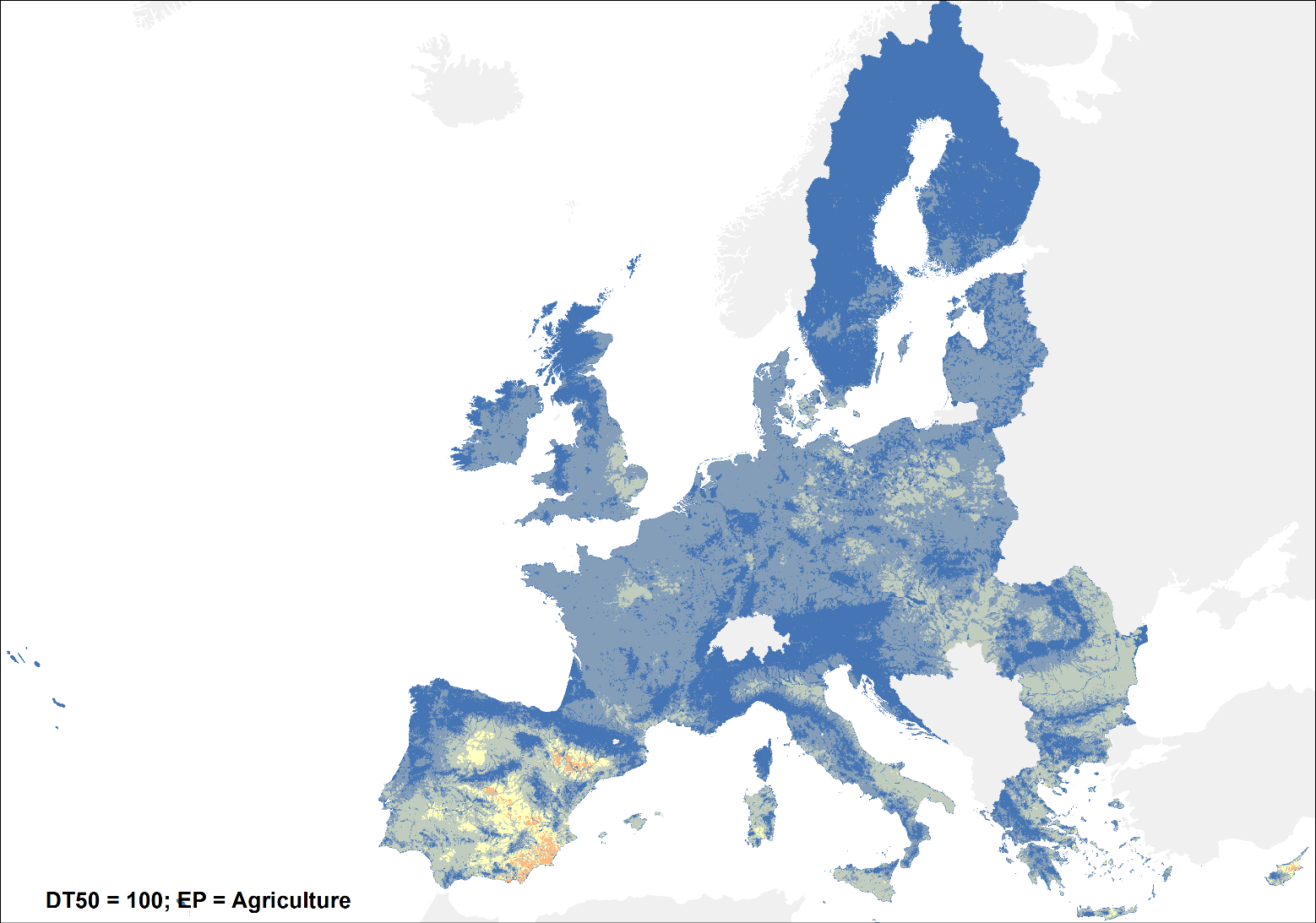 |
| 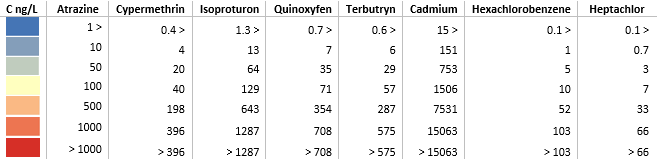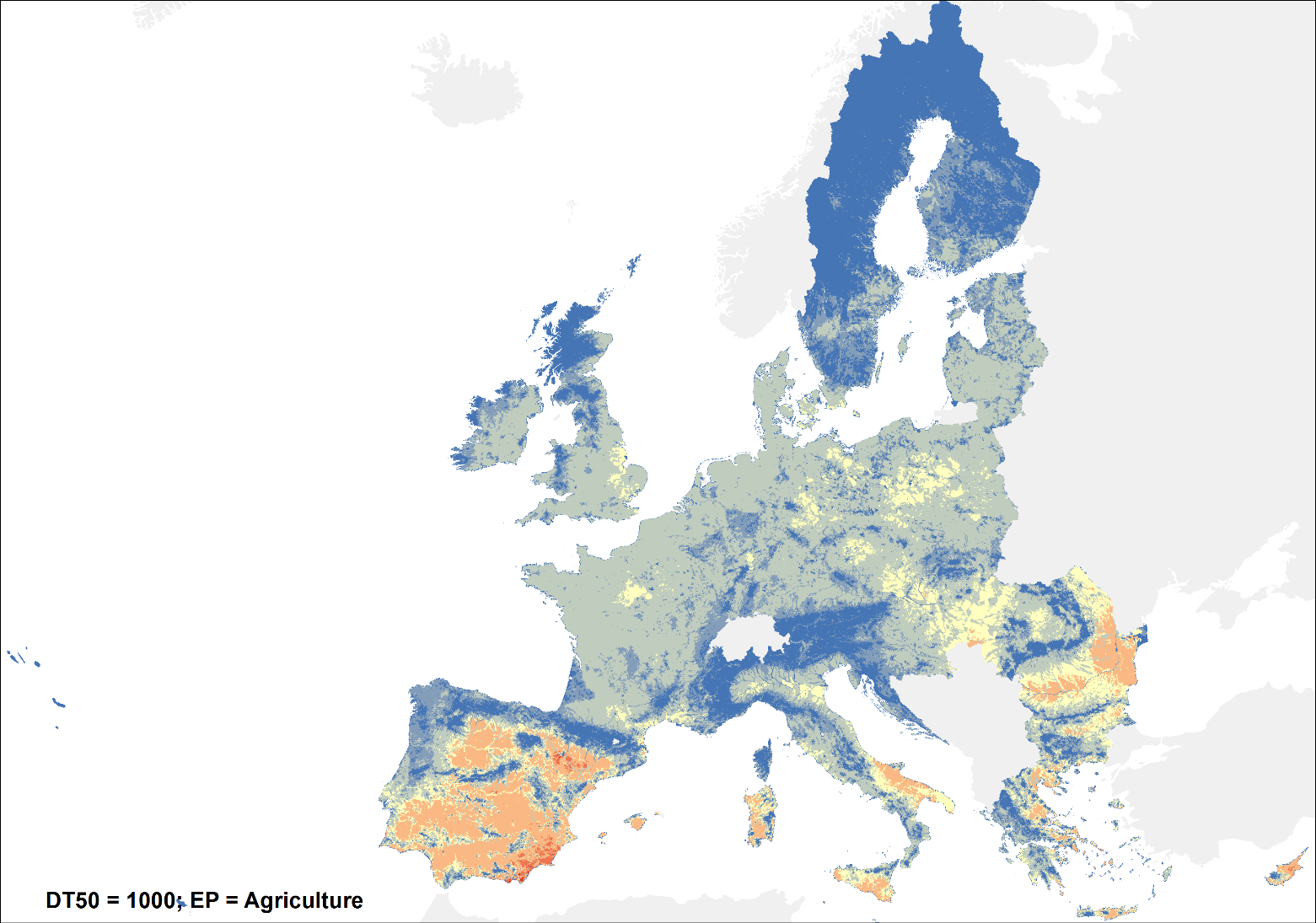 |
| 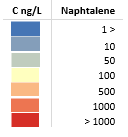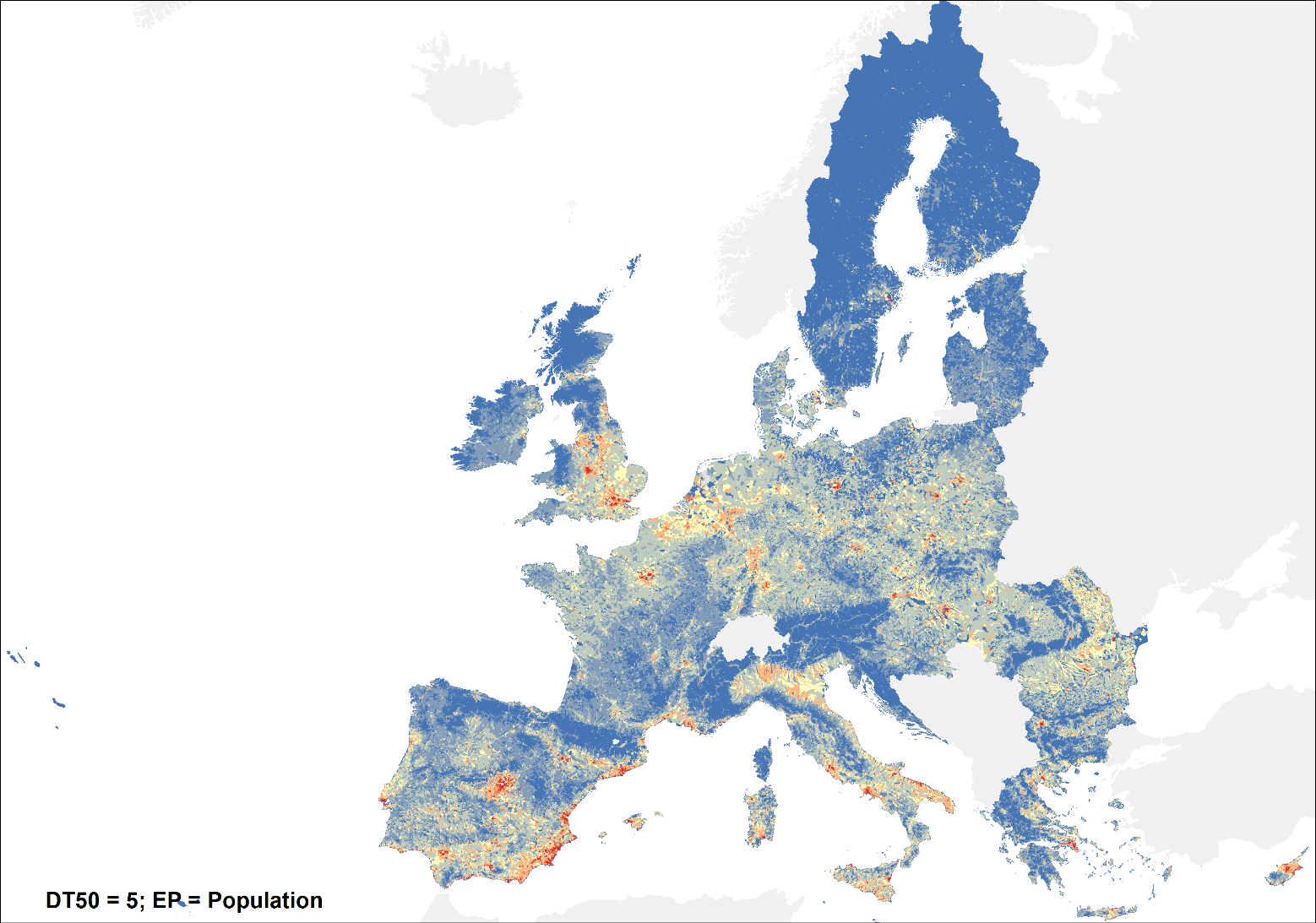 |
| 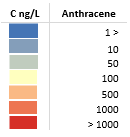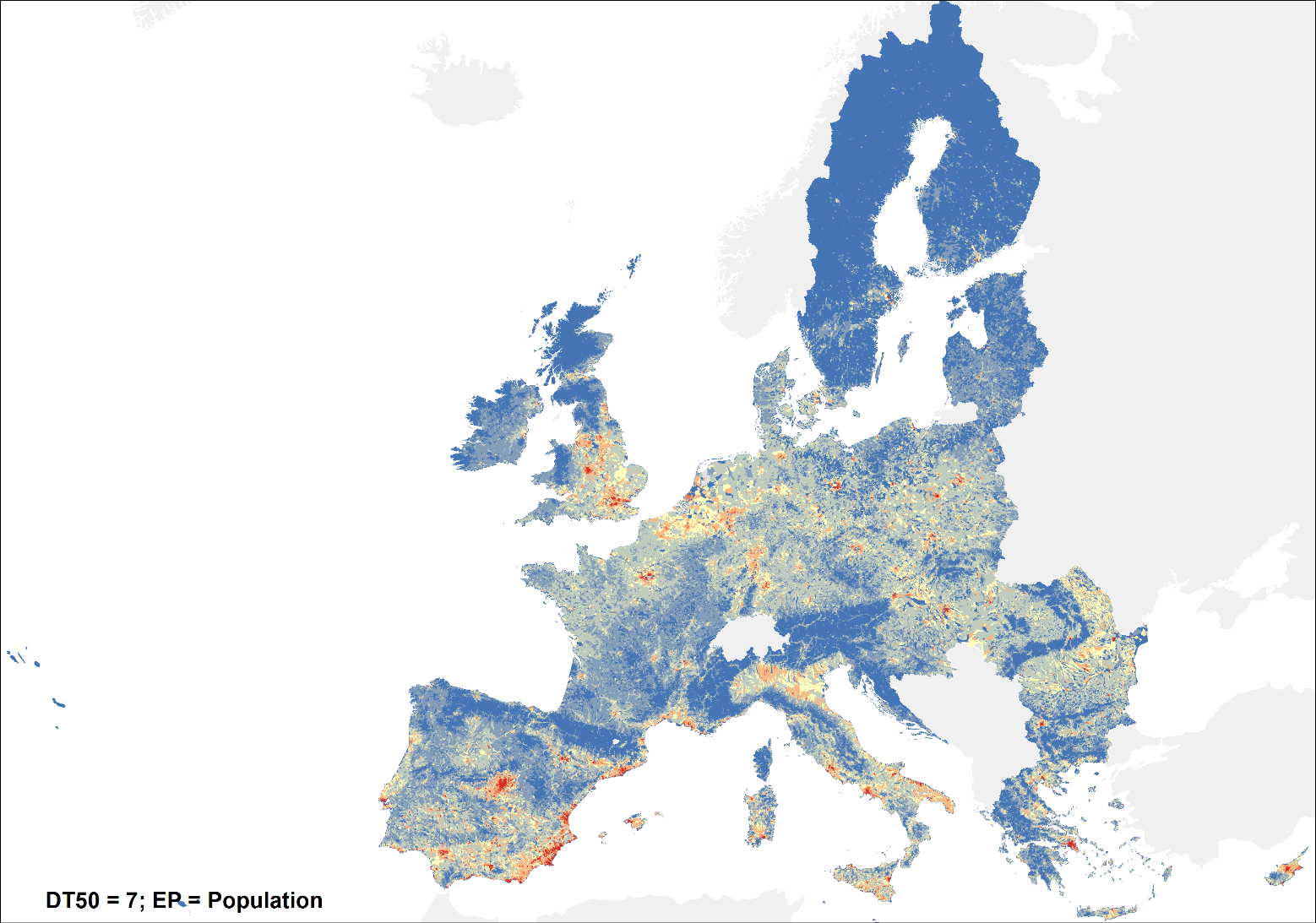 |
|  |
|  |
|  |
|  |

## Total loads to the European seas

The following table summarizes the loads of the 36 contaminants analysed here to the different European sea regions.

| **Substance** | **MAD** | **MAL** | **BAL** | **ABI** | **BLK** | **ACS** | **ANS** | **MIC** | **AMA** | **MWE** |
| --- | --- | --- | --- | --- | --- | --- | --- | --- | --- | --- |
| 1_2_dichloroethane | 45.4 | 34.9 | 3.5 | 164.1 | 42.0 | 11.9 | 40.8 | 17.9 | 11.5 | 72.7 |
| Alachlor | 3.3 | 1.6 | 0.0 | 5.1 | 3.2 | 1.2 | 1.3 | 1.0 | 0.6 | 1.9 |
| Anthracene | 0.9 | 0.8 | 4.4 | 2.9 | 4.2 | 0.2 | 0.6 | 0.3 | 0.2 | 1.2 |
| Atrazine | 9.6 | 3.2 | 0.1 | 11.6 | 8.7 | 6.2 | 2.8 | 2.2 | 1.1 | 8.2 |
| Benzene | 42.1 | 46.2 | 4.0 | 207.1 | 60.5 | 39.0 | 34.9 | 12.7 | 11.5 | 56.3 |
| Bifenox | 8.0 | 2.7 | 0.1 | 9.8 | 7.4 | 5.3 | 2.1 | 1.9 | 0.9 | 3.9 |
| Cadmium | 168.7 | 56.7 | 5.4 | 375.6 | 147.9 | 97.5 | 83.3 | 33.2 | 24.4 | 121.2 |
| Chlorfenvinphos | 1.7 | 0.7 | 0.0 | 2.3 | 1.7 | 0.7 | 0.7 | 0.5 | 0.2 | 0.9 |
| Chloroalkanes_C10_13 | 920.7 | 490.7 | 46.6 | 3277.8 | 1168.5 | 792.2 | 632.3 | 290.8 | 150.0 | 1052.5 |
| Chloroform | 28.1 | 24.0 | 2.0 | 113.1 | 25.0 | 7.5 | 28.5 | 9.9 | 5.6 | 54.0 |
| Chlorpyrifos | 1.6 | 0.9 | 0.0 | 2.8 | 1.4 | 0.5 | 0.8 | 0.5 | 0.3 | 1.0 |
| Cypermethrin | 3.7 | 1.2 | 0.0 | 4.5 | 3.4 | 2.4 | 1.0 | 0.9 | 0.4 | 1.8 |
| Di_2_ethylhexyl_phthalate | 143.7 | 87.6 | 7.8 | 534.5 | 171.1 | 115.6 | 92.0 | 41.8 | 21.6 | 166.2 |
| Dichloromethane | 99.4 | 84.5 | 8.2 | 379.4 | 92.4 | 27.2 | 89.4 | 41.2 | 25.7 | 169.5 |
| Dichlorvos | 4.7 | 1.6 | 0.0 | 5.8 | 4.4 | 3.1 | 1.2 | 1.1 | 0.6 | 2.3 |
| Diclofenac | 6.6 | 5.4 | 0.6 | 23.9 | 5.6 | 1.6 | 5.7 | 2.8 | 1.7 | 10.5 |
| Dicofol | 4.2 | 1.4 | 0.0 | 5.1 | 3.9 | 2.8 | 1.1 | 1.0 | 0.5 | 2.0 |
| Diuron | 10.6 | 4.6 | 0.3 | 14.6 | 8.5 | 3.2 | 3.7 | 2.8 | 1.6 | 8.2 |
| Endosulfan | 1.3 | 0.8 | 0.0 | 2.2 | 1.2 | 0.4 | 0.6 | 0.5 | 0.3 | 0.8 |
| Fluoranthene | 3.2 | 2.5 | 0.4 | 11.4 | 20.7 | 0.8 | 2.9 | 1.2 | 12.5 | 4.5 |
| Heptachlor | 0.4 | 0.4 | 0.0 | 0.8 | 0.4 | 0.1 | 0.3 | 0.2 | 0.1 | 0.3 |
| Hexachlorobenzene | 0.7 | 0.4 | 0.0 | 2.5 | 0.9 | 0.6 | 0.7 | 0.2 | 0.1 | 0.9 |
| Hexachlorobutadiene | 4.6 | 2.4 | 0.2 | 16.0 | 5.8 | 3.9 | 3.3 | 1.4 | 0.7 | 6.1 |
| Hexachlorocyclohexane | 1.1 | 0.5 | 0.0 | 1.8 | 1.1 | 0.4 | 0.4 | 0.4 | 0.2 | 0.7 |
| Isoproturon | 12.4 | 4.0 | 0.3 | 15.0 | 11.2 | 8.0 | 3.4 | 2.8 | 1.4 | 6.5 |
| Lead | 664.7 | 343.3 | 34.3 | 2315.4 | 984.9 | 680.1 | 514.5 | 193.8 | 107.3 | 779.3 |
| Mercury | 19.4 | 11.6 | 1.5 | 65.8 | 25.9 | 7.6 | 20.5 | 6.6 | 3.8 | 27.5 |
| Naphtalene | 6.4 | 10.6 | 13.0 | 33.7 | 5.6 | 1.2 | 7.3 | 2.6 | 1.7 | 10.6 |
| Nickel | 502.2 | 327.4 | 30.3 | 1977.0 | 730.9 | 210.9 | 525.6 | 198.0 | 121.2 | 696.5 |
| Nonylphenol | 104.1 | 89.3 | 7.1 | 435.6 | 129.3 | 87.7 | 139.9 | 32.0 | 29.7 | 118.5 |
| Pentachlorobenzene | 1.7 | 0.9 | 0.1 | 6.0 | 2.1 | 1.6 | 1.4 | 0.5 | 0.4 | 2.7 |
| Pentachlorophenol | 2.6 | 2.0 | 0.1 | 4.3 | 2.0 | 0.6 | 2.3 | 1.0 | 0.8 | 2.1 |
| Quinoxyfen | 6.6 | 2.2 | 0.0 | 8.1 | 6.2 | 4.4 | 1.7 | 1.5 | 0.8 | 3.2 |
| Simazine | 3.7 | 2.1 | 2.0 | 5.7 | 3.0 | 1.1 | 1.7 | 1.3 | 0.8 | 13.4 |
| Terbutryn | 5.4 | 1.8 | 0.0 | 6.6 | 5.0 | 3.5 | 1.4 | 1.3 | 0.6 | 2.6 |
| Tributyltin | 0.4 | 0.3 | 0.0 | 97.4 | 0.8 | 0.1 | 0.5 | 0.2 | 0.3 | 0.6 |
| Trifluralin | 1.2 | 0.7 | 0.0 | 1.9 | 1.1 | 0.4 | 0.6 | 0.4 | 0.2 | 0.7 |

Table 3 SI – loads to European sea regions (kg/day). MAD=Adriatic Sea ; MAL=Aegean-Levantine Sea ; BAL=Baltic Sea ; ABI=Bay of Biscay and the Iberian Coast ; BLK=Black Sea ; ACS=Celtic Seas ; ANS=Greater North Sea, incl. the Kattegat and the English Channel ; MIC=Ionian Sea and the Central Mediterranean Sea ; AMA=Macaronesia ; MWE=Western Mediterranean Sea

The following figures show the distribution of chemical loads conveyed by rivers to European coastal waters, for the 36 substances addressed here.

|  |  |
| --- | --- |
|  |  |
|  |  |
|  |  |
|  |  |
|  |  |
|  |  |
|  |  |
|  |  |
|  |  |
|  |  |
|  |  |
|  |  |
|  |  |
|  |  |
|  |  |
|  |  |
|  |  |

## Receiver-Operator Characteristics (ROC) curves of predicted and observed concentrations concentrations

The following graphs show the ROC curves built with reported environmental quality standard (EQS) exceedances from the 2^nd^ WFD river basin management plans. Both modelled concentrations, and pre- and post-2009 observed concentrations are used as predictors whenever possible. Missing graphs correspond to cases where IPChem does not contain data referred to stream segments with reported EQS exceedances.

| **Cumulate modelled C *vs* cumulate EQS exceedance** | **Cumulate obs. C (IPChem pre2009) *vs* cumulate EQS exceedance** | **Cumulate obs. C (IPChem post2009) *vs* cumulate EQS exceedance** |
| --- | --- | --- |
|  |  |  |
|  |  |  |
|  |  |  |
|  |  |  |
|  |  |  |
|  |  |  |
|  |  |  |
|  |  |  |
|  |  |  |
|  |  |  |
|  |  |  |
|  |  |  |
|  |  |  |
|  |  |  |
|  |  |  |
|  |  |  |
|  |  |  |
|  |  |  |
|  |  |  |
|  |  |  |
|  |  |  |
|  |  |  |
|  |  |  |
|  |  |  |
|  |  |  |
|  |  |  |

## Statistics of model errors by EU Member states

In the following, we display the scatter plots of computed (x-axis) and observed (y-axis) median concentrations (with error bars representing the 5 to 95 percentile range of both) in ug/L. the following table summarizes orientative statistical indicators (R^2^ and slope of the zero-intercept best-fit line) for 22 EU countries with sufficient monitoring data available in IPCheM before 2009. NL, LU, SE, MT, HR and CY are not included.

| **Country** | **R^2^** | **Obs/calc ratio (slope of zero-intercept best-fit line)** | **Notes** |
| --- | --- | --- | --- |
| AT | .75 | 38.32 |  |
| BE | .22 | 0.65 |  |
| BG | - | 0.49 |  |
| CZ | .68 | 2.81 |  |
| DE | .78 | 1.68 |  |
| DK | .52 | 1.18 |  |
| EE | .77 | 4.43 | Only Pb, Hg, Ni, Cd |
| EL | .59 | 17.80 |  |
| ES | .27 | 3.86 |  |
| FI | .48 | 90.21 |  |
| FR | .75 | 24.4 |  |
| HU | .48 | 0.71 |  |
| IE | .95 | 9.21 |  |
| IT | .58 | 10.44 |  |
| LT | - | 4.52 |  |
| LV | 1.00 | 7.98 | 2 substances only |
| PL | .71 | 3.62 |  |
| PT | .67 | 8.80 |  |
| RO | .96 | 3.80 |  |
| SI | .47 | 12.30 |  |
| SK | .83 | 5.51 |  |
| UK | .07 | 17.38 |  |

1. Zhang, L., W. R. Dawes, and G. R. Walker, 2001: Response of mean annual evapotranspiration to vegetation changes at catchment scale. Water Resources Research, 37 (3), 701{708, doi: 10.1029/2000WR900325. [↑](#footnote-ref-1)
2. Ntegeka, V.N., P. Salamon, G. Gomes, H. Sint, V. Lorini, J. Thielen, and M. Zambrano-Bigiarini, 2013: A European daily high-resolution gridded meteorological data set for 1990-2011, 2013; EUR 26408 EN; DOI: 10.2788/51262 [↑](#footnote-ref-2)
3. <https://ec.europa.eu/jrc/en/publication/eur-scientific-and-technical-research-reports/lisvap-evaporation-pre-processor-lisflood-water-balance-and-flood-simulation-model> [↑](#footnote-ref-3)
4. Part of the data on reported emissions in EU Member States used in this paragraph, although publicly available, were provided in a summarized form by European Commission consultants under the “Blue2” study (<http://ec.europa.eu/environment/blue2_en.htm>). The Blue2 project is gratefully acknowledged for this. [↑](#footnote-ref-4)
5. <http://www.adbpo.it/PianoAcque2015/Elaborato_02_PressioniImpatti_3mar16/PdGPo2015_Alleg26_Elab_2_3mar16/Rel_Inventario_30gen14_Definitiva.pdf> [↑](#footnote-ref-5)
6. <https://www.daera-ni.gov.uk/sites/default/files/publications/doe/WMU%20-%20WADE%20-%20Pollution%20Inventory%20for%20the%20final%20River%20Basin%20Plans%202015.pdf> [↑](#footnote-ref-6)
7. <http://www.emissieregistratie.nl/> [↑](#footnote-ref-7)
8. <http://www.eea.europa.eu/data-and-maps/data/waterbase-emissions-5> [↑](#footnote-ref-8)
9. <http://cdr.eionet.europa.eu/help/WFD/WFD_521_2016/Guidance/WFD_ReportingGuidance.pdf> [↑](#footnote-ref-9)
10. See e.g. EEA, 2018: Chemicals in European waters. Knowledge developments. EEA Report No 18/2018, doi: 10.2800/265080. [↑](#footnote-ref-10)
11. <http://www.helcom.fi/Lists/Publications/BSEP128.pdf> [↑](#footnote-ref-11)
